# Acute depletion of CTCF rewires genome-wide chromatin accessibility

**DOI:** 10.1186/s13059-021-02466-0

**Published:** 2021-08-24

**Authors:** Beisi Xu, Hong Wang, Shaela Wright, Judith Hyle, Yang Zhang, Ying Shao, Mingming Niu, Yiping Fan, Wojciech Rosikiewicz, Mohamed Nadhir Djekidel, Junmin Peng, Rui Lu, Chunliang Li

**Affiliations:** 1 Center for Applied Bioinformatics, St. Jude Children’s Research Hospital, 262 Danny Thomas Place, Memphis, TN 38105 USA; 2 Center for Proteomics and Metabolomics, St. Jude Children’s Research Hospital, 262 Danny Thomas Place, Memphis, TN 38105 USA; 3 Departments of Structural Biology and Developmental Neurobiology, St. Jude Children’s Research Hospital, 262 Danny Thomas Place, Memphis, TN 38105 USA; 4 Tumor Cell Biology, St. Jude Children’s Research Hospital, 262 Danny Thomas Place, Memphis, TN 38105 USA; 5 Computational Biology, St. Jude Children’s Research Hospital, 262 Danny Thomas Place, Memphis, TN 38105 USA; 6 Division of Hematology/Oncology, University of Alabama at Birmingham, 1824 6th Ave S WTI 510G, Birmingham, AL 35294 USA; 7 O’Neal Comprehensive Cancer Center, University of Alabama at Birmingham, 1824 6th Ave S WTI 510G, Birmingham, AL 35294 USA

**Keywords:** ATAC-seq, Auxin-induced degron, Chromatin accessibility, CTCF, Transcription factor, Proteomics, Phosphoproteomics

## Abstract

**Background:**

The transcription factor CTCF appears indispensable in defining topologically associated domain boundaries and maintaining chromatin loop structures within these domains, supported by numerous functional studies. However, acute depletion of CTCF globally reduces chromatin interactions but does not significantly alter transcription.

**Results:**

Here, we systematically integrate multi-omics data including ATAC-seq, RNA-seq, WGBS, Hi-C, Cut&Run, and CRISPR-Cas9 survival dropout screens, and time-solved deep proteomic and phosphoproteomic analyses in cells carrying auxin-induced degron at endogenous CTCF locus. Acute CTCF protein degradation markedly rewires genome-wide chromatin accessibility. Increased accessible chromatin regions are frequently located adjacent to CTCF-binding sites at promoter regions and insulator sites associated with enhanced transcription of nearby genes. In addition, we use CTCF-associated multi-omics data to establish a combinatorial data analysis pipeline to discover CTCF co-regulatory partners. We successfully identify 40 candidates, including multiple established partners. Interestingly, many CTCF co-regulators that have alterations of their respective downstream gene expression do not show changes of their own expression levels across the multi-omics measurements upon acute CTCF loss, highlighting the strength of our system to discover hidden co-regulatory partners associated with CTCF-mediated transcription.

**Conclusions:**

This study highlights that CTCF loss rewires genome-wide chromatin accessibility, which plays a critical role in transcriptional regulation.

**Supplementary Information:**

The online version contains supplementary material available at 10.1186/s13059-021-02466-0.

## Introduction

CCCTC-binding factor (CTCF) is a highly conserved zinc finger–containing transcription factor known as “the master weaver of the genome” [1]. It is the most extensively studied regulator of three-dimensional (3D) chromatin architecture. CTCF was initially identified by its ability to regulate MYC [2] and later revealed to function as an insulator at the imprinted *H19*-*IGF2* and β-hemoglobin loci [3, 4]. CTCF-binding occupancy is highly enriched at many known chromatin architecture elements, including chromatin loop anchors and topologically associated domain (TAD) boundaries [5, 6]. In general, CTCF-mediated chromatin loops favor a pattern in which two CTCF-binding sites are located in a convergent manner [7–9], and the cohesin complex–dependent loop extrusion model is proposed to support this pattern [10–14]. At the molecular level, the N-terminal domain of CTCF interacts with the cohesin complex to facilitate chromatin loop formation by protecting cohesin against loop release in both human and mouse cells [15, 16]. Moreover, CTCF is indispensable for genome-wide TAD and intra-TAD loop formation in a CTCF acute depletion cell model as well as other gene knockout models [17–21].

Despite a global reduction of chromatin interactions upon CTCF loss, its observed effect on mRNA expression by RNA-seq is not dramatic [19, 22]. Thus far, the discrepancy remains elusive. To better understand how CTCF-binding occupancy contributes to transcription regulation, we systematically conducted multi-omics studies with a particular focus on chromatin accessibility. We previously established a genetically engineered cellular tool in an *MLL*-rearranged human B cell lymphoblastic leukemia (B-ALL) cell line SEM, allowing acute depletion of CTCF protein through the auxin-inducible degradation (AID) [19] system. CTCF protein degradation was acutely induced by degron in the CTCF-AID cellular model, and data were collected from a series of next-generation sequencing techniques: assay for transposase-accessible chromatin using sequencing (ATAC-seq), whole-genome bisulfite sequencing (WGBS), transcriptome RNA sequencing (RNA-seq), Hi-C chromosome conformation capture, time-solved deep proteome and phosphoproteome profiling, and profiling of genome-wide CTCF occupancy with cleavage under targets and release using nuclease (Cut&Run). As a result, we found that acute CTCF depletion directly altered genome-wide chromatin accessibility. The most differentially altered ATAC-seq peaks overlapped with adjacent CTCF-binding sites, confirming the likely direct link between CTCF occupancy and its surrounding chromatin accessibility. The increased ATAC-seq peaks were significantly associated with increased transcription at promoter regions and insulator sites. The decreased peaks were dramatically enriched at regions with DNA loops. We used integrated data analysis to identify 67 novel CTCF-mediated insulators at noncoding regions distal to target genes. CRISPR-mediated disruption of a conserved CTCF-binding site upstream of *BLCAP* induced transcription, consistent with the data collected from acute depletion of CTCF. Last, we discovered 40 CTCF co-regulatory partners in controlling a different subset of downstream genes, of which many CTCF co-regulators exhibited alterations to their downstream genes’ expression but did not show changes at their expression levels, highlighting the advantage of our system to discover hidden co-regulatory partners associated with CTCF-mediated transcription. In summary, we propose a model that acute CTCF loss rewires genome-wide chromatin accessibility, which plays an essential role in transcription regulation.

## Results

**Fig. 1:**
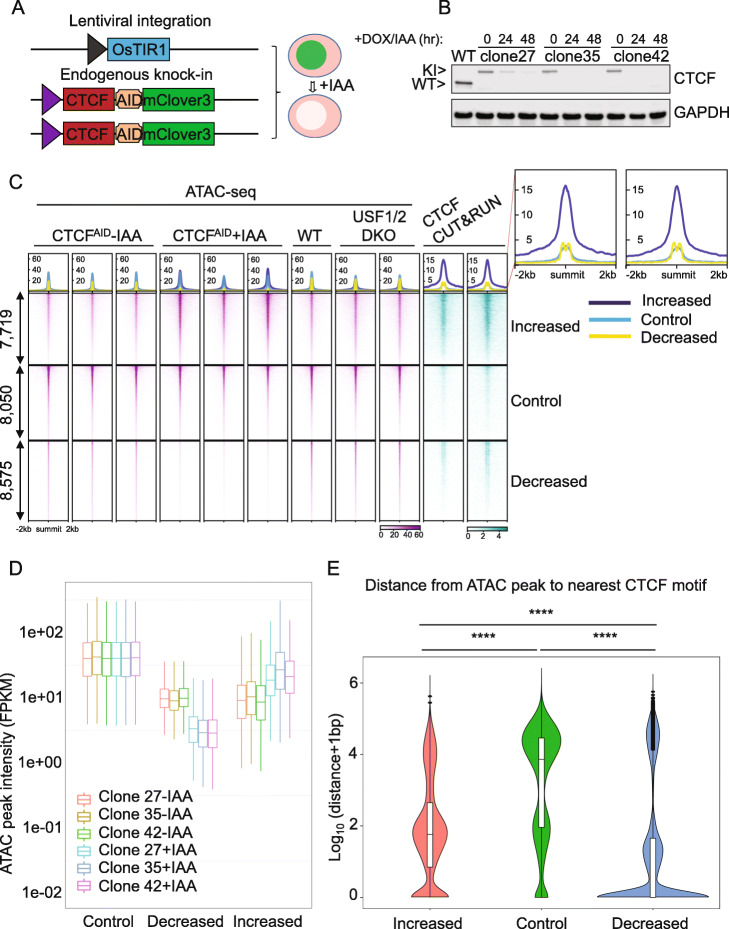
Acute CTCF depletion alters chromatin accessibility. **A** Schematic diagram of the auxin-inducible degron model for tagging endogenous CTCF in MLL-rearranged B-ALL SEM cells [19]. SEM cells were transduced with a lentivirus expressing a transgene encoding doxycycline-inducible OsTIR1. The miniAID-mClover3 cassette was inserted in both endogenous alleles in frame with CTCF. In the presence of doxycycline and auxin (IAA), forced expression of OsTIR1 combines with Skp1/Culin/F-box ubiquitin ligase components to form a functional SCF/OsTIR1 E3 ubiquitin ligase complex that rapidly degrades miniAID-mClover3 fusion proteins. **B** CTCF immunoblots confirmed successful degradation of the CTCF-miniAID-mClover3 fusion protein translated from the knockin samples. **C** Heatmap centered at ATAC-seq nucleosome-free peak summits for differential accessibility regions (DARs). CTCF Cut&Run data indicate that the increased DARs have the strongest CTCF binding, whereas the decreased DARs have weaker overall CTCF binding with double summits (yellow average profile curve denoted in the enlarged area). ATAC-seq profiling of *USF1/2* double-knockout SEM cells served as negative controls. **D** Boxplots of ATAC-seq peak intensities (fragments per kb of peaks per million reads mapped, FPKM) at control NFRs, decreased DARs, and increased DARs (DARs were FDR-corrected *p* value < 0.05 and |log2fold change| > 1. **E** Violin plots of the overall distance of the closest CTCF motif to decreased DARs increased DARs, or control regions. The physical distance at the *y*-axis was calculated by log_10_ (distance + 1 bp). **** *p* value < 0.0001 in Wilcox test

Acute CTCF depletion alters chromatin accessibility. Although many crude loss-of-function models targeting CTCF have been extensively studied [17, 23–28], accumulated secondary effects were inevitably observed. The acute protein degradation system was recently developed as an essential tool to study direct transcriptional regulation [29, 30]. We previously delivered bi-allelic miniAID-mClover3 tags into the human endogenous *CTCF* locus and generated three clones of CTCF^AID^ cells [19]. In the presence of doxycycline and auxin (IAA), forced expression of OsTIR1 connects with Skp1/Culin/F-box (SCF) ubiquitin ligase components and rapidly degrades the CTCF fusion protein (Fig. 1A). This degradation was reversible after doxycycline and IAA were removed entirely from the culture medium. We confirmed that CTCF was efficiently degraded by immunoblotting three single-cell derived clones treated with IAA for 24 h (Fig. 1B and Additional file 1: Fig. S1A), similar to our previous observation at 48 h [19].

To investigate the genome-wide chromatin accessibility change in response to CTCF loss, we performed ATAC-seq in CTCF^AID^ cells treated with or without IAA. Wild-type SEM cells and SEM cells with CRISPR double knockdown of two unrelated targets, USF1 and USF2, were included as additional controls (Additional file 1: Fig. S1B). We analyzed the data by using nucleosome-free reads at reproducible nucleosome-free regions (NFRs). In total, we identified 8876 significantly decreased differential accessibility regions (DARs), and 8042 significantly increased DARs with a false discovery rate (FDR) controlled *p* value of 0.05 and a 2-fold change as a cutoff. In addition, we designated 8440 NFRs as control NFRs because they exhibited no significant difference when comparing CTCF^AID^ untreated cells to CTCF^AID^ cells treated with IAA (*p* value > 0.5 and fold change < 1.05 as a cutoff) (Additional file 2: Table S1). To improve resolution, we extracted the best peak summits for each peak by using the summit (called MACS2) closest to the peak center among replicates. We also excluded peak summits if the two summits were too close to each other to avoid potential artifacts. After that, we used the remaining peak summits (8575 decreased DARs, 7719 increased DARs, and 8050 control regions that did not exhibit chromatin accessibility changes upon CTCF loss) to generate heatmaps and mean profiles for each sample along with CTCF Cut&Run profiles. Both the heatmaps and peak intensity results confirmed that the DARs were highly reproducible (Fig. 1C, D and Additional file 1: Fig. S1C). Moreover, given that the USF1/2 and CTCF-binding consensus motifs are entirely different, we also included DARs collected from USF1/2 knockdown to test if CTCF-associated DARs were specific to CTCF loss. As expected, the mean profiles in the heatmaps for these DARs exhibited a consistent trend with CTCF loss but remained unchanged in *USF1/2* knockout SEM cells, suggesting that these DARs exhibit a CTCF-dependent signature.

To survey which transcription factors (TFs) are associated with these DARs, we performed de novo motif analysis (Homer v4.9.1) [31]. Our data suggest that the CTCF motif is the top enriched motif for decreased DARs (Additional file 1: Fig. S2A) but not increased DARs (Additional file 1: Fig. S2B). Gene-based chromatin immunoprecipitation enrichment analysis (ChEA) analysis using the EnrichR server [32] also confirmed that the CTCF binding sites from various cell types were enriched in the decreased DARs (Additional file 1: Fig. S2C, S2D).

The DARs exhibited highly distinct patterns in the CTCF Cut&Run profiles. For example, the increased DARs demonstrated the most robust CTCF binding in parental cells, whereas both the decreased DARs and control regions exhibited weaker CTCF-binding affinity. Moreover, a rare double-summit pattern of CTCF binding colocalized with the decreased DARs (Fig. 1C). To further confirm this pattern, we used k-means clustering with a smaller window (500 bp). The heatmaps confirmed that both increased DARs and control NFRs (Additional file 1: Fig. S3A, S3B) do not exhibit the double-summit pattern for CTCF binding, which was again observed in the decreased DARs (Additional file 1: Fig. S3C). We next determined the physical distance between these DARs with their nearest CTCF motifs. Our results indicated that the decreased DARs were more adjacent to the nearest CTCF motifs (*p* value < 2.2 × 10^–16^, Wilcoxon test), with a median distance very close to 0 bp. In contrast, the increased DARs were significantly distant (~ 100 bp) from the CTCF motifs than the decreased DARs (*p* value < 2.2 × 10^–16^) but were significantly closer than the control regions (~ 10 kb) (*p* value < 2.2 × 10^–16^) (Fig. 1E).

We then determined whether the CTCF-binding profiles in the double-summit pattern represented two equivalent binding events for each ATAC-seq summit or arose from unequal CTCF binding randomly assigned to the ATAC-seq summit with equal chance. We re-oriented the NFR summits according to the strand of the nearest CTCF motifs. If multiple motifs occurred for the same peak, we assigned the strand to the motif with the highest position weight matrix score. Using this strategy, we found that CTCF binding was biased to regions upstream of the CTCF motifs (Additional file 1: Fig. S4A). The biased signature occurred in clusters 1, 2, and 3, where the CTCF-binding affinity was more robust, but not for cluster 4, where the CTCF-binding affinity was much weaker (Additional file 1: Fig. S4B). These data confirmed the notion that the double-summit pattern was due to unequal CTCF binding, which was randomly assigned to the ATAC-seq summit with equal chance.

**Fig. 2:**
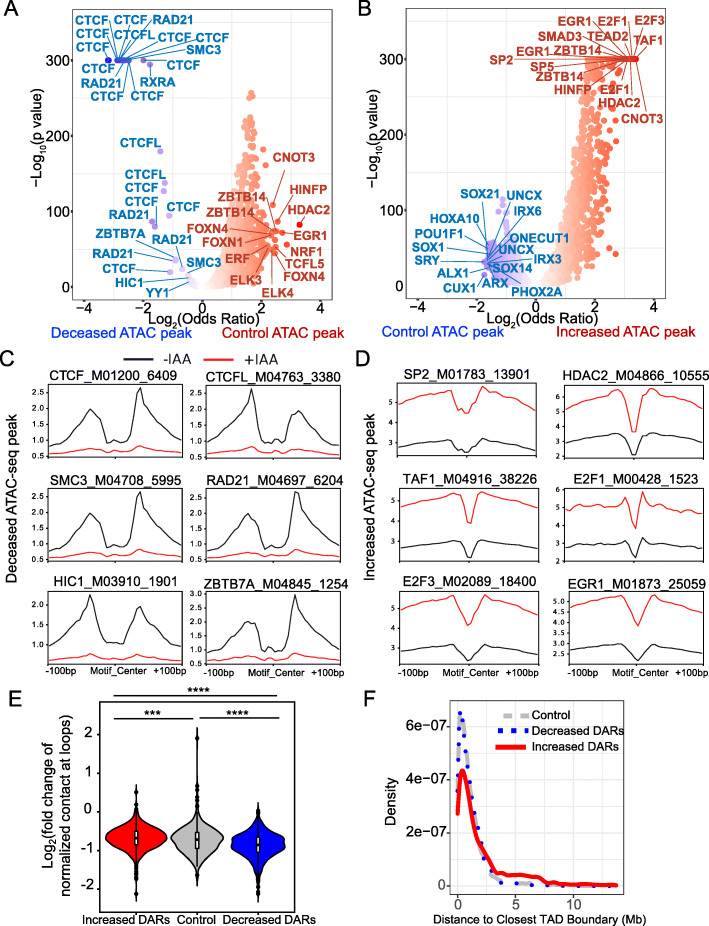
Signature of chromatin accessibility changes upon acute CTCF loss. **A** Volcano plots of motif enrichment analysis of ATAC-seq comparing control nucleosome-free regions (NFRs) versus decreased differential accessibility regions (DARs). Fisher exact tests comparing motif frequency generated the *p* values and odds ratios. Each dot represents a motif in the database. Dots in the top left corner indicate motifs enriched for decreased DARs. **B** Volcano plots of motif enrichment analysis of ATAC-seq for increased DARs versus control NFRs. Fisher exact tests comparing motif frequency generated the *p* values and odds ratios. Each dot represents a motif in the database. Dots in the top right corner indicate motifs enriched for increased DARs. **C** ATAC-seq footprint profiles of the top motifs enriched for decreased DARs. Ratios between the nearest summit and the center indicate the probability of motifs protected from Tn5 insertion. Stronger dips in the center indicate higher confidence in binding. The height of the nearest summit to the center indicates chromatin accessibility. The number of matched motifs we used for each footprint profiling was attached at the end of each TF motif. **D** ATAC-seq footprint profiles of the top motifs enriched for increased DARs. **E** Log_2_ fold change of normalized contact numbers from Hi-C (+IAA versus -IAA) at loops grouped by whether the loop anchors overlapped the DARs or control NFRs. ***p value < 0.001; ****p value < 0.0001, Student’s *t* test. **F** Density plot measures the distance from DARs to the closest TAD boundaries

The chromatin accessibility signature changes upon acute CTCF loss. We comprehensively characterized the TF occupancy profiles in response to the chromatin accessibility alterations upon CTCF loss. We scanned all of the annotated TF motifs in the motif database TRANSFAC [33] and scored their enrichment frequency among three categories: decreased DARs, increased DARs, and control regions. As expected, the top TFs enriched for decreased DARs were CTCF and cohesin complex proteins (SMC3 and RAD21) (Fig. 2A). Foot-printing profiling with Tn5 insertion sites confirmed that their motifs (e.g., CTCF, SMC3, and RAD21) were protected at the motif center (Fig. 2C). These results collectively suggest that the decreased DARs reflect the impact of CTCF loss.

CTCF motifs were also enriched for the increased DARs (Additional file 3: Table S2), consistent with our distance-to-CTCF-motif analysis (Fig. 1E). However, the most enriched TFs were not CTCF motifs. Instead, many were general transcription factors (GTFs) associated with active transcription (Fig. 2B, D and Additional file 1: Fig. S5A). We hypothesized that the regulation of these DARs is most likely associated with the repressive function of CTCF. In wild-type cells, CTCF functions through a repressive role at these DARs by blocking GTF binding. Therefore, CTCF depletion allows more GTFs to bind to target gene promoters, thereby increasing chromatin accessibility. Alternatively, loss of CTCF may increase chromatin accessibility to allow more GTFs to bind. Indeed, these regions were more likely to be annotated as gene promoters (Additional file 1: Fig. S6), which is consistent with hidden Markov modeling chromatin state characterization of the acute myeloid leukemia cell line K562 (Additional file 1: Fig. S7) [34, 35].

Although the CTCF and cohesin motifs were enriched for increased DARs and decreased DARs, their foot-printing profiles in the increased DARs exhibited distinct patterns (Additional file 1: Fig. S5B). In contrast with the Tn5-protected motif centers in the decreased DARs, the proximal flanking regions surrounding these motifs were more protected than the centers of these motifs, consistent with the tandem CTCF motifs (2xCTSes) associated with active promoters and enhancers [36]. We found that 1244 of 8042 (15.4%) increased DARs overlapped with 2xCTSes, which were more enriched than the control regions (204 of 8440, 2.4%) (Fisher exact test *p* value < 2.2 × 10^–16^, odds ratio = 7.39) or decreased DARs (123 of 8876, 1.38%). These 2xCTSes are proposed to regulate chromatin loops [36]. Therefore, the DARs we observed may directly associate with chromatin loops. We next separated the loops into three groups and plotted their normalized chromatin contact numbers with different criteria (Knight–Ruiz normalization) [37]. The loops overlapping with the increased DARs exhibited more intra-chromatin contacts, whereas the loops overlapping with the decreased DARs exhibited fewer intra-chromatin contacts (Additional file 1: Fig. S8). All three groups showed reduced contacts upon CTCF loss. However, the lost contacts at loops overlapping the decreased DARs (log_2_ fold change CTCF loss vs. control) were significantly more than the loops overlapping the control NFRs (Wilcoxon test *p* value = 1.9 × 10^–08^) and increased DARs (Wilcoxon test *p* value < 2.2 × 10^–16^). The lost contact of the loops overlapping control NFRs were also significantly stronger (*p* value = 0.0034) than the loops overlapping with the increased DARs, although the difference appeared marginal (Fig. 2E). Collectively, loop formation may only reflect CTCF binding status rather than direct regulation of chromatin accessibility. However, weaker distal loops appeared more vulnerable to CTCF loss. At last, we sought to explore whether these DARs were associated with TAD boundaries. We called high confidence TAD boundaries in control and CTCF-deficient cells independently and merged the TAD boundaries as putative reference boundaries (702 regions) collected from Hi-C data [19]. We found that the control ATAC-seq peaks and decreased DARs have a similar distribution of distance to TAD boundaries, while increased DARs were overall more likely to be found distal from TAD boundaries (Fig. 2F). It is known that TAD boundaries are enriched in CTCF binding sites and transcriptionally active genes, including housekeeping genes. The physical location of CTCF occupancy seems to be closely associated with its transcriptional regulation.

GC-rich CTCF binding is highly associated with DNA methylation status [38, 39]. However, the role of CTCF on DNA methylation regulation is still controversial [17, 40, 41]. We hypothesized that the acute CTCF depletion cell model is best for determining the immediate response of genome-wide DNA methylation. Surprisingly, when we generated DNA methylation profiles by WGBS, we did not observe genome-wide DNA methylation changes upon acute CTCF depletion, which was confirmed by estimation for each CpG site. Unlike the ATAC-seq and CTCF Cut&Run profiles, the DNA methylation level surrounding DARs did not differ between control and CTCF loss (Additional file 1: Fig. S9A). Next, we called the differentially methylated regions (DMRs) and found that only 49 regions that passed the threshold could be considered significant (Additional file 1: Fig. S9B). Further examination of the motifs enriched for these DMRs did not reveal any role for CTCF or cohesin (Additional file 1: Fig. S9C), indicating that these DMRs are not directly associated with CTCF occupancy (Additional file 4: Table S3). Together, our findings indicate that acute CTCF loss does not affect genome-wide DNA methylation in SEM cells.

**Fig. 3:**
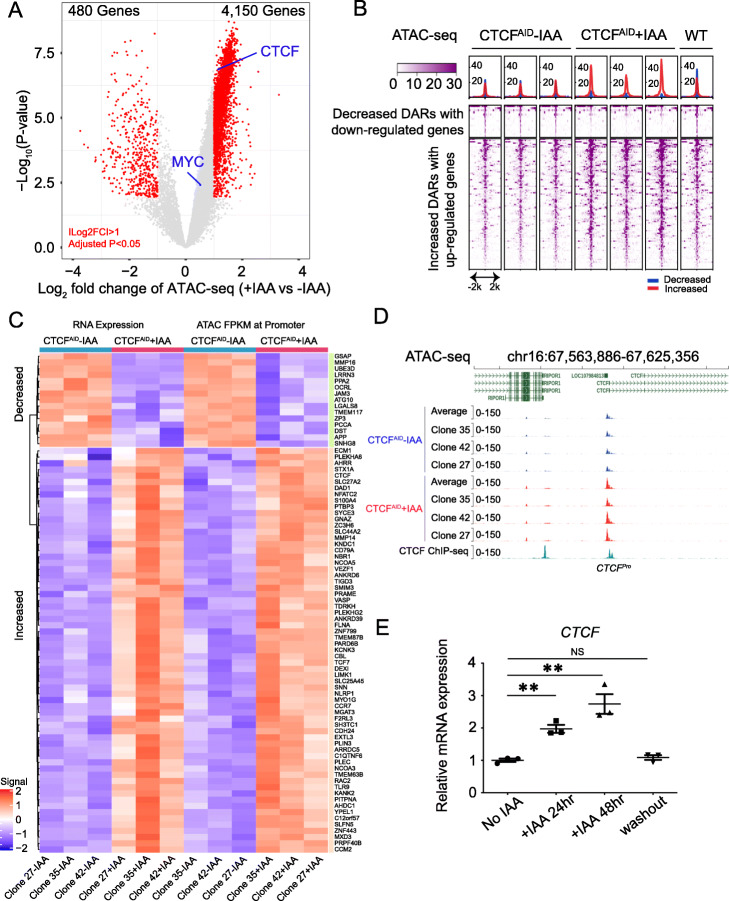
CTCF-dependent chromatin accessibility regulates gene expression either through a promoter or enhancer-promoter loops. **A** Volcano plots of chromatin accessibility changes at gene promoters. Differential ATAC-seq peaks were defined at cutoffs of ILog_2_ fold change > 1 and an adjusted *p* value < 0.05. A total of 480 genes with decreased promoter ATAC signals and 4150 genes with increased promoter ATAC signals are shown. **B** Genomic heatmap of normalized ATAC-seq signals centered at ATAC-seq DARs with consistent gene expression changes. **C** Heatmap of expression levels and ATAC-seq signals at promoters of genes with consistent changes upon CTCF depletion (*z*-score was calculated independently by either expression or ATAC-seq signal). **D** Screenshot of the *CTCF* promoter with ATAC-seq tracks in CTCF^AID^ cells with or without IAA treatment. Averaged tracks are combined analyses of three individual clones. **E** Q-PCR analysis of *CTCF* mRNA expression after CTCF protein depletion for 24 and 48 h and washout from three biological replicates of clones 27, 35, and 42

CTCF-dependent chromatin accessibility regulates gene expression either through a promoter or enhancer-promoter loops. Although CTCF is indispensable for gene regulation at some loci, such as *H19*-*IGF2*, β-hemoglobin, protocadherin cluster, and *TP53* [3, 4, 42, 43], it is unclear whether this transcriptional regulation is a direct effect of CTCF or whether chromatin accessibility also plays a role. Because increased DARs were enriched for gene promoters, we assigned NFRs to genes if they overlapped with the gene promoters (transcriptional start site [TSS] ± 2 kb). Volcano plots revealed that more gene promoters were assigned to the increased DARs than to the decreased DARs (Fig. 3A). We next counted the number of genes assigned to the DARs that also exhibited differential transcription by RNA-seq upon IAA treatment. Fisher exact tests indicated that more decreased DARs are often associated with downregulated genes (*p* value = 8.607 × 10^–07^, odds ratio = 5.45) and more increased DARs are often associated with more expression (*p* value = 2.217 × 10^–15^, odds ratio = 0.16). For the genes shown consistent changes, we further reviewed the ATAC-seq signals assigned to their promoters and confirmed that the pattern was as expected (Fig. 3B). We also made a gene-based heatmap using expression level and ATAC-seq signal *z*-score (*z*-score was calculated independently for expression or ATAC-seq changes) and confirmed the reproducible pattern (Fig. 3C). To further assure that the threshold criteria did not bias these observations, we compiled gene sets with the top-ranked genes (i.e., top 100, 200, 500, or 1000) and combined them with the gene sets downloaded from msigdb (v7 )[44]. We performed gene set enrichment analysis (GSEA) of the combined gene sets against the log_2_ fold change of RNA-seq data with and without IAA treatment. Our results indicated that the decreased DARs were associated with downregulated genes and that the increased DARs were associated with upregulated genes (Additional file 1: Fig. S10A-D). We also investigated a set of deregulated genes that showed no changes in promoter and/or enhancer accessibility but had CTCF binding in the vicinity. Out of 219 upregulated genes, only eight gene promoters have ATAC-seq control peaks and CTCF binding occupancy. Out of 269 downregulated genes, twenty-one gene promoters (24 ATAC-seq peaks) have ATAC-seq control peaks and CTCF binding occupancy. Therefore, we concluded that the transcriptional change signatures associated with DARs directly responded to acute loss of CTCF.

The chromatin accessibility at the CTCF gene promoters also increased upon CTCF loss (Fig. 3D), which was further validated by quantitative PCR (Q-PCR) (Fig. 3E). These data suggest that CTCF can repress itself to maintain optimal expression levels. For the genes known to be downregulated upon CTCF loss, such as *MYC* [19], we did not detect statistically significant chromatin accessibility changes at the promoter region (Additional file 1: Fig. S10E). Actually, the *MYC* promoter has four reproducible ATAC-seq peaks. The one located closest to *MYC* TSS was significantly increased and passed FDR 5%, but did not pass a twofold change. Another one downstream of *MYC* TSS was significantly increased but did not pass FDR 5%. The other two were not significantly increased or decreased but were neither included in control peaks since we required a cutoff of *p* value > 0.5 for control peaks. However, we found that several CTCF conserved binding sites exhibited decreased DARs at a distal enhancer residing ~ 1.8 Mb from the *MYC* gene, which we previously identified from Hi-C data [19] and have shown regulates *MYC* through CTCF-dependent enhancer-promoter looping in SEM cells (Additional file 1: Fig. S10F). It is known that the transcriptional regulation of *MYC* is vulnerable to CTCF in the MLL-rearranged leukemia cell line SEM due to addiction of enhancer/promoter looping regulation at three-dimensional chromatin architecture [19]. Although the chromatin accessibility at the *MYC* promoter remains unchanged upon CTCF loss, a significant reduction of ATAC-seq signals at the CTCF binding sites located in the distal *MYC* enhancer was observed. It is possible that the CTCF loss affects the binding occupancy of enhancer-bound TFs and epigenetic regulators, further reducing the transcription of *MYC* in the three-dimensional context of the enhancer/promoter loop. Therefore, while the promoter region remained open, the chromatin landscape of the distal enhancer region became less accessible and, together with CTCF loss, could play a role in controlling the distal enhancer-promoter loop formation that regulates *MYC* transcription.

### Integrated analysis to explore putative insulator CTCF-binding sites

**Fig. 4:**
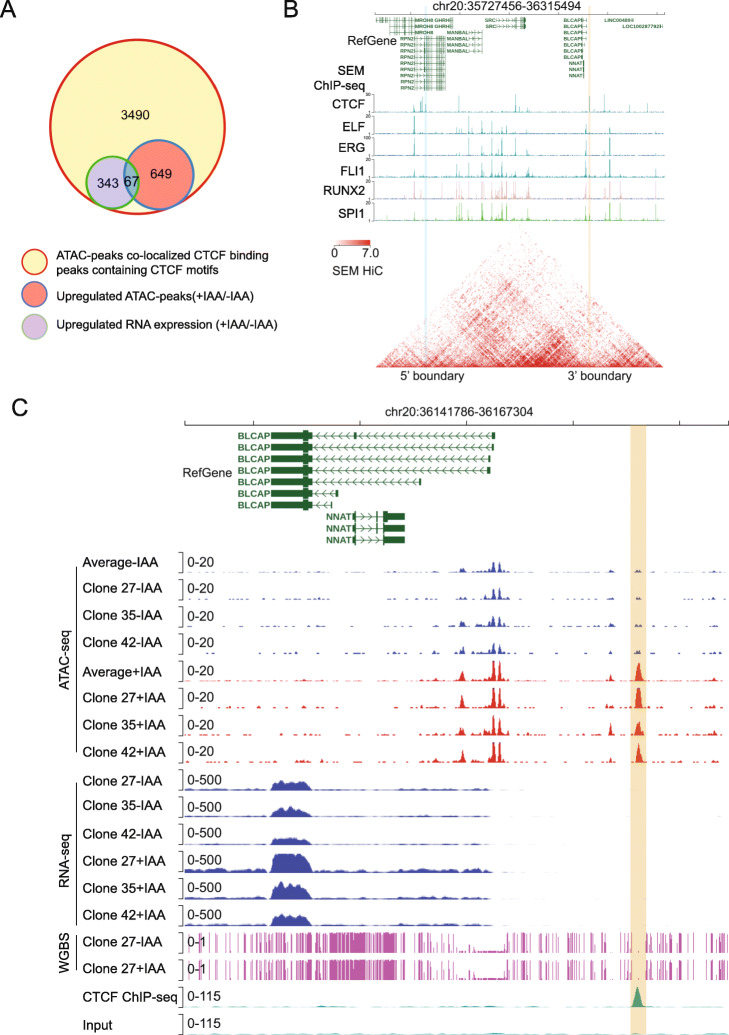
Integrated analysis of putative insulator CTCF-binding sites. **A** Scheme of integrative analysis to identify 67 putative CTCF insulators at target genes. **B** Screenshot of chromatin status characterization of the *BLCAP* gene and adjacent chromatin regions. Publicly available ChIP-seq tracks were included to annotate the transcription factor occupancy at this region. Blue and orange bars depict the annotated left and right chromatin looping anchors identified by Hi-C. **C** Screenshot of the *BLCAP* gene and surrounding chromatin regions with ATAC-seq tracks of CTCF^AID^ cells with and without IAA treatment. Averaged tracks are combined analyses of three individual clones—RNA-seq tracks of CTCF^AID^ cells with and without IAA treatment. Publicly available CTCF ChIP-seq tracks were included to indicate CTCF occupancy in this region. WGBS tracks from clone 27 serve as a negative control because no methylation occurred in this region

Although techniques such as Hi-C, Hi-ChIP, and chromatin interaction analysis by paired-end tag sequencing permit genome-wide characterization of CTCF-mediated insulators, systematic and functional annotation of such CTCF-bound insulators in noncoding regions of the genome remains challenging. We set up a framework to identify such putative insulator elements by integrative analysis. By overlapping the 3490 ATAC-seq peaks colocalized with adjacent CTCF-binding peaks containing conserved CTCF motifs, we found 716 increased ATAC-seq peaks (fold change > 2, FDR-corrected *p* value < 0.05). We next matched them to the upregulated genes in our RNA-seq results (fold change > 2, FDR-corrected *p* value < 0.05) if the TSSs were located 2 to 50 kb away from the DARs. In summary, 67 genes passed these criteria (Fig. 4A). We found that 20 of these 67 genes were supported by a nearby chromatin loop called from the Hi-C data (Additional file 5: Table S4). For example, a putative repressive CTCF-binding peak was observed ~ 7 kb upstream of the *BLCAP* gene, which physically resided in a chromatin insulation loop shown by Hi-C (Fig. 4B). In control CTCF^AID^ cells without IAA treatment, CTCF bound to this motif leading to repressed chromatin accessibility evident by the absence of ATAC-seq signals. However, upon acute CTCF loss, both ATAC-seq peak signal and *BLCAP* mRNA expression were notably increased (Fig. 4C).

### Functional validation of the role of CTCF on repressing *BLCAP* expression

**Fig. 5:**
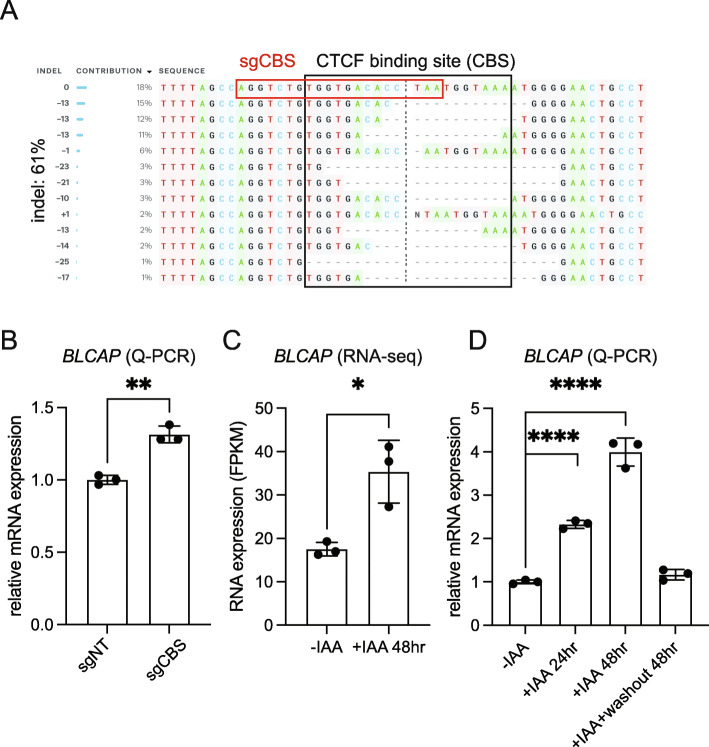
Functional validation of the role of CTCF on repressing BLCAP expression. **A** Cas9-expressing SEM cells infected with sgRNA against CBS were collected for genomic PCR and Sanger sequencing. Indel frequency was evaluated by Inference of CRISPR Edits (ICE). **B** Q-PCR was conducted in sgBLCAP-CBS targeted bulk population and sgRNA-NT control using primers against BLCAP mRNA. **C** Quantification of mRNA change of BLCAP upon CTCF loss by RNA-seq. **D** Q-PCR validation was conducted to examine the mRNA expression of BLCAP in response to acute depletion of CTCF protein by auxin treatment for 24 and 48 h and washout. *p value < 0.05; **p value < 0.01; ****p value < 0.0001. Student’s *t* test

To further validate the role of predicted putative insulators, we conducted the following experiments to investigate the regulation. First, we designed a guide RNA targeting the CTCF binding site (CBS) within the CTCF binding peak in the distal non-coding region upstream of the *BLCAP* promoter. Lentiviral-expressing guide RNA was infected into Cas9-expressing SEM cells followed by antibiotic selection. Sanger genomic sequencing (Inference of CRISPR Edits, ICE) detected about 61% overall indel frequency in the targeted pool population (Fig. 5A), which led to a significant increase of *BLCAP* mRNA expression compared with a non-targeting guide control (sgNT) (Fig. 5B). Alternatively, since there is only one CTCF binding peak in the distal non-coding region upstream of the *BLCAP* promoter, we believe acute depletion of CTCF protein should provide a complementary result to support the functional regulation of the CTCF/BLCAP axis. We treated CTCF^AID^ cells with IAA for 24 and 48 h and then washout out the IAA for CTCF restoration. RNA-seq analysis and Q-PCR validation were conducted to examine the mRNA expression of *BLCAP* in response to acute depletion of CTCF protein (Fig. 5C). As expected, the *BLCAP* expression level significantly increased upon acute depletion of CTCF protein by auxin treatment for 24 or 48 h. More importantly, the expression level was restored to the level seen in parental cells after auxin was washed out (Fig. 5D). In summary, these data strongly support that the CTCF occupancy at the regulatory region of *BLCAP* serves as a functional insulator controlling *BLCAP* expression. To further confirm the transcriptional regulation of repressive effects in other loci, we conducted Q-PCR with primers specific for additional candidate genes that showed increased chromatin accessibility at CTCF binding motifs following CTCF acute loss. We treated CTCF^AID^ cells with IAA for 24 and 48 h and then washed out the IAA for CTCF rescue. We observed consistent induction of randomly selected candidate genes, including *TMEM173*, *MRXA7*, *STAT3*, and *STAT5A* upon CTCF degradation. When the CTCF protein was recovered in response to the IAA washout, transcription was completely restored to the parental control cells’ levels (Additional file 1: Fig. S11)**.** These data suggest that our combined analysis is robust in identifying novel repressive CTCF-binding sites.

### Multi-omics integration reveals CTCF co-regulatory partners

**Fig. 6:**
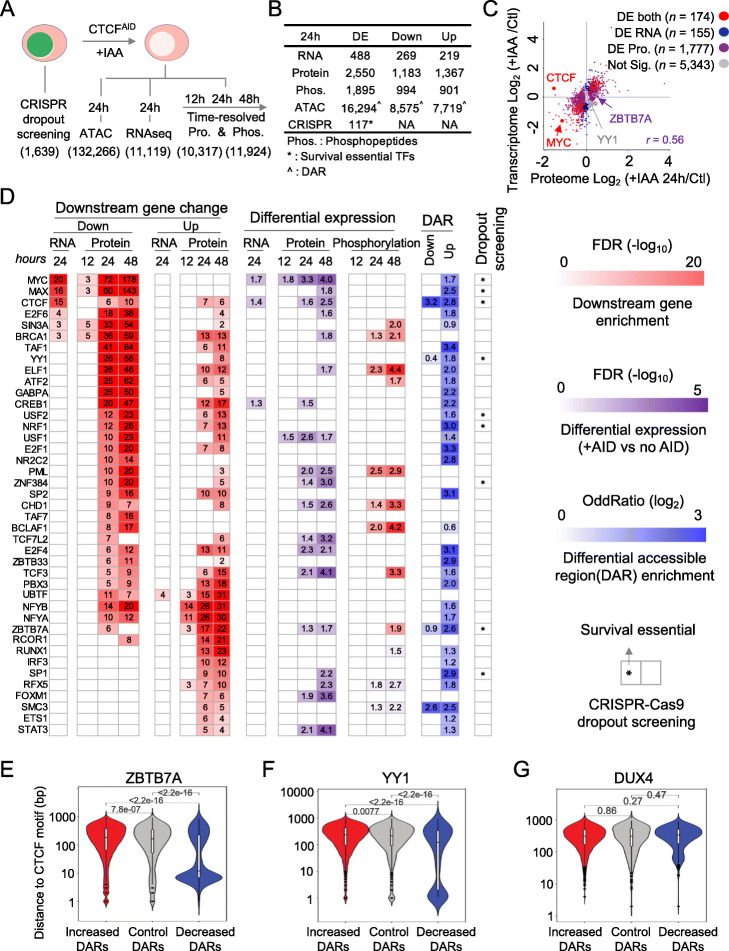
Multi-omics integration reveals main CTCF co-regulatory partners in regulating downstream gene expression. **A** Scheme of multi-omics analyses including CRISPR dropout screening, ATAC-seq, RNA-seq, proteomics, and phosphoproteomics with total 1639 TFs, 132,266 DARs, 11,119 mRNAs, 10,317 proteins, and 11,924 phosphopeptides respectively. **B** Summary of DE analysis for each dataset. **C** More DE proteins were identified compared to DE mRNAs upon CTCF acute loss. The scatterplot shows the log_2_ fold change of proteins (*x*-axis) and log_2_ fold change of mRNAs (*y*-axis) upon CTCF acute loss at 24 h. Pearson correlation coefficient and representative gene names are labeled. **D** CTCF co-regulatory TFs revealed by multi-omics integration. Heatmaps indicate (1) the enrichment (FDR) of downstream DE genes of CTCF partner TFs at RNA and protein levels showing either upregulation or downregulation (red). (2) FDR values of the DE analyses comparing the expression of these TFs in acute CTCF loss groups to the control group in the transcriptome, proteome, and phosphoproteome data (purple). (3) Enrichment (Log_2_ OddRatio) of TFs at decreased DAR and increased DAR (blue). (4) Survival essential genes identified in the CRISPR-Cas9 dropout screening in SEM cells (asterisk). **E** The physical distance between CTCF and ZBTB7A binding motifs was calculated within increased, decreased, and control DARs, normalized to peak size. **F** The physical distance between CTCF and YY1 binding motifs was calculated within increased, decreased, and control DARs, normalized to peak size. **G** The physical distance between CTCF and negative control DUX4 binding motifs was calculated within increased, decreased, and control DARs, which were normalized to peak size. Student’s *t* test

To further investigate the impact of acute CTCF loss on gene expression, we systemically explored CTCF-mediated downstream gene expression at proteome and phosphoproteome levels and integrated them with ATAC-seq and RNA-seq (Fig. 6A). We also included data collected from a dropout CRISPR/Cas9 screen to unbiasedly reveal the survival dependency genes in SEM cells by targeting 1639 transcription factors (Additional file 1: Fig. S12 and Additional file 6: Table S5) [45]. Time-resolved deep proteomic analyses of CTCF^AID^ treatments at 12, 24, and 48 h compared to control cells were carried out via an advanced TMT-LC/LC-MS/MS platform (Additional file 1: Fig. S13A) [46–51], which resulted in high-quality and deep proteomic and phosphoproteomic data (Additional file 1: Fig. S13B-E). In total, we identified 2550 differentially expressed proteins (1183 down and 1367 up) and 1895 differentially expressed phosphopeptides (994 down and 901 up) with FDR < 0.05 out of total 10,317 and 11,924 quantified proteins and phosphopeptides, respectively, by comparing 24 h treatment to no IAA treatment group (Fig. 6B and Additional file 1: Fig. S13F) (Additional file 7: Table S6). While we observed a reasonable correlation (*r* = 0.56) between global proteome and transcriptome (Fig. 6C), there were only 488 DE mRNAs (269 down and 219 up) that passed a cutoff of FDR < 0.05 (Additional file 8: Table S7). These data suggest that although the mRNA level changes were not robust and acute CTCF loss induced substantial downstream disruption, which was observed in differential protein expression and phosphorylation.

Consistent with the immunoblotting and Q-PCR results, mass-spectrum (MS)-based proteomics and RNA-seq analyses confirmed the substantial loss of CTCF expression at the protein level and increased expression at the mRNA level following acute loss of CTCF. While the loss of CTCF is lethal in SEM cells [19], the molecular network has not been systematically studied. Thus, to explore the main transcription programs that CTCF mediates and utilizes to rewire the downstream molecular network changes, we developed a multi-omics integrative approach to define CTCF co-regulatory transcription factors. We first required that CTCF co-regulatory partners significantly enrich a deregulated downstream gene either upregulated or downregulated at RNA and/or protein levels upon CTCF loss. We further limited these TFs to ones that have evidence of protein level expression supported by MS detection. Identified TFs were further prioritized by their mRNA, protein, and/or phosphorylation changes upon CTCF loss. In total, we identified 40 CTCF co-regulatory partner TFs that have significantly affected their downstream target genes’ expression at the mRNA and/or protein level upon acute CTCF loss (Fig. 6D). We further categorized these partners into two categories. (1) The evident master co-regulatory partners have expression alterations either at RNA, protein, or phosphorylation levels, including MYC, E2F4, TCF3, and STAT3. For instance, MYC, a well-characterized CTCF co-regulatory partner [19], showed a profound decrease at the mRNA and protein levels following CTCF loss, and its downstream genes were also the most significantly downregulated after IAA treatment (enrichment FDR < 1 × 10^–72^ at 24 h, and FDR < 1 × 10^−178^ at 48 h). Moreover, MYC’s binding sites were significantly enriched in CTCF-mediated DARs upon acute CTCF loss highlighting it can work synergistically with CTCF; (2) hidden master partners that do not show an evident difference of their expression following acute CTCF depletion but exhibit substantial proteomic changes to their respective downstream gene targets (e.g., YY1, TAF1, USF2, NFYB, NFYA, NRF1, E2F1, and SP1). As expected, most of these 40 CTCF co-regulatory partners also co-localize within CTCF-mediated DARs, confirming their potential direct co-regulatory role with CTCF. Furthermore, we investigated the co-regulation pattern of CTCF and selected candidate TFs ZBTB7A and YY1. We hypothesized that if acute depletion of CTCF impaired chromatin accessibility and adjacent TF binding, we should be able to detect the proximity of both binding motifs within a subset of loci. To this end, the overall distance between the CTCF binding motif and ZBTB7A or YY1 motifs was calculated among increased, decreased, and control DARs identified previously. The data demonstrated that the physical distance between pairwise CTCF/ZBTB7A and CTCF/YY1 motifs are notably closer (less than 100 bp, *p* < 2.2 × 10^–16^) in decreased DARs compared with the others. As a negative control, this pattern was not observed between CTCF and DUX motifs (*p* = 0.47). Notably, the list of CTCF co-regulatory TFs was significantly enriched in survival essential genes (i.e., MYC, MAX, YY1, USF2, NRF1, ZNF384, ZBTB7A, SP1) when compared to the complete list of TFs in the CRISPR-Cas9 library (1639 TFs) dropout screening (Fisher exact test *p* = 0.0237) [45], underlining their indispensable roles in CTCF-mediated regulation of downstream molecular networks for supporting fundamental cellular and molecular functions. To further confirm the potential co-regulatory function between CTCF and the hidden TFs identified by the multi-omics study above, within each category of DARs, the physical distance between CTCF/ZBTB7A and CTCF/YY1 was calculated. The data suggest that the vast majority of the two motifs are closer in decreased DARs than control and increased DARs, suggesting that CTCF loss might affect adjacent open chromatin accessibility leading to the loss of binding other TFs (Fig. 6E, F). As a negative control, DUX4 and CTCF motif distance is equally distributed (Fig. 6G). In summary, we systematically unveiled and validated the leading co-regulatory partner TFs that CTCF mediates and recruits to fulfill downstream transcription regulation through the weaving and altering of chromatin accessibility and demonstrated that our multi-omics pipeline is robust to identify hidden master regulators that do not show expression changes.

## Discussion

Despite recent advancements in understanding CTCF biology, several aspects remain unresolved because of a lack of adequate research tools. *CTCF* haploinsufficiency destabilizes DNA methylation [17], and CTCF can interact with TET enzymes, thereby promoting DNA methylation of adipogenic transcriptional enhancers during adipocyte cell differentiation [52]. *CTCF* knockdown in prostate cancer cells leads to hypermethylation at CTCF-binding sites [40]. In contrast, CTCF binding is reported to be DNA methylation dependent [17, 38, 53], which is further supported by structural analysis [54]. Whether one of these two regulatory functions is more applicable genome-wide than the other is unknown. Chromatin accessibility is generally negatively associated with DNA methylation levels [55, 56]. Upon *DNMT1* and *DNMT3B* double knockout in HCT116 cells, the DMRs exhibited increased chromatin accessibility and were enriched for CTCF motifs [57]. This is in line with a report that decreased DARs in p63 mutant keratinocytes are enriched for CTCF motifs and thereby decreased CTCF binding [58], further suggesting that CTCF regulates chromatin accessibility. However, another report suggested that BATF is a pioneer factor that can regulate chromatin accessibility. However, it is unknown whether BATF can directly bind to DNA and recruit CTCF [59]. Indeed, it is still unclear whether CTCF directly regulates chromatin accessibility or if chromatin accessibility controls CTCF binding before transcription. Regardless of which mechanism they supported, most of these studies rely heavily on crude knockout of CTCF, leading to mixed secondary effects on transcription during the long-term expansion of CTCF-depleted cells.

Using a state-of-the-art acute CTCF degradation system and rich available datasets, we provide direct evidence that CTCF regulates chromatin accessibility but not DNA methylation. These data successfully fill a knowledge gap by uncoupling the direct effects of CTCF from its downstream effects. Our results shed light on the mechanism by which CTCF exerts numerous molecular functions. To our knowledge, this is the first report that shows sites with decreased DARs upon CTCF loss exhibit a tandem CTCF-binding pattern that is associated with CTCF motif orientation. These data also support the hypothesis that transcriptional regulation most likely occurs where CTCF and CTCFL/BORIS reside together [36]. Because CTCF and CTCFL do not appear to recruit each other [60], CTCF may maintain chromatin accessibility at tandem CTCF-binding sites, thereby recruiting CTCFL to nearby genes and initiating transcription. In contrast, most gene promoters were highly depleted of nucleosomes, resulting from combinatorial TF binding. In these sites, loss of CTCF binding will not completely turn off transcription. Interestingly, a recent study led by Owens et al. also revealed that CTCF confers local nucleosome resiliency after DNA replication and during mitosis [28], which can lend support to the potential role of CTCF in changing chromatin accessibility and binding of other GTFs.

We also found that the distance between tandem CTCF sites previously reported for 2xCTSes (~ 33 bp) [19] was shorter than those within decreased DARs (~ 200 bp). This accounts for the augmented number of 2xCTSes for increased DARs and our findings of double-dip footprint profiles. For sites with few TF binding, CTCF may create a barrier for only limited nucleosome occupancy, thus allowing TF access to chromatin. In contrast, for a site with many putative TFs or Pol II binding, such as promoters, CTCF cannot compete with multiple TFs. Together, our data suggest a simplified yet effective strategy for how a highly conserved TF, such as the zinc finger–containing CTCF, can exhibit diverse regulatory functions by fine-tuning the distance of tandem motifs. This accelerates our understanding of the function of CTCF and other dimers/tetramer factors, such as STAT5A [61]. Moreover, the established framework and multi-omics datasets may be broadly useful for the research community.

While acute deletion of CTCF can profoundly disturb global chromatin interactions and accessibilities, transcription is often not significantly altered [19, 22]. We extended our molecular profiling beyond the transcriptome to characterize the proteome and phosphoproteome changes. Recent advancements in MS-based technologies have allowed in-depth analyses of protein products of almost all confidently expressed transcripts [48, 62–66], providing unprecedented opportunities to systematically characterize molecular phenotypes contributed by transcription, translation, and post-translational modifications via multi-omics integrative analysis [46, 47, 49, 51, 67–69]. Through an advanced TMT-LC/LC-MS/MS proteomic protocol [48, 63–66], we quantitatively analyzed > 10,000 proteins and around 12,000 phosphosites across all samples and discovered 2550 DE proteins with FDR < 0.05 upon CTCF loss for 24 h, a more than 5-fold change compared to the number of DE mRNAs. In addition, we also identified 1895 protein phosphorylation changes with FDR < 0.05. Together, these findings indicate potential global changes occurred during protein translation and post-translational modification upon CTCF acute loss. It is possible that acute CTCF loss disrupted major components in translation, therefore derailing the protein translation machinery. Indeed, we found that ribosome biogenesis-related terms are the most significantly altered GO annotation via GSEA analyses of the transcriptome and proteome upon CTCF loss (data not shown). A limitation of the study is that the bulk mRNA sequencing may suffer from limitations of differential mRNA stabilities and turnover. Advanced nascent transcript sequencing methods, e.g., NET-seq, should be able to address this issue. In summary, acute deletion of CTCF changed chromatin interactions and accessibilities globally. Although transcription changes were not dramatic, we observed strong protein expression and post-translational modification changes. Further studies are required to understand better how the loss of CTCF induces big changes in proteins and post-translational modifications.

We and others demonstrated that long-term loss of CTCF induced massive cell death [19], highlighting its indispensable role in maintaining cell integrity. The present study further unveiled that CTCF fulfilled its functions primarily through altering chromatin interactions and accessibilities upon acute loss; however, downstream genes of CTCF were barely altered, indicating that its role as a transcription factor was minor. We hypothesize that the global changes of chromatin accessibility and interaction triggered by acute CTCF loss will cause relocation, switching, and genome comprehensive occupancy reprogramming of other master transcription factors, further altering these TF’s downstream gene expression. Therefore, it is critical to identify these CTCF co-regulatory partners. We identified 40 CTCF co-regulatory partner TFs with significant reprogramming of downstream targets at the mRNA and/or protein level upon acute CTCF loss through multi-omics integrative approaches. Notably, most of these 40 TFs showed enrichment of motifs in the DARs upon CTCF loss, and their functional importance was further validated by our CRISPR-Cas9 dropout screening highlighting significant enrichment of essential survival genes in these 40 partner TFs in SEM. We expected the co-regulatory partners could cooperate with CTCF in multiple ways. For instance, direct binding, which likely occurs in the decreased DARs, indirect interaction, and occupancy switching, preferably happens in the increased DARs; these different mechanisms present in a loci-dependent manner, and multiple mechanisms can occur for the same TFs in distinct loci. We observed enrichment of downstream genes in both upregulated and downregulated pools for many of the 40 detected co-regulatory partners, supporting the possibility of a distinct mechanism of cooperation with CTCF for the same TFs at different loci. Most importantly, our data reveals CTCF’s ability to affect co-regulators’ binding affinity to target genes, which was under-appreciated in previous studies. However, functional validation in the future is required to confirm these observations.

We also acknowledge that residual CTCF protein can be detected upon auxin treatment in CTCF^AID^ knockin clones. For instance, although CTCF protein seems to completely disappear when monitored by immunoblotting, flow cytometry and CUT&RUN still can detect some positive signals. There are many possible explanations to address this observation. Dr. Gerd Blobel’s group recently reported that a minimal amount of chromatin-bound CTCF is retained upon auxin treatment [70]. Also, random integration of different copies of OsTir1 may lead to a variable expression level of OsTir1. Finally, clonal variation of genetically engineered CTCF^AID^ lines may also contribute to incomplete protein degradation. To mitigate this challenge, a more powerful acute depletion system is required, which is currently not available to our knowledge.

In brief, here we unveiled many CTCF co-regulatory partners through systematic integration of ATAC-seq, RNA-seq, Hi-C, Cut&Run, CRISPR-Cas9 TF library dropout screening, and MS-based deep proteomic and phosphoproteomic data, largely broadening our understanding of the transcriptional network mediated by CTCF. The mechanism and functional consequence behind the interactions between CTCF and these partners is worth further investigation into other systems. Finally, the rich next-generation sequencing data collected from the acute CTCF degradation model will be valuable resources for the research community.

## Materials and methods

### Cell culture

The human B-ALL cell line SEM (DSMZ) carrying bi-allelic miniAID-mClover3 knockin tags was established in a previous study [19]. Three single-cell derived clones (clones 27, 35, and 42) were maintained in RPMI-1640 medium (Lonza) containing 10% fetal bovine serum (Hyclone), 2 mM glutamine (Sigma), and 1% penicillin/streptomycin (Thermo Fisher Scientific). USF1/USF2 double-knockout SEM cells were created by co-targeting cells with two validated guide RNAs described in our previous study (sgUSF1: 5′- CTATACTTACTTCCCCAGCA-3′; sgUSF2: 5′AGAAGAGCCCAGCACAACGA3′)^44^. The BCLAP-CBS targeted pool population was generated by delivering guide RNA (5′- AGGTCTGTGGTGACACCTAA-3′) into the Cas9-expressing SEM cell line. All cells were negative for mycoplasma infection, and their identity was confirmed by short tandem repeat analysis.

### Immunoblotting

Cell lysates were prepared in RIPA buffer, subjected to SDS-PAGE (Thermo Fisher Scientific), and transferred to PVDF membranes (Bio-Rad) at 100 V for 1 h. After blocking, the membranes were incubated with 5% nonfat milk in TBS-T (10 mM Tris, pH 8.0, 150 mM NaCl, 0.5% Tween-20) containing antibodies against GAPDH (Thermo Fisher Scientific, AM4300, 1:10,000), USF1 (Proteintech, 22327–1-AP, 1:2000), USF2 (Novus, NBP1-92649, 1:2000), and CTCF (Abcam, ab70303, 1:1000) at 4 °C for 12 h with gentle shaking. The membranes were washed three times for 30 min and incubated with a 1:2000 dilution of horseradish peroxidase-conjugated anti-mouse or anti-rabbit antibodies for 2 h at room temperature. The blots were washed with TBS-T three times for 30 min and developed with the ECL system (Amersham Biosciences).

### Auxin-induced degradation

Three single-cell derived knockin clones (clones 27, 35, and 42) were treated with complete medium supplemented with 500 μM IAA (Sigma) for 24 h or 48 h to induce CTCF degradation. IAA washout was performed by centrifuging and resuspending the cells in PBS three times and then maintaining in culture for 48 h in a regular medium without IAA.

### Quantitative real-time PCR

Reverse transcription was performed by using the High-Capacity cDNA Reverse Transcriptase kit (Applied Biosystems, 4374966). The real-time Q-PCR was performed using FAST SYBR Green Master Mix (Applied Biosystems, 4385612), with specific primers to amplify *CTCF, BLCAP, MEM173*, *MXRA7*, *STAT3*, *STAT5A,* and *GAPDH*. Relative gene expression was determined by using the ^ΔΔ^CT method [71].

### ATAC-seq protocol and data analysis

Briefly, 75,000 cells were collected per sample in duplicate and resuspended in cold PBS with protease inhibitors. After centrifugation at 500 rpm for 5 min at 4 °C (Eppendorf 5417R refrigerated centrifuge), cell pellets were resuspended in cold lysis buffer with protease inhibitors (10 mM Tris pH 7.4, 10 mM NaCl, 3 mM MgCl_2_, and 0.1% IGEPAL), followed by centrifugation. The pellets were resuspended in 25 μL Tagment DNA Buffer (Nextera, FC-121-1030) and then used directly in the transposition reaction. Nextera Tn5 (Nextera, FC-121-1030) was added to the resuspended nuclei, and the transposition reaction was incubated at 37 °C for 30 min. After transposition, the DNA was purified using a Qiagen MinElute PCR purification kit (Qiagen, 28004). Indexing PCR was carried out for 12 cycles with NEBNext HiFi 2X PCR Master Mix (NEB, M0541S) and indexing primers, as previously described [72]. PCR products were purified using a 1:3 ratio of Agencourt AMPure XP beads (Beckman Coulter, A63881), and the samples were sequenced at 100-bp paired-ends with an Illumina HiSeq 4000 system. For computational analysis, the reads were trimmed for the Nextera adapter by cutadapt (v1.9, paired-end mode, default parameter with “–m 6 –O 20”) and aligned to the human genome hg19 (GRCh37-lite) by BWA (v0.7.12-r1039, default parameter) [73]. The duplicated reads were then marked with biobambam2 (v2.0.87), and only non-duplicated proper paired reads were kept by samtools (parameter “–q 1 –F 1804,” v1.2) [74]. After removing the mitochondrial DNA reads, the rest of the reads were classified into four groups: nucleosome-free and mono-, di-, and tri-nucleosomes by fragment size. The bigwig files were generated by using the center 80-bp fragments and scaled to 20 million nucleosome-free reads. We observed reasonable nucleosome-free peaks and patterns of mono-, di-, and tri-nucleosome peaks surrounding the nucleosome-free peaks on the Integrative Genomics Viewer (Broad Institute). All samples exhibited double the ENCODE criteria. Therefore, we concluded that the data showed enough depth. Given that all samples exhibited more than 15 million nucleosome-free fragments, we were confident that most of the strong NFRs were not missed. MACS2 conducted peak calling on the nucleosome-free reads (v2.1.1.20160309, default parameters with “--extsize 200 –nomodel”) [75]. To assure data reproducibility, we finalized the peaks for each group as only a retained peak if it was called with a stringent cutoff (macs2 –q 0.05) in one merged sample and was at least called with a lower cutoff (macs2 –q 0.5) in the other merged sample. The reproducible peaks were further merged between the groups to create a final set of reference chromatin accessible regions. We then counted the nucleosome-free reads from each sample overlapping the reference regions by bedtools (v2.24.0) [76]. The reproducibility was optimal because the Spearman correlation coefficient between the replicates was > 0.9 and larger than the between-sample variability from different groups. To elucidate the DARs, we normalized the raw nucleosome-free read counts used to trim the mean of the *M*-value normalization method and applied empirical Bayes statistical tests after linear fitting from the voom package (R 3.23, edgeR 3.12.1, limma 3.26.9) [77]. DARs were extracted using an FDR-corrected *p* value < 0.05 (Benjamini–Hochberg procedure) and fold change > 2. Cutoff *p* value > 0.5 and fold change < 1.05 were used for control NFRs (no change). To detect TF-enriched DARs, we scanned the TRANSFAC motif database [33] by using FIMO (parameter “--motif-pseudo 0.0001 --thresh 1e-4”) from the MEME suite (v4.11.3) [78]. For each motif, we counted how many DARs or control regions had motif matches and used Fisher exact tests to estimate their enrichment over the background (DAR or control regions without motif matches). For the top motifs enriched, we also performed footprint profiling with deeptools2 (v2.5.7) [79]. Quality control analysis and motif analysis of ATAC-seq peak were provided in Additional file 3: Table S2.

### Whole-genome bisulfite sequencing and analysis

Genomic DNA collected from Clone 27 with or without IAA treatment for 24 h was extracted with the PureLink Genomic DNA Mini kit (Thermo Fisher Scientific). Approximately 2 μg of genomic DNA was used for bisulfite DNA conversion and subsequent cleanup of the converted DNA with the EpiTect Fast Bisulfite Conversion kit (Qiagen), followed by library construction and next-generation sequencing. The FASTQ sequencing files were first trimmed by removing adapter sequences with trimgalore and mapped to the human genome (hg19) by BSMAP (v2.74, parameters “–m 17 –x 600 –u -R –z 33 –f 5 –g 3 –r 0 –p 6”) [80]. CpGs were then extracted by the methratio.py in the BSMAP package. We then confirmed optimal depth (each sample > 900 million reads), coverage (> 95% CpG had more than five reads and > 90% CpG had more than 10 reads), and C > T conversion rate (> 90%). DMRs were identified using the Bioconductor package DSS [81] and custom R scripts, with a threshold of > 0 change in methylation ratio and *p* value ≤ 0.01 as a cutoff. The minimum length for DMRs was 50 bp, and the minimum number of CpG sites for DMR was 3. DMR information was provided in Additional file 4: Table S3.

### Data analysis of a dropout CRISPR screen

A dropout CRISPR screen was done in SEM cells to target 1639 human transcription factors with seven sgRNAs designed against each gene [45]. The Cas9-expressing SEM cells infected with the pooled library of sgRNAs (M.O.I = ~ 0.3) were collected at day 0 and day 12 to sequence for differentially represented sgRNA at the late time. The rationale of this screen is based on the fact that read counts of sgRNAs against essential survival genes will be depleted on day 12 compared with day 0. Following the instruction of MAGeCK analysis, there are 117 TFs identified as essential survival genes based on the cutoff of MAGeCK score less than 0.01. The complete gene list can be found in Additional file 7, Table S6.

### Deep profiling of the whole proteome and phosphoproteome analyses

Two million CTCF^AID^ SEM cells from 4 treatment groups: no IAA, + IAA 12 h, + IAA 24 h, and + IAA 48 h, each with triplicates, were applied for deep proteomic and phosphoproteomic analyses using a well-established protocol [48, 65, 66]. In brief, cells were lysed in fresh lysis buffer (50 mM HEPES, pH 8.5, 8 M urea, 1× PhosStop Phosphatase inhibitor, 0.5% sodium deoxycholate). Proteins were quantified by the BCA protein assay (Thermo Fisher Scientific). About 100 μg proteins from each sample were digested with Lys-C (Wako, 1:100 w/w) for 2 h, followed by trypsin digestion (Promega, 1:50 w/w) overnight at room temperature after 4× dilution with 50 mM HEPES buffer. The resulting peptides from each sample were desalted, labeled with TMTpro reagents, and equally pooled. TiO_2_-based phosphoproteomic enrichment was then performed on the pooled sample, and flow-through was further desalted and applied for off-line basic-PH fractionation. Peptides were separated into 80 fractions via a 2 h gradient, and every other fraction was dried and reconstituted in 5% formic acid for MS analysis. Peptides were analyzed on an Orbitrap HF mass spectrometer (Thermo Fisher Scientific) after separated on a 20 cm × 75 μm id column packed with 1.9 μm C18 resin (Dr. Maisch GmbH, Germany) and heated at 55 °C. Peptide separation was achieved through a 2 h ~ 15–40% buffer B (0.2% formic acid, 65% CAN, 3% DMSO) gradient. The mass spectrometer was set in DDA mode with 60,000 resolution, 1 × 10^6^ AGC target, and 50 ms maximal ion time for MS1, Top 10, 1 × 10^5^ AGC target, 105 ms maximal ion time, 1 m/z isolation window and 0.2 m/z offset, 38 NCE, and 15 s dynamic exclusion for MS2. Proteomic data were processed by the hybrid JUMP software suites for improving sensitivity and specificity [63, 64]. Briefly, raw files were searched against the Uniprot human database, and the same search and filtering parameters were applied to achieve 1% protein or phosphopeptide FDR using the target-decoy approach [82]. Detailed expression data were provided in Additional file 7: Table S6.

### Statistical analysis

Statistical analyses for Q-PCR data were calculated with a two-tailed *t*-test from two or three independent biological replicates with GraphPad Prism 6.0. Fisher exact tests and Wilcoxon tests were performed with R. The figures were plotted with R (ggplot2, ggpubr or ggally), deeptools, or Excel.

## Supplementary Information

**Additional file 1: Supplementary Figures-Figures S1-S13.**

**Additional file 2: Supplementary Tables-Tables S1.** ATAC-seq peak data.

**Additional file 3: Supplementary Tables-Tables S2.** ATAC-seq statistics and QC.

**Additional file 4: Supplementary Tables-Tables S3.** DMR profiles.

**Additional file 5: Supplementary Tables-Tables S4.** Insulator targets.

**Additional file 6: Supplementary Table-Tables S5.** MAGeCK analysis of CRISPR screen.

**Additional file 7: Supplementary Tables -Tables S6.** Differential expression analysis of whole proteome and phosphoproteome data.

**Additional file 8: Supplementary Tables -Tables S7.** Summary of RNA-seq data.

**Additional file 9.**

## Data Availability

All plasmids created in this study will be deposited to Addgene. Raw data collected from ATAC-seq and WGBS were deposited at NCBI GEO (GSE153237[83]). The publicly available datasets used in this study are cited accordingly (GSE120781[84], GSE126619[85], GSE138862[86]). Raw proteomic data supporting this study's findings are deposited at ProteomeXchange (Accession: PXD026484[87]).

## References

[1] Phillips JE, Corces VG (2009). CTCF: master weaver of the genome. Cell.

[2] Filippova GN, Fagerlie S, Klenova EM, Myers C, Dehner Y, Goodwin G, Neiman PE, Collins SJ, Lobanenkov VV (1996). An exceptionally conserved transcriptional repressor, CTCF, employs different combinations of zinc fingers to bind diverged promoter sequences of avian and mammalian c-myc oncogenes. Mol Cell Biol.

[3] Singh P, Lee D-H, Szabó PE. More than insulator: multiple roles of CTCF at the H19-Igf2 imprinted domain. Front Genet. 2012;3. 10.3389/fgene.2012.00214. 10.3389/fgene.2012.00214 PMC3471092 23087708

[4] Splinter E, Heath H, Kooren J, Palstra R-J, Klous P, Grosveld F, Galjart N (2006). Laat Wd: CTCF mediates long-range chromatin looping and local histone modification in the β-globin locus. Genes Dev.

[5] Ong C-T, Corces VG (2014). CTCF: an architectural protein bridging genome topology and function. Nat Rev Genet.

[6] Pombo A, Dillon N (2015). Three-dimensional genome architecture: players and mechanisms. Nat Rev Mol Cell Biol.

[7] de Wit E, Vos Erica SM, Holwerda Sjoerd JB, Valdes-Quezada C, Verstegen Marjon JAM, Teunissen H, Splinter E, Wijchers Patrick J, Krijger Peter HL, de Laat W (2015). CTCF binding polarity determines chromatin looping. Mol Cell.

[8] Guo Y, Xu Q, Canzio D, Shou J, Li J, Gorkin David U, Jung I, Wu H, Zhai Y, Tang Y (2015). CRISPR inversion of CTCF sites alters genome topology and enhancer/promoter function. Cell.

[9] Rao SSP, Huntley MH, Durand NC, Stamenova EK, Bochkov ID, Robinson JT, Sanborn AL, Machol I, Omer AD, Lander ES, Aiden EL (2014). A 3D map of the human genome at kilobase resolution reveals principles of chromatin looping. Cell.

[10] Fudenberg G, Imakaev M, Lu C, Goloborodko A, Abdennur N, Mirny Leonid A (2016). Formation of chromosomal domains by loop extrusion. Cell Rep.

[11] Gassler J, Brandão HB, Imakaev M, Flyamer IM, Ladstätter S, Bickmore WA, Peters J-M, Mirny LA, Tachibana K (2017). A mechanism of cohesin-dependent loop extrusion organizes zygotic genome architecture. EMBO J.

[12] Nuebler J, Fudenberg G, Imakaev M, Abdennur N, Mirny LA (2018). Chromatin organization by an interplay of loop extrusion and compartmental segregation. Proc Natl Acad Sci.

[13] Rowley MJ, Corces VG (2018). Organizational principles of 3D genome architecture. Nat Rev Genet.

[14] Hsieh T-HS, Cattoglio C, Slobodyanyuk E, Hansen AS, Rando OJ, Tjian R, Darzacq X: Resolving the 3D landscape of transcription-linked mammalian chromatin folding. *Molecular Cell* 2020, 78:539-553.e538. 10.1016/j.molcel.2020.03.002 PMC7703524 32213323

[15] Li Y, Haarhuis JHI, Sedeño Cacciatore Á, Oldenkamp R, van Ruiten MS, Willems L, Teunissen H, Muir KW, de Wit E, Rowland BD, Panne D (2020). The structural basis for cohesin–CTCF-anchored loops. Nature.

[16] Pugacheva EM, Kubo N, Loukinov D, Tajmul M, Kang S, Kovalchuk AL, Strunnikov AV, Zentner GE, Ren B, Lobanenkov VV (2020). CTCF mediates chromatin looping via N-terminal domain-dependent cohesin retention. Proc Natl Acad Sci.

[17] Kemp CJ, Moore JM, Moser R, Bernard B, Teater M, Smith LE, Rabaia NA, Gurley KE, Guinney J, Busch SE, Shaknovich R, Lobanenkov VV, Liggitt D, Shmulevich I, Melnick A, Filippova GN (2014). CTCF haploinsufficiency destabilizes DNA methylation and predisposes to cancer. Cell Rep.

[18] Chen X, Ke Y, Wu K, Zhao H, Sun Y, Gao L, Liu Z, Zhang J, Tao W, Hou Z, Liu H, Liu J, Chen ZJ (2019). Key role for CTCF in establishing chromatin structure in human embryos. Nature.

[19] Hyle J, Zhang Y, Wright S, Xu B, Shao Y, Easton J, Tian L, Feng R, Xu P, Li C (2019). Acute depletion of CTCF directly affects MYC regulation through loss of enhancer-promoter looping. Nucleic Acids Res.

[20] Nora EP, Caccianini L, Fudenberg G, et al. Molecular basis of CTCF binding polarity in genome folding. Nat Commun. 2020;11(5612). 10.1038/s41467-020-19283-x. 10.1038/s41467-020-19283-x PMC7645679 33154377

[21] Splinter E, Heath H, Kooren J, Palstra RJ, Klous P, Grosveld F, Galjart N, de Laat W (2006). CTCF mediates long-range chromatin looping and local histone modification in the beta-globin locus. Genes Dev.

[22] Nora EP, Goloborodko A, Valton A-L, Gibcus JH, Uebersohn A, Abdennur N, Dekker J, Mirny LA, Bruneau BG: Targeted degradation of CTCF decouples local insulation of chromosome domains from genomic compartmentalization. Cell 2017, 169:930-944.e922. 10.1016/j.cell.2017.05.004 PMC5538188 28525758

[23] Heath H, de Almeida CR, Sleutels F, Dingjan G, van de Nobelen S, Jonkers I, Ling K-W, Gribnau J, Renkawitz R, Grosveld F, Hendriks RW, Galjart N (2008). CTCF regulates cell cycle progression of αβ T cells in the thymus. EMBO J.

[24] Ren L, Wang Y, Shi M, Wang X, Yang Z, Zhao Z (2012). CTCF mediates the cell-type specific spatial organization of the Kcnq5 locus and the local gene regulation. PLoS One.

[25] Moore JM, Rabaia NA, Smith LE, Fagerlie S, Gurley K, Loukinov D, Disteche CM, Collins SJ, Kemp CJ, Lobanenkov VV, Filippova GN (2012). Loss of maternal CTCF is associated with peri-implantation lethality of Ctcf null embryos. PLoS One.

[26] Hirayama T, Tarusawa E, Yoshimura Y, Galjart N, Yagi T (2012). CTCF is required for neural development and stochastic expression of clustered Pcdh genes in neurons. Cell Rep.

[27] Degner SC, Verma-Gaur J, Wong TP, Bossen C, Iverson GM, Torkamani A, Vettermann C, Lin YC, Ju Z, Schulz D, Murre CS, Birshtein BK, Schork NJ, Schlissel MS, Riblet R, Murre C, Feeney AJ (2011). CCCTC-binding factor (CTCF) and cohesin influence the genomic architecture of the Igh locus and antisense transcription in pro-B cells. Proc Natl Acad Sci U S A.

[28] Owens N, Papadopoulou T, Festuccia N, Tachtsidi A, Gonzalez I, Dubois A, Vandormael-Pournin S, Nora EP, Bruneau BG, Cohen-Tannoudji M, Navarro P (2019). CTCF confers local nucleosome resiliency after DNA replication and during mitosis. eLife.

[29] Nishimura K, Fukagawa T, Takisawa H, Kakimoto T, Kanemaki M (2009). An auxin-based degron system for the rapid depletion of proteins in nonplant cells. Nat Methods.

[30] Holland AJ, Fachinetti D, Han JS, Cleveland DW (2012). Inducible, reversible system for the rapid and complete degradation of proteins in mammalian cells. Proc Natl Acad Sci U S A.

[31] Heinz S, Benner C, Spann N, Bertolino E, Lin YC, Laslo P, Cheng JX, Murre C, Singh H, Glass CK (2010). Simple combinations of lineage-determining transcription factors prime cis-regulatory elements required for macrophage and B cell identities. Mol Cell.

[32] Kuleshov MV, Jones MR, Rouillard AD, Fernandez NF, Duan Q, Wang Z, Koplev S, Jenkins SL, Jagodnik KM, Lachmann A, McDermott MG, Monteiro CD, Gundersen GW, Ma'ayan A (2016). Enrichr: a comprehensive gene set enrichment analysis web server 2016 update. Nucleic Acids Res.

[33] Matys V, Kel-Margoulis O, Fricke E, Liebich I, Land S, Barre-Dirrie A, Reuter I, Chekmenev D, Krull M, Hornischer K, Voss N, Stegmaier P, Lewicki-Potapov B, Saxel H, Kel AE, Wingender E (2006). TRANSFAC and its module TRANSCompel: transcriptional gene regulation in eukaryotes. Nucleic Acids Res.

[34] Ernst J, Kheradpour P, Mikkelsen TS, Shoresh N, Ward LD, Epstein CB, Zhang X, Wang L, Issner R, Coyne M, Ku M, Durham T, Kellis M, Bernstein BE (2011). Mapping and analysis of chromatin state dynamics in nine human cell types. Nature.

[35] Ernst J, Kellis M (2012). ChromHMM: automating chromatin state discovery and characterization. Nat Methods.

[36] Pugacheva EM, Rivero-Hinojosa S, Espinoza CA, Méndez-Catalá CF, Kang S, Suzuki T, Kosaka-Suzuki N, Robinson S, Nagarajan V, Ye Z, Boukaba A, Rasko JEJ, Strunnikov AV, Loukinov D, Ren B, Lobanenkov VV (2015). Comparative analyses of CTCF and BORIS occupancies uncover two distinct classes of CTCF binding genomic regions. Genome Biol.

[37] Knight PA, Ruiz D (2013). A fast algorithm for matrix balancing. IMA J Numerical Analysis.

[38] Wang H, Maurano MT, Qu H, Varley KE, Gertz J, Pauli F, Lee K, Canfield T, Weaver M, Sandstrom R, Thurman RE, Kaul R, Myers RM, Stamatoyannopoulos JA (2012). Widespread plasticity in CTCF occupancy linked to DNA methylation. Genome Res.

[39] Ishihara K, Oshimura M, Nakao M (2006). CTCF-dependent chromatin insulator is linked to epigenetic remodeling. Mol Cell.

[40] Damaschke NA, Gawdzik J, Avilla M, Yang B, Svaren J, Roopra A, Luo J-H, Yu YP, Keles S, Jarrard DF (2020). CTCF loss mediates unique DNA hypermethylation landscapes in human cancers. Clin Epigenetics.

[41] Schuijers J, Manteiga JC, Weintraub AS, Day DS, Zamudio AV, Hnisz D, Lee TI, Young RA (2018). Transcriptional dysregulation of MYC reveals common enhancer-docking mechanism. Cell Rep.

[42] Elbert A, Vogt D, Watson A, Levy M, Jiang Y, Brûlé E, Rowland ME, Rubenstein J, Bérubé NG (2019). CTCF governs the identity and migration of MGE-derived cortical interneurons. J Neurosci.

[43] Saldaña-Meyer R, González-Buendía E, Guerrero G, Narendra V, Bonasio R, Recillas-Targa F, Reinberg D (2014). CTCF regulates the human p53 gene through direct interaction with its natural antisense transcript, Wrap53. Genes Dev.

[44] Liberzon A, Subramanian A, Pinchback R, Thorvaldsdóttir H, Tamayo P, Mesirov JP (2011). Molecular signatures database (MSigDB) 3.0. Bioinformatics.

[45] Zhang H, Zhang Y, Zhou X, Wright S, Hyle J, Zhao L, An J, Zhao X, Shao Y, Xu B, Lee HM, Chen T, Zhou Y, Chen X, Lu R, Li C (2020). Functional interrogation of HOXA9 regulome in MLLr leukemia via reporter-based CRISPR/Cas9 screen. eLife.

[46] Bai B, Wang X, Li Y, Chen P-C, Yu K, Dey KK, Yarbro JM, Han X, Lutz BM, Rao S, et al: Deep multilayer brain proteomics identifies molecular networks in Alzheimer’s disease progression. Neuron 2020, 105:975-991.e977. 10.1016/j.neuron.2019.12.015 PMC7318843 31926610

[47] Dey KK, Wang H, Niu M, Bai B, Wang X, Li Y, Cho J-H, Tan H, Mishra A, High AA, Chen PC, Wu Z, Beach TG, Peng J (2019). Deep undepleted human serum proteome profiling toward biomarker discovery for Alzheimer’s disease. Clin Proteomics.

[48] Niu M, Cho J-H, Kodali K, Pagala V, High AA, Wang H, Wu Z, Li Y, Bi W, Zhang H, Wang X, Zou W, Peng J (2017). Extensive peptide fractionation and y1 ion-based interference detection method for enabling accurate quantification by isobaric labeling and mass spectrometry. Anal Chem.

[49] Stewart E, McEvoy J, Wang H, Chen X, Honnell V, Ocarz M, Gordon B, Dapper J, Blankenship K, Yang Y, et al: Identification of therapeutic targets in rhabdomyosarcoma through integrated genomic, epigenomic, and proteomic analyses. Cancer Cell 2018, 34:411-426.e419. 10.1016/j.ccell.2018.07.012 PMC6158019 30146332

[50] Wang H, Dey KK, Chen P-C, Li Y, Niu M, Cho J-H, Wang X, Bai B, Jiao Y, Chepyala SR, Haroutunian V, Zhang B, Beach TG, Peng J (2020). Integrated analysis of ultra-deep proteomes in cortex, cerebrospinal fluid and serum reveals a mitochondrial signature in Alzheimer’s disease. Mol Neurodegeneration.

[51] Wang H, Diaz AK, Shaw TI, Li Y, Niu M, Cho J-H, Paugh BS, Zhang Y, Sifford J, Bai B, Wu Z, Tan H, Zhou S, Hover LD, Tillman HS, Shirinifard A, Thiagarajan S, Sablauer A, Pagala V, High AA, Wang X, Li C, Baker SJ, Peng J (2019). Deep multiomics profiling of brain tumors identifies signaling networks downstream of cancer driver genes. Nat Commun.

[52] Dubois-Chevalier J, Oger F, Dehondt H, Firmin FF, Gheeraert C, Staels B, Lefebvre P, Eeckhoute J (2014). A dynamic CTCF chromatin binding landscape promotes DNA hydroxymethylation and transcriptional induction of adipocyte differentiation. Nucleic Acids Res.

[53] Shukla S, Kavak E, Gregory M, Imashimizu M, Shutinoski B, Kashlev M, Oberdoerffer P, Sandberg R, Oberdoerffer S (2011). CTCF-promoted RNA polymerase II pausing links DNA methylation to splicing. Nature.

[54] Hashimoto H, Wang D, Horton JR, Zhang X, Corces VG, Cheng X: Structural basis for the versatile and methylation-dependent binding of CTCF to DNA. *Molecular Cell* 2017, 66:711-720.e713. 10.1016/j.molcel.2017.05.004 PMC5542067 28529057

[55] Clark SJ, Argelaguet R, Kapourani C-A, Stubbs TM, Lee HJ, Alda-Catalinas C, Krueger F, Sanguinetti G, Kelsey G (2018). Marioni JC, et al: scNMT-seq enables joint profiling of chromatin accessibility DNA methylation and transcription in single cells. Nat Commun.

[56] Thurman RE, Rynes E, Humbert R, Vierstra J, Maurano MT, Haugen E, Sheffield NC, Stergachis AB, Wang H, Vernot B, Garg K, John S, Sandstrom R, Bates D, Boatman L, Canfield TK, Diegel M, Dunn D, Ebersol AK, Frum T, Giste E, Johnson AK, Johnson EM, Kutyavin T, Lajoie B, Lee BK, Lee K, London D, Lotakis D, Neph S, Neri F, Nguyen ED, Qu H, Reynolds AP, Roach V, Safi A, Sanchez ME, Sanyal A, Shafer A, Simon JM, Song L, Vong S, Weaver M, Yan Y, Zhang Z, Zhang Z, Lenhard B, Tewari M, Dorschner MO, Hansen RS, Navas PA, Stamatoyannopoulos G, Iyer VR, Lieb JD, Sunyaev SR, Akey JM, Sabo PJ, Kaul R, Furey TS, Dekker J, Crawford GE, Stamatoyannopoulos JA (2012). The accessible chromatin landscape of the human genome. Nature.

[57] Spektor R, Tippens ND, Mimoso CA (2019). Soloway PD: methyl-ATAC-seq measures DNA methylation at accessible chromatin. Genome Res.

[58] Qu J, Yi G, Zhou H (2019). p63 cooperates with CTCF to modulate chromatin architecture in skin keratinocytes. Epigenetics Chromatin.

[59] Pham D, Moseley CE, Gao M, Savic D, Winstead CJ, Sun M, Kee BL, Myers RM, Weaver CT, Hatton RD: Batf pioneers the reorganization of chromatin in developing effector T cells via Ets1-dependent recruitment of Ctcf. Cell Reports 2019, 29:1203-1220.e1207. 10.1016/j.celrep.2019.09.064 PMC7182170 31665634

[60] Nishana M, Ha C, Rodriguez-Hernaez J, Ranjbaran A, Chio E, Nora EP, Badri SB, Kloetgen A, Bruneau BG, Tsirigos A, Skok JA (2020). Defining the relative and combined contribution of CTCF and CTCFL to genomic regulation. Genome Biol.

[61] Lin J-X, Li P, Liu D, Jin Hyun T, He J (2012). Rasheed Mohammed Ata U, Rochman Y, Wang L, Cui K, Liu C, et al: Critical role of STAT5 transcription factor tetramerization for cytokine responses and normal immune function. Immunity.

[62] Kim M-S, Pinto SM, Getnet D, Nirujogi RS, Manda SS, Chaerkady R, Madugundu AK, Kelkar DS, Isserlin R, Jain S, Thomas JK, Muthusamy B, Leal-Rojas P, Kumar P, Sahasrabuddhe NA, Balakrishnan L, Advani J, George B, Renuse S, Selvan LDN, Patil AH, Nanjappa V, Radhakrishnan A, Prasad S, Subbannayya T, Raju R, Kumar M, Sreenivasamurthy SK, Marimuthu A, Sathe GJ, Chavan S, Datta KK, Subbannayya Y, Sahu A, Yelamanchi SD, Jayaram S, Rajagopalan P, Sharma J, Murthy KR, Syed N, Goel R, Khan AA, Ahmad S, Dey G, Mudgal K, Chatterjee A, Huang TC, Zhong J, Wu X, Shaw PG, Freed D, Zahari MS, Mukherjee KK, Shankar S, Mahadevan A, Lam H, Mitchell CJ, Shankar SK, Satishchandra P, Schroeder JT, Sirdeshmukh R, Maitra A, Leach SD, Drake CG, Halushka MK, Prasad TSK, Hruban RH, Kerr CL, Bader GD, Iacobuzio-Donahue CA, Gowda H, Pandey A (2014). A draft map of the human proteome. Nature.

[63] Wang X, Li Y, Wu Z, Wang H, Tan H, Peng J (2014). JUMP: A Tag-based database search tool for peptide identification with high sensitivity and accuracy. Mol Cell Proteomics.

[64] Li Y, Wang X, Cho J-H, Shaw TI, Wu Z, Bai B, Wang H, Zhou S, Beach TG, Wu G, Zhang J, Peng J (2016). JUMPg: an integrative proteogenomics pipeline identifying unannotated proteins in human brain and cancer cells. J Proteome Res.

[65] Tan H, Wu Z, Wang H, Bai B, Li Y, Wang X, Zhai B, Beach TG, Peng J (2015). Refined phosphopeptide enrichment by phosphate additive and the analysis of human brain phosphoproteome. Proteomics.

[66] Wang H, Yang Y, Li Y, Bai B, Wang X, Tan H, Liu T, Beach TG, Peng J, Wu Z (2015). Systematic optimization of long gradient chromatography mass spectrometry for deep analysis of brain proteome. J Proteome Res.

[67] Zhang B, Wang J, Wang X, Zhu J, Liu Q, Shi Z, Chambers MC, Zimmerman LJ, Shaddox KF, Kim S (2014). Proteogenomic characterization of human colon and rectal cancer. Nature.

[68] Gillette MA, Satpathy S, Cao S, Dhanasekaran SM, Vasaikar SV, Krug K, Petralia F, Li Y, Liang WW, Reva B, et al: Proteogenomic characterization reveals therapeutic vulnerabilities in lung adenocarcinoma. Cell 2020, 182:200-225.e235. 10.1016/j.cell.2020.06.013 PMC7373300 32649874

[69] Chang T-C, Carter RA, Li Y, Li Y, Wang H, Edmonson MN, Chen X, Arnold P, Geiger TL, Wu G, Peng J, Dyer M, Downing JR, Green DR, Thomas PG, Zhang J (2017). The neoepitope landscape in pediatric cancers. Genome Med.

[70] Zhang H, Emerson DJ, Gilgenast TG, Titus KR, Lan Y, Huang P, Zhang D, Wang H, Keller CA, Giardine B, Hardison RC, Phillips-Cremins JE, Blobel GA (2019). Chromatin structure dynamics during the mitosis-to-G1 phase transition. Nature.

[71] Schmittgen TD, Livak KJ (2008). Analyzing real-time PCR data by the comparative C(T) method. Nat Protoc.

[72] Buenrostro JD, Giresi PG, Zaba LC, Chang HY, Greenleaf WJ (2013). Transposition of native chromatin for fast and sensitive epigenomic profiling of open chromatin, DNA-binding proteins and nucleosome position. Nat Methods.

[73] Li H, Durbin R: Fast and accurate short read alignment with Burrows-Wheeler transform. Bioinformatics (Oxford, England) 2009, 25:1754-1760. 10.1093/bioinformatics/btp324 PMC2705234 19451168

[74] Li H, Handsaker B, Wysoker A, Fennell T, Ruan J, Homer N, Marth G, Abecasis G, Durbin R, Genome Project Data Processing S: The Sequence Alignment/Map format and SAMtools. Bioinformatics (Oxford, England) 2009, 25:2078-2079. 10.1093/bioinformatics/btp352 PMC2723002 19505943

[75] Zhang Y, Liu T, Meyer CA, Eeckhoute J, Johnson DS, Bernstein BE, Nusbaum C, Myers RM, Brown M, Li W, Liu XS (2008). Model-based analysis of ChIP-Seq (MACS). Genome Biol.

[76] Quinlan AR, Hall IM: BEDTools: a flexible suite of utilities for comparing genomic features. Bioinformatics (Oxford, England) 2010, 26:841-842. 10.1093/bioinformatics/btq033 PMC2832824 20110278

[77] Law CW, Chen Y, Shi W (2014). Smyth GK: voom: precision weights unlock linear model analysis tools for RNA-seq read counts. Genome Biol.

[78] Bailey TL, Boden M, Buske FA, Frith M, Grant CE, Clementi L, Ren J, Li WW, Noble WS (2009). MEME SUITE: tools for motif discovery and searching. Nucleic Acids Res.

[79] Ramírez F, Ryan DP, Grüning B, Bhardwaj V, Kilpert F, Richter AS, Heyne S, Dündar F (2016). Manke T: deepTools2: a next generation web server for deep-sequencing data analysis. Nucleic Acids Res.

[80] Xi Y, Li W (2009). BSMAP: whole genome bisulfite sequence MAPping program. BMC Bioinformatics.

[81] Wu H, Xu T, Feng H, Chen L, Li B, Yao B, Qin Z, Jin P, Conneely KN (2015). Detection of differentially methylated regions from whole-genome bisulfite sequencing data without replicates. Nucleic Acids Res.

[82] Peng J, Elias JE, Thoreen CC, Licklider LJ, Gygi SP (2003). Evaluation of multidimensional chromatography coupled with tandem mass spectrometry (LC/LC − MS/MS) for large-scale protein analysis: the yeast proteome. J Proteome Res.

[83] Xu B, Wright S, Hyle J, Zhang Y, Shao Y, Fan Y, Lu R, Li C. Acute depletion of CTCF rewires genome-wide chromatin accessibility. GSE153237. Gene Expression Omnibus. 2021. https://www.ncbi.nlm.nih.gov/geo/query/acc.cgi?acc = GSE153237. Accessed 25 June 2020. 10.1186/s13059-021-02466-0 PMC8386078 34429148

[84] Hyle J, Zhang Y, Wright S, Li C. Acute depletion of CTCF directly affects MYC regulation through loss of enhancer–promoter looping. GSE120781. Gene Expression Omnibus. 2019. https://www.ncbi.nlm.nih.gov/geo/query/acc.cgi?acc = GSE120781. Accessed 4 May 2019. 10.1093/nar/gkz462 PMC6648894 31127282

[85] Zhang Y, Hyle J, Wright S, Li C. Acute depletion of CTCF directly affects MYC regulation through loss of enhancer–promoter looping. GSE126619. Gene Expression Omnibus. 2019. https://www.ncbi.nlm.nih.gov/geo/query/acc.cgi?acc = GSE126619. Accessed 4 May 2019. 10.1093/nar/gkz462 PMC6648894 31127282

[86] Hyle J, Zhang Y, Wright S, Li C. Acute depletion of CTCF directly affects MYC regulation through loss of enhancer–promoter looping. GSE138862. Gene Expression Omnibus. 2019. https://www.ncbi.nlm.nih.gov/geo/query/acc.cgi?acc = GSE138862. Accessed 16 Oct 2019. 10.1093/nar/gkz462 PMC6648894 31127282

[87] Wang H, Niu M, Peng J, Li C. Acute depletion of CTCF rewires genome-wide chromatin accessibility. PXD026484. ProteomeXchange PRoteomics IDEntifications (PRIDE) Database. 2021.http://www.ebi.ac.uk/pride/archive/projects/PXD026484. Accessed 7 Apr 2021. 10.1186/s13059-021-02466-0 PMC8386078 34429148

