# Supplementary material for: Acute depletion of CTCF rewires genome-wide chromatin accessibility
Source: Genome Biol. 2021 Aug 24;22:244. doi: 10.1186/s13059-021-02466-0 (PMC8386078; doi:10.1186/s13059-021-02466-0)
Supplement: Supplementary file 1 — Additional file 1: Supplementary Figures-Figures S1-S13. [file 13059_2021_2466_MOESM1_ESM.pdf]

Figure Legends related to Additional file 1 (Fig. S1-S13)

Additional file, Fig. S1 A Validation of CTCF depletion by Dox/IAA treatment for 24 and 48 hours treatment in three clones. Wild-type SEM cells were included as a control. **B** Validation of USF1/2 knockdown by immunoblotting. Two sgRNAs against USF1 and USF2 were delivered by lentivirus into Cas9-expressing SEM cells, followed by antibiotic selection. **C** Spearman correlation analysis was conducted to quantify the total ATAC-seq peaks among each sample with or without IAA treatment.

Additional file, Fig. S2 A Top *de novo* motifs enriched for decreased differential accessibility regions (DARs) were shown by Homer software. **B** Top *de novo* motifs enriched for increased DARs were shown by Homer software. **C** Top gene sets enriched for genes assigned by nearest promoters (transcriptional start site ± 2 kb) to decreased DARs with EnrichR in the ChIP enrichment analysis database. **D** Top gene sets enriched for genes assigned by nearest promoters (transcriptional start site ± 2 kb) to increased DARs.

Additional file, Fig. S3 Kmeans clustering (deeptools) was used to check whether there is a small set of regions showing the double-summit pattern in **A** control nucleosome-free regions, **B** increased differential accessibility regions (DARs), or **C** decreased DARs.

Additional file, Fig. S4 A Profiles of four clusters from deeptools Kmeans analysis for decreased differential accessibility regions (as shown in Supplementary Fig. 3C) but re-

oriented by the closest CTCF motif strands. **B** Violin plots of the mean height from CTCF Cut&Run tracks comparing regions (200 bp) on the left side of the CTCF motifs to the right side of the CTCF motifs. Replicates were plot separately and analyzed by Wilcoxon tests.

Additional file, Fig. S5 A ATAC-seq footprint profiles depicting additional top motifs enriched and protected by Tn5 insertion in increased differential accessibility regions (DARs) in $CTCF^{AID}$ cells with and without IAA treatment. **B** The same motifs shown in Fig. 2C are depicted in ATAC-seq footprint profiles in increased DARs in $CTCF^{AID}$ cells with and without IAA treatment.

Additional file, Fig. S6 A Schematic diagram showing how genomic regions were defined for annotation of nucleosome-free regions. The GENCODE database (v24lift37) was used for gene definitions. **B** Genomic annotation and proportion for control nucleosome-free regions. **C** Genomic annotation proportion for decreased differential accessibility regions (DARs). **D** Genomic annotation proportion for increased DARs. Note strong enrichment at promoters, primarily upstream of promoters (transcriptional start site ± 2 kb).

Additional file, Fig. S7 A Comparing DARs with enrichment of chromatin states defined in ENCODE K562 data. Note that the insulator states were defined by CTCF binding from ChIP-seq and not necessarily from chromatin states with insulator functions. **B**

Percentage of different groups of nucleosome-free regions annotated by ENCODE K562 chromatin states.

Additional file, Fig. S8 Ggpair plot of normalized Hi-C contact (Knight–Ruiz normalization) for loops overlapping different nucleosome-free region groups. Gray, Blue, Red colors indicated control ATAC peaks, decreased DARs, increased DARs, respectively. **A** Kernel density plots for $CTCF^{AID}$ cells without IAA. **B** Pearson correlation between $CTCF^{AID}$ cells with and without IAA for all, control ATAC-seq peaks, decreased DARs and increased DARs. **C** Boxplot of contacts for $CTCF^{AID}$ cells without IAA. **D** Scatter plot of contact between $CTCF^{AID}$ cells with and without IAA. **E** Kernel density plots for $CTCF^{AID}$ cells with IAA. **F** Boxplot of contacts for $CTCF^{AID}$ cells with IAA. **G** Histogram of contact for $CTCF^{AID}$ cells without IAA. **H** Histogram of contact for $CTCF^{AID}$ cells with IAA. **I** Barplot of the number of loops overlapped with three groups of ATAC-seq peaks, including control ATAC-seq peaks, decreased DARs and increased DARs. **J** Normalized contact number for loops with CTCF binding sites and 2xCTS, loops with CTCF binding sites but not 2xCTS, loops without CTCF binding sites.

Additional file, Fig. S9 Acute CTCF loss does not affect genome-wide DNA methylation. **A** Heatmap of global DNA methylation profiles of increased, decreased, and control ATAC-seq peaks. A 4-kb window flanking each peak center is depicted. **B** Boxplot of the genome-wide DNA methylation profiles in $CTCF^{AID}$ cells with or without IAA treatment. Methylation ratios at the y-axis ranged between 0 and 1 for each CpG. **C** Motif analysis for 49 differentially methylated regions.

Additional file, Fig. S10 A Gene set enrichment analysis (GSEA) profile of the top 200 gene promoters assigned to the increased differential accessibility regions (DARs) upon CTCF depletion in $CTCF^{AID}$ cells treated with and without IAA. Genes were ranked by their log_2 fold change in RNA-seq (i.e., CTCF depletion, +IAA vs. –IAA, left side expression higher with IAA treatment). **B** Further filtering of the top 200 gene promoters assigned to increased DARs by CTCF motifs. **C** GSEA profile of the top 200 gene promoters assigned to decreased DARs. **D** Further filtering of the top 200 gene promoters assigned to decreased DARs by CTCF motifs. **E** Screenshot of the *MYC* promoter with ATAC-seq tracks in $CTCF^{AID}$ cells with and without IAA treatment. **F** Screenshot of the *MYC* distal enhancer resides about 1.8 Mb away with ATAC-seq tracks in $CTCF^{AID}$ cells with and without IAA treatment. Averaged tracks are combined analyses of three individual clones.

Additional file, Fig. S11 A Screenshot of the putative CTCF insulator target *TMEM173*. **B** Screenshot of the putative CTCF insulator targets *MXRA7* and *JMJD6*. **C** Screenshot of the putative CTCF insulator targets *STAT5A* and *STAT3*. **D** Q-PCR validation of TMEM173 expression upon CTCF loss and restoration. **E** Q-PCR validation of MRXA7 expression upon CTCF loss and restoration. **F** Q-PCR validation of STAT3 expression upon CTCF loss and restoration. **G** Q-PCR validation of STAT5A expression upon CTCF loss and restoration. The RNA samples from three $CTCF^{AID}$ clones (27, 35, and 42) were collected for the Q-PCR assay.

Additional file, Fig. S12 Data analysis of dropout CRISPR screens. **A** To incorporate survival dependency genes identified in SEM cells into our multi-omics study, we downloaded raw data from the previous study (Zhang, et al. eLife. 2020 Oct 1;9 e57858) and re-analyzed it by the MAGeCK algorithm. This dropout CRISPR/Cas9 screen was conducted by targeting 1,639 transcription factors with seven sgRNAs designed against each gene. The Cas9-expressing SEM cells infected with the pooled library of sgRNAs (M.O.I=~0.3) were collected at day 0 and day 12 to sequence for sgRNA redundancy. The rationale of this screen is based on the fact that read counts of sgRNAs against essential survival genes will be depleted on day 12 compared with day 0. **B** Following the instruction of MAGeCK analysis, there are 117 TFs identified as essential survival genes based on the cutoff of MAGeCK score less than 0.01. Known survival-essential genes in leukemia cells were identified among this gene list, including PAX5, DOT1L, ZFP64, YY1, MEF2C, KMT2A, and USF2.

Additional file, Fig. S13 Summary of proteomic and phosphoproteomic analyses. **A** design and workflow of time-resolved proteomic and phosphoproteomic analyses. **B** boxplot confirming equal loading of proteins in each sample for proteome analysis. **C** boxplot ensuring equal loading of phosphopeptides in each sample for phosphoproteomic analysis. **D** Principle component analysis of $\log_2$-transformation proteome data. **E** Principle component analysis of $\log_2$-transformation phosphoproteomic data. **F** Summary of DE analyses comparing acute CTCF loss at various time points to control using LIMMA *r* package.

Fig S1

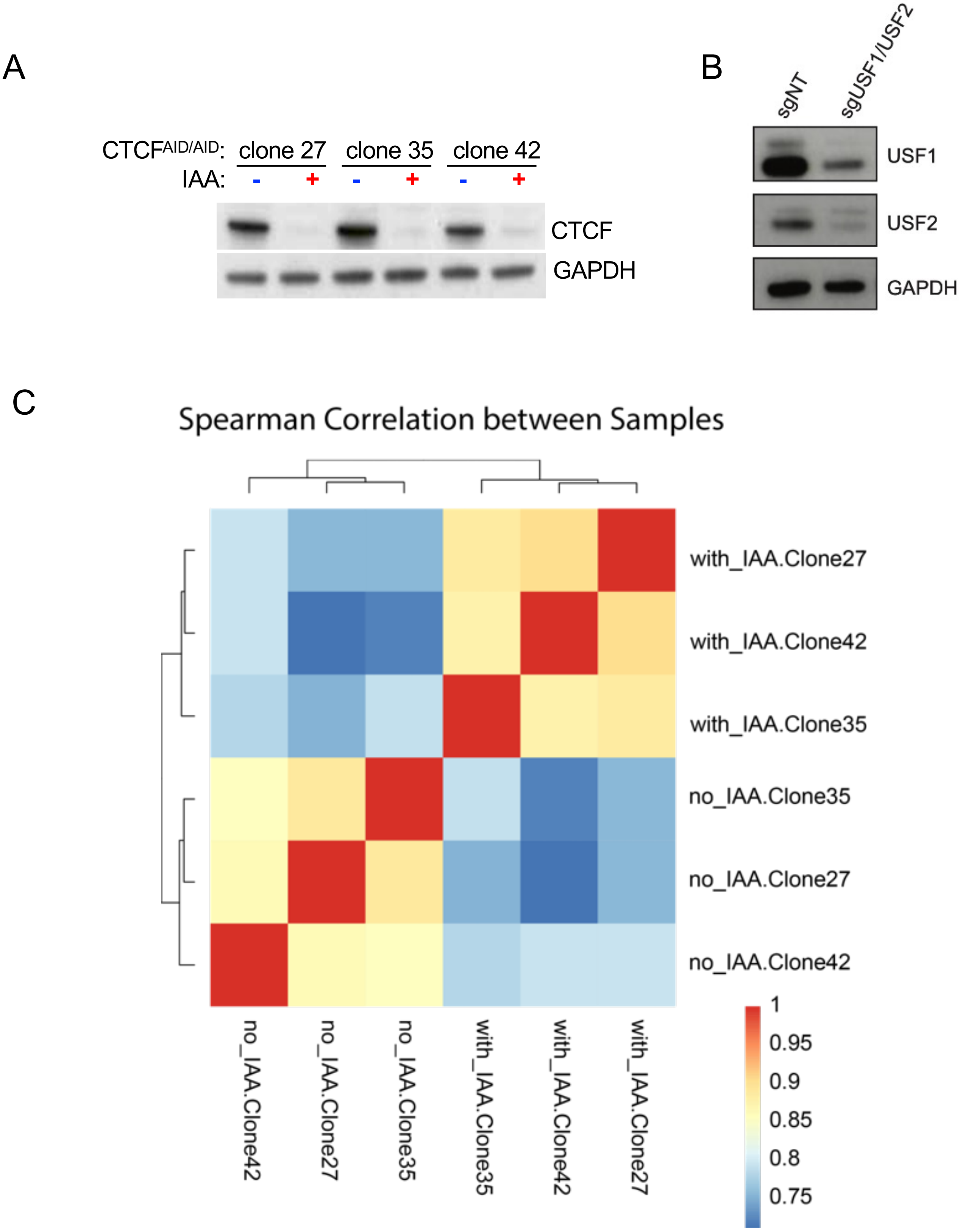
A
CTCF^AID/AID:
clone 27
clone 35
clone 42
IAA:
-
+
-
+
-
+
CTCF
GAPDH
B
sgNT
sgUSF1/USF2
USF1
USF2
GAPDH
C
Spearman Correlation between Samples
with_IAA.Clone27
with_IAA.Clone42
with_IAA.Clone35
no_IAA.Clone35
no_IAA.Clone27
no_IAA.Clone42
no_IAA.Clone42
no_IAA.Clone27
no_IAA.Clone35
with_IAA.Clone35
with_IAA.Clone42
with_IAA.Clone27
1
0.95
0.9
0.85
0.8
0.75

Fig S2

A

De novo motif enrichment analysis in decreased ATAC-seq peaks

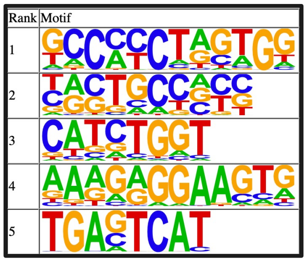

Rank	P-value	% of Targets	Best Match
1	1.00E-4372	46.53%	BORIS/CTCF
2	1.00E-624	13.46%	ZBTB29
3	1.00E-294	57.24%	TCF4
4	1.00E-213	11.48%	SPIB
5	1.00E-131	9.38%	FOS

B

De novo motif enrichment analysis in increased ATAC-seq peaks

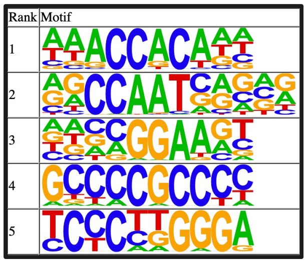

Rank	P-value	% of Targets	Best Match
1	1.00E-1052	32.15%	SFPI1
2	1.00E-803	36.26%	RUNX2
3	1.00E-287	6.60%	IRF8
4	1.00E-145	7.22%	AP1
5	1.00E-78	5.61%	MEF2C

C

ChEA results from Enrichr for genes assigned to decreased differentially accessible regions

Index	Name	P-value	Adjusted p-value	Odds Ratio	Combined score
1	RUNX1 CHEA	1.64E-17	1.71E-15	2.14	82.62
2	IRF3 ENCODE	3.02E-08	0.000001568	2.04	35.32
3	SP1 ENCODE	3.19E-08	0.000001104	2	34.52
4	ZBTB7A ENCODE	4.23E-08	0.000001101	1.51	25.65
5	PBX3 ENCODE	2.09E-07	0.000004337	1.66	25.59
6	FOS ENCODE	0.000001803	0.00002678	1.9	25.1
7	GATA2 CHEA	0.000001766	0.00003062	1.8	23.91
8	E2F1 CHEA	0.00001787	0.0001859	1.67	18.26
9	ATF3 ENCODE	0.001315	0.006837	2.52	16.69
10	SP2 ENCODE	0.00003917	0.0003134	1.59	16.11
11	CTCF ENCODE	0.00001537	0.0001776	1.44	15.99
12	NELFE ENCODE	0.0004969	0.00272	2.1	15.99
13	CREB1 CHEA	0.00003689	0.0003197	1.48	15.06
14	NFYA ENCODE	0.00002542	0.0002403	1.38	14.55
15	KLF4 CHEA	0.00009158	0.0006803	1.56	14.48
16	NFYB ENCODE	0.00001351	0.0001756	1.29	14.41
17	GATA1 CHEA	0.0001595	0.001037	1.6	13.99
18	NRF1 ENCODE	0.00009617	0.0006667	1.38	12.79
19	SMC3 ENCODE	0.0003174	0.001942	1.46	11.74
20	STAT5A ENCODE	0.002422	0.01145	1.93	11.65
21	SIX5 ENCODE	0.0004827	0.002789	1.46	11.16
22	MYC CHEA	0.002058	0.01019	1.57	9.73
23	YY1 CHEA	0.006746	0.02923	1.67	8.33
24	RCOR1 ENCODE	0.004864	0.02199	1.46	7.77
25	NFIC ENCODE	0.01011	0.04043	1.69	7.76
26	BHLHE40 ENCOD	0.01215	0.04679	1.59	7.01
27	TCF3 ENCODE	0.007491	0.03116	1.39	6.81

D

ChEA results from Enrichr for genes assigned to increased differentially accessible regions

Index	Name	P-value	Adjusted p-value	Odds Ratio	Combined score
1	AR CHEA	0.00005693	0.005921	1.86	18.18
2	SRF ENCODE	0.0136	0.3535	2.01	8.65
3	NFE2L2 CHEA	0.004954	0.2576	1.59	8.42
4	SUZ12 CHEA	0.005674	0.1967	1.43	7.39
5	SUZ12 ENCODE	0.07737	1	2.2	5.64
6	RELA ENCODE	0.03479	0.7236	1.63	5.46
7	STAT3 CHEA	0.0899	1	1.83	4.41
8	TRIM28 CHEA	0.08623	1	1.76	4.32
9	TP63 CHEA	0.04253	0.7371	1.32	4.18
10	TRIM28 ENCODE	0.1187	1	1.93	4.11

Fig S3

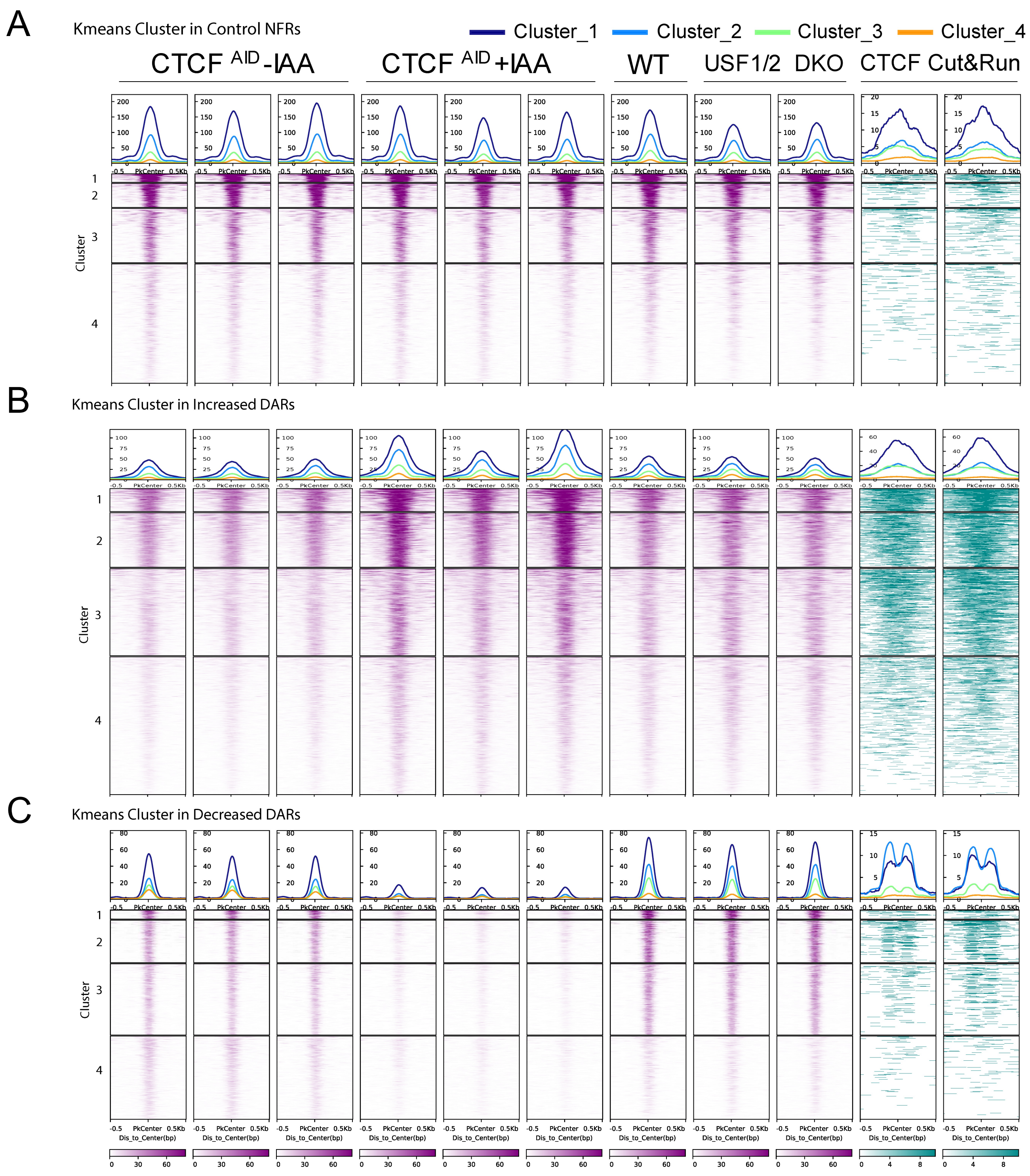

A
Kmeans Cluster in Control NFRs
Cluster_1
Cluster_2
Cluster_3
Cluster_4
CTCF AID -IAA
CTCF AID +IAA
WT
USF1/2 DKO
CTCF Cut&Run
Cluster
B
Kmeans Cluster in Increased DARs
C
Kmeans Cluster in Decreased DARs
-0.5
PkCenter
0.5Kb
Dis_to_Center(bp)

Fig S4

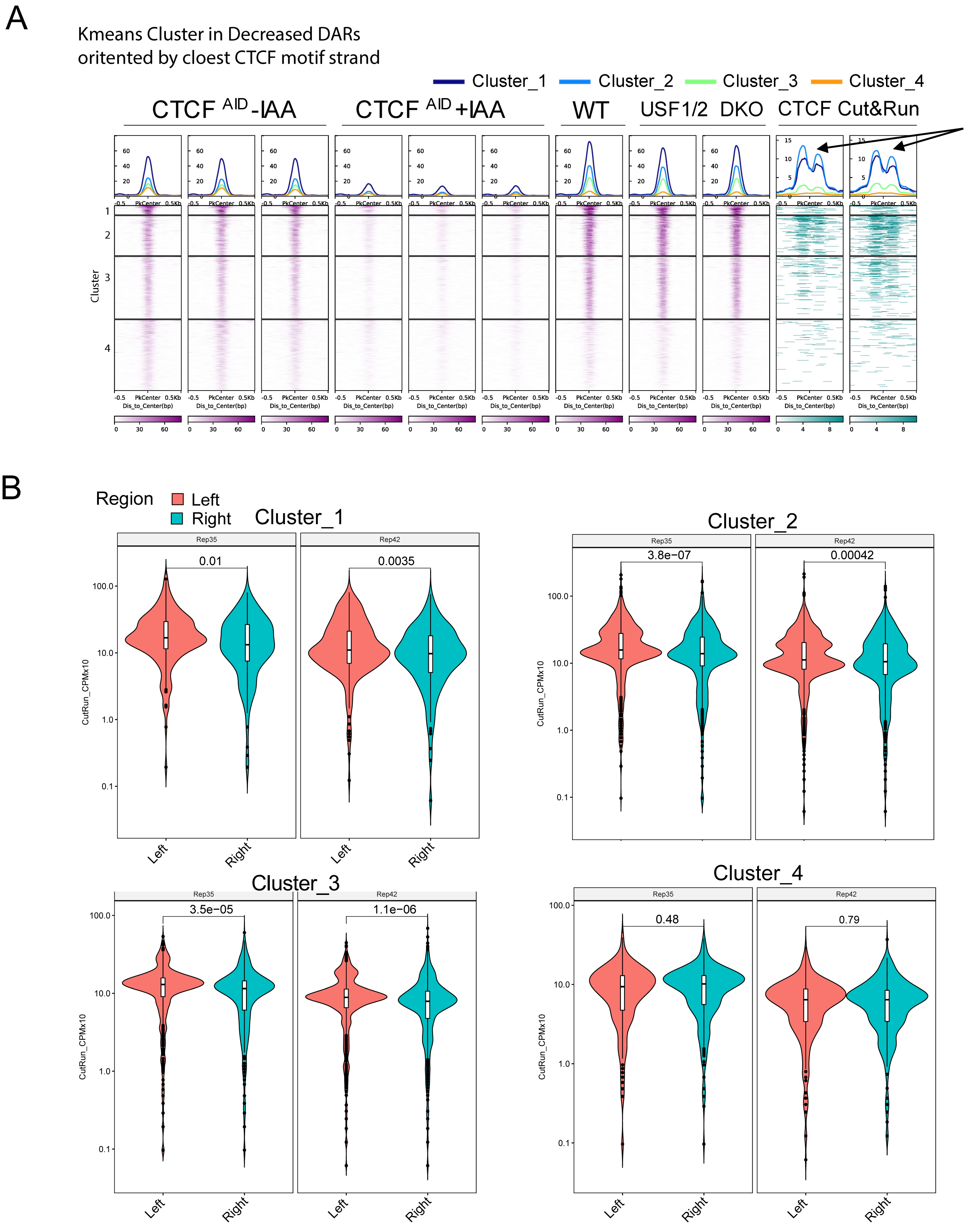

A
Kmeans Cluster in Decreased DARs
oritented by cloest CTCF motif strand
Cluster_1
Cluster_2
Cluster_3
Cluster_4
CTCF AID-IAA
CTCF AID+IAA
WT
USF1/2 DKO
CTCF Cut&Run
Cluster
PkCenter
Dis_to_Center(bp)
B
Region
Left
Right
Cluster_1
Cluster_2
Cluster_3
Cluster_4
Rep35
Rep42
CutRun_CPMx10
0.01
0.0035
3.8e-07
0.00042
3.5e-05
1.1e-06
0.48
0.79

Fig S5

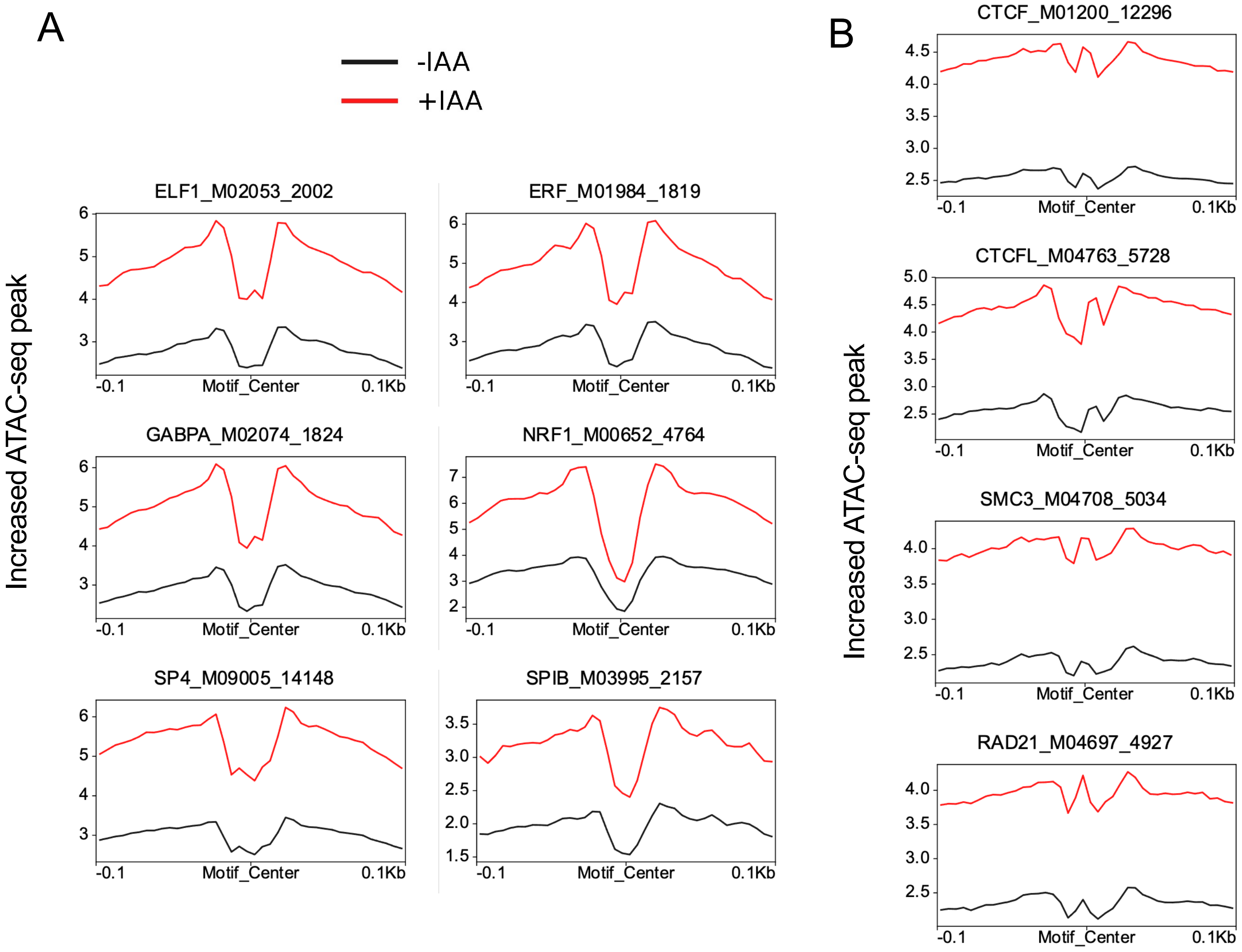
A
-IAA
+IAA
Increased ATAC-seq peak
ELF1_M02053_2002
ERF_M01984_1819
GABPA_M02074_1824
NRF1_M00652_4764
SP4_M09005_14148
SPIB_M03995_2157
-0.1
Motif_Center
0.1Kb
B
CTCF_M01200_12296
CTCFL_M04763_5728
SMC3_M04708_5034
RAD21_M04697_4927

Fig S6

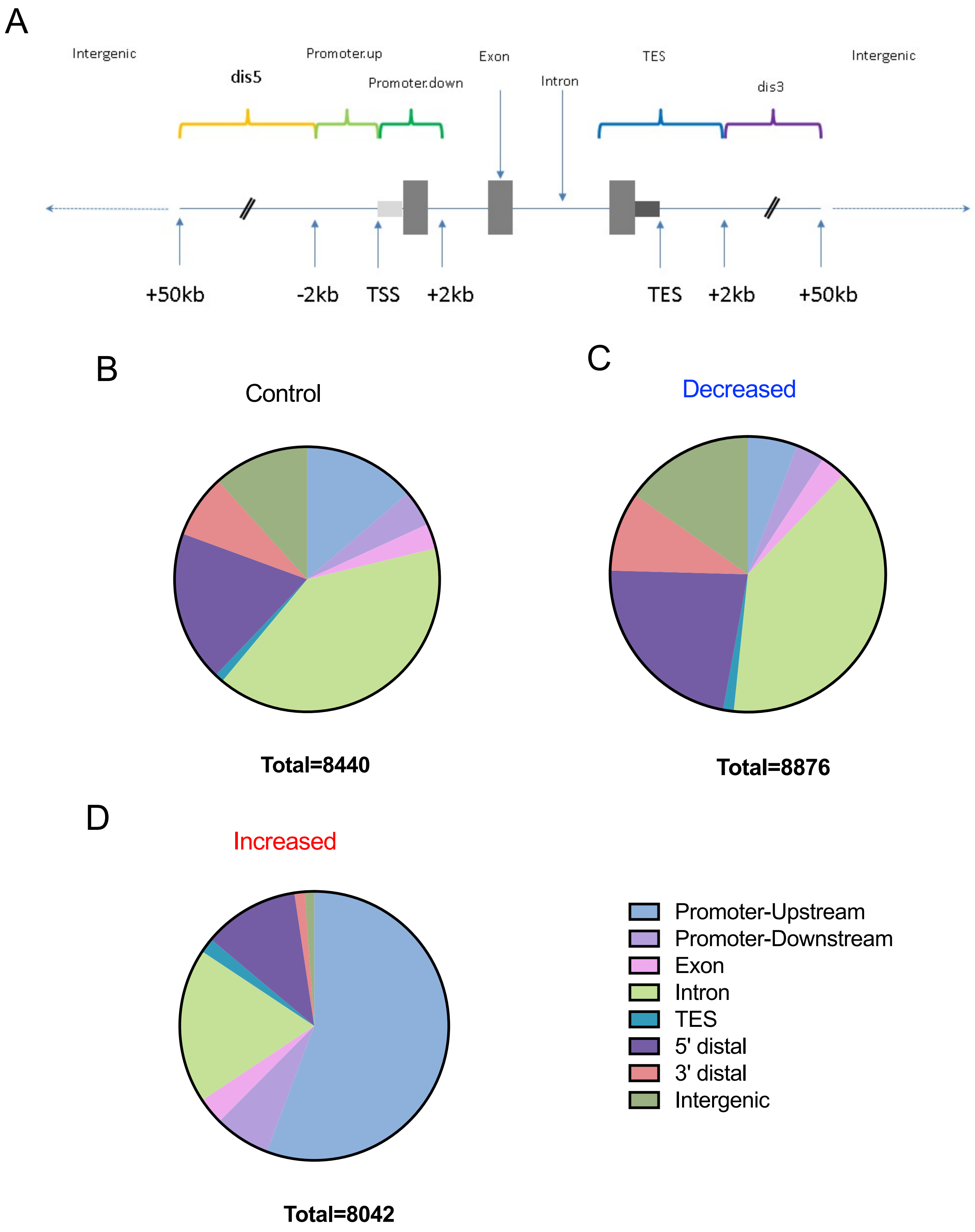
A
Intergenic
dis5
Promoter.up
Promoter.down
Exon
Intron
TES
dis3
Intergenic
+50kb
-2kb
TSS
+2kb
TES
+2kb
+50kb
B
Control
Total=8440
C
Decreased
Total=8876
D
Increased
Total=8042
Promoter-Upstream
Promoter-Downstream
Exon
Intron
TES
5' distal
3' distal
Intergenic

Fig S7

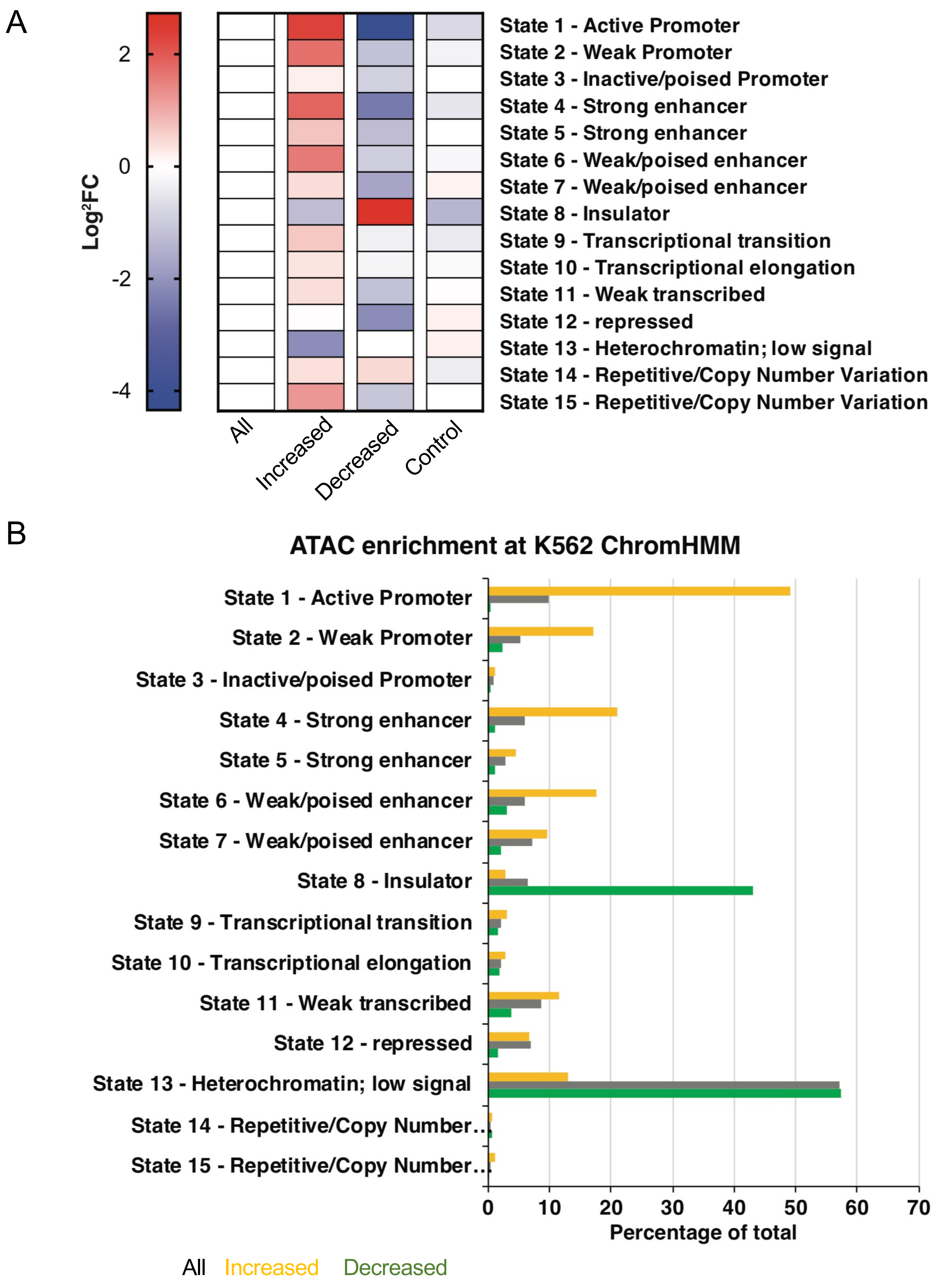
A
Log2FC
2
0
-2
-4
All
Increased
Decreased
Control
State 1 - Active Promoter
State 2 - Weak Promoter
State 3 - Inactive/poised Promoter
State 4 - Strong enhancer
State 5 - Strong enhancer
State 6 - Weak/poised enhancer
State 7 - Weak/poised enhancer
State 8 - Insulator
State 9 - Transcriptional transition
State 10 - Transcriptional elongation
State 11 - Weak transcribed
State 12 - repressed
State 13 - Heterochromatin; low signal
State 14 - Repetitive/Copy Number Variation
State 15 - Repetitive/Copy Number Variation
B
ATAC enrichment at K562 ChromHMM
State 1 - Active Promoter
State 2 - Weak Promoter
State 3 - Inactive/poised Promoter
State 4 - Strong enhancer
State 5 - Strong enhancer
State 6 - Weak/poised enhancer
State 7 - Weak/poised enhancer
State 8 - Insulator
State 9 - Transcriptional transition
State 10 - Transcriptional elongation
State 11 - Weak transcribed
State 12 - repressed
State 13 - Heterochromatin; low signal
State 14 - Repetitive/Copy Number...
State 15 - Repetitive/Copy Number...
0 10 20 30 40 50 60 70
Percentage of total
All Increased Decreased

Fig S8

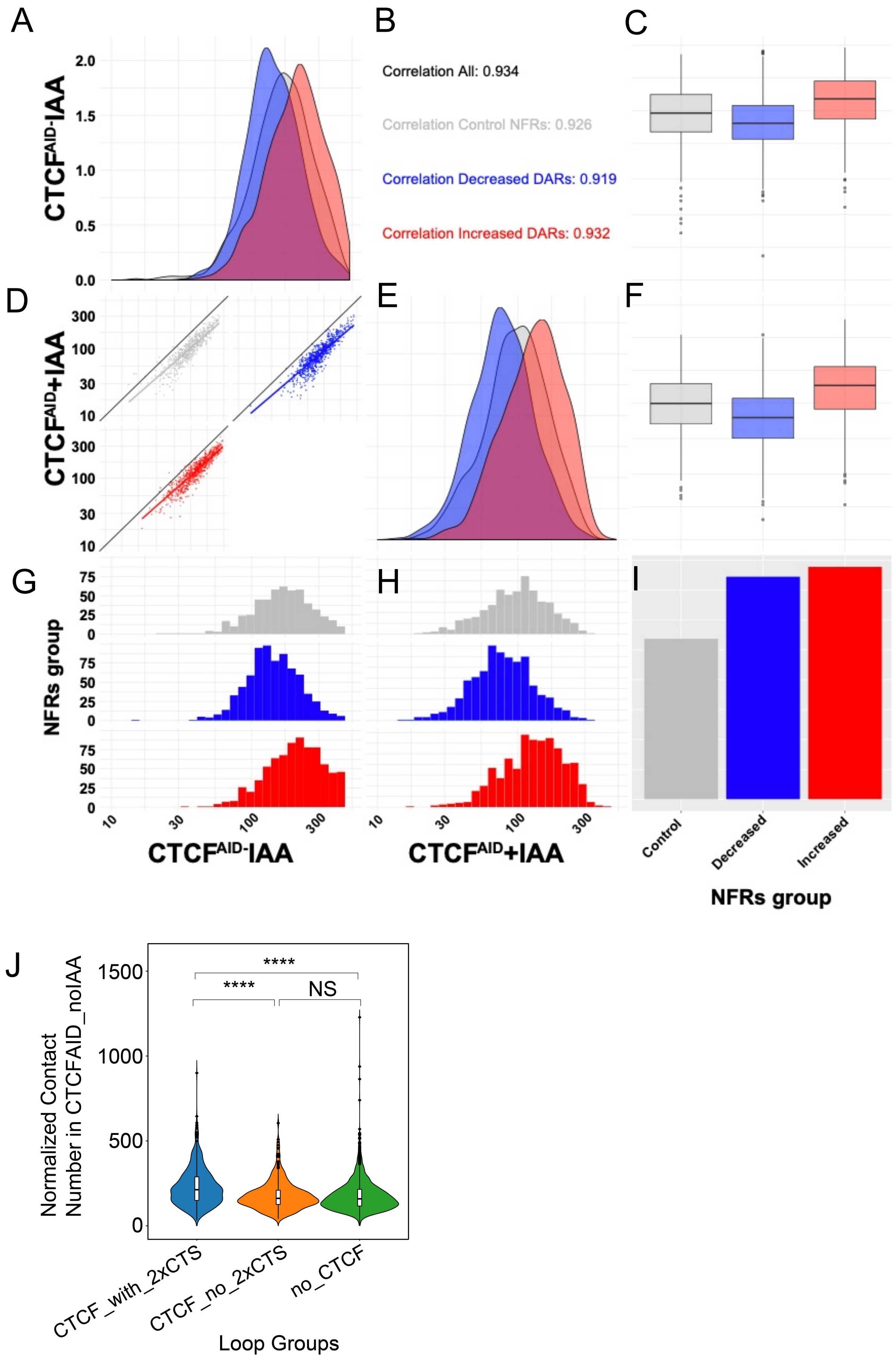
A
B
C
CTCF^AID-IAA
2.0
1.5
1.0
0.5
0.0
Correlation All: 0.934
Correlation Control NFRs: 0.926
Correlation Decreased DARs: 0.919
Correlation Increased DARs: 0.932
D
E
F
CTCF^AID+IAA
300
100
30
10
G
H
I
NFRs group
75
50
25
0
10
30
100
300
CTCF^AID-IAA
CTCF^AID+IAA
Control
Decreased
Increased
NFRs group
J
Normalized Contact Number in CTCFAID_noIAA
1500
1000
500
0
****
****
NS
CTCF_with_2xCTS
CTCF_no_2xCTS
no_CTCF
Loop Groups

Fig S9

A

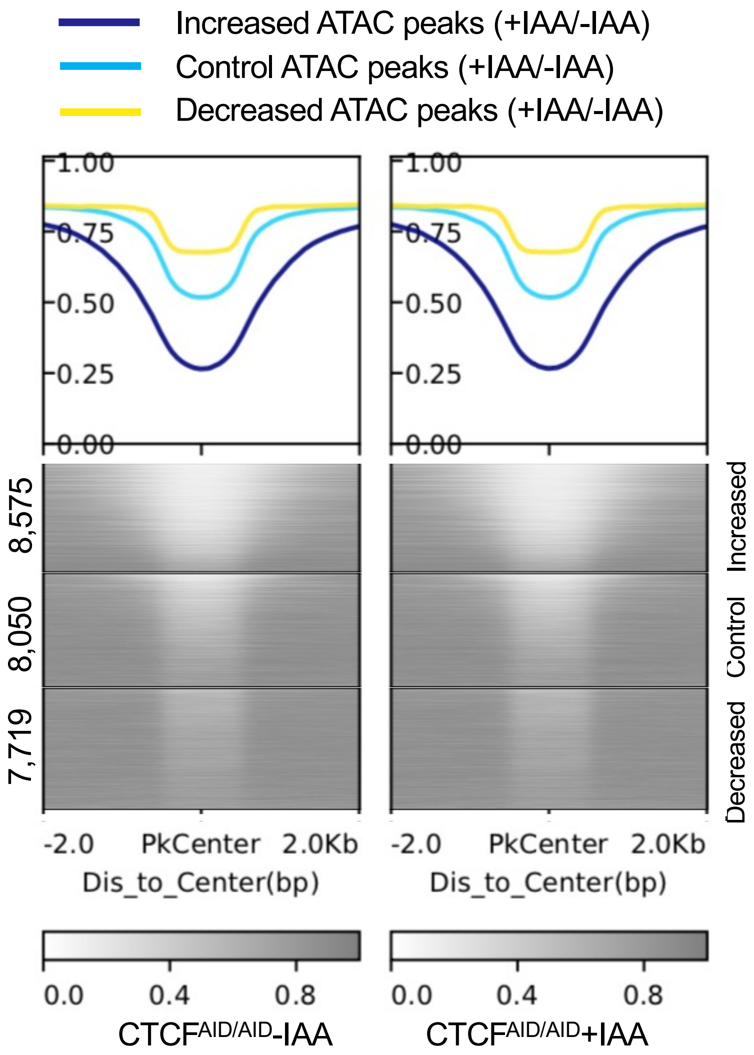

B

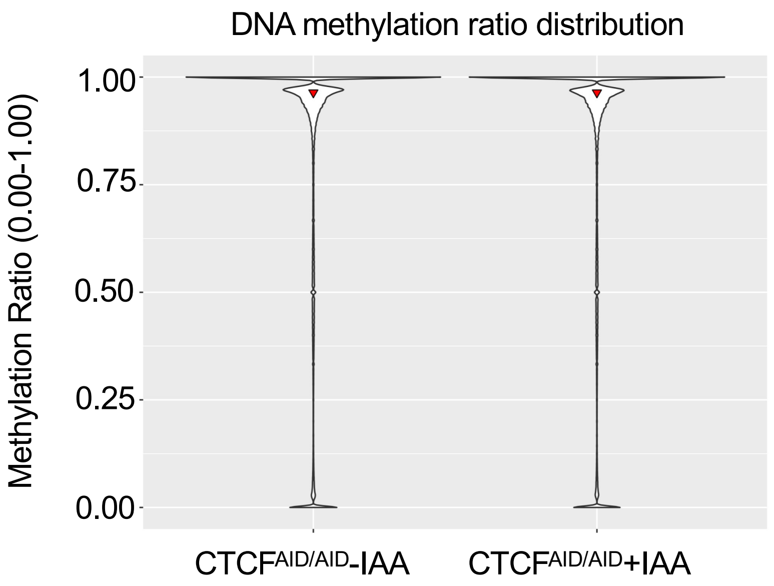

C

Rank	Motif	Name	P-value
1	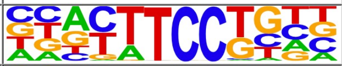	Etv2(ETS)/ES-ER71-ChIP-Seq(GSE59402)/Homer	1e-2
2	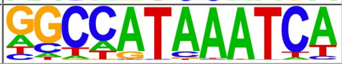	Hoxc9(Homeobox)/Ainv15-Hoxc9-ChIP-Seq(GSE21812)/Homer	1e-2

Fig S10

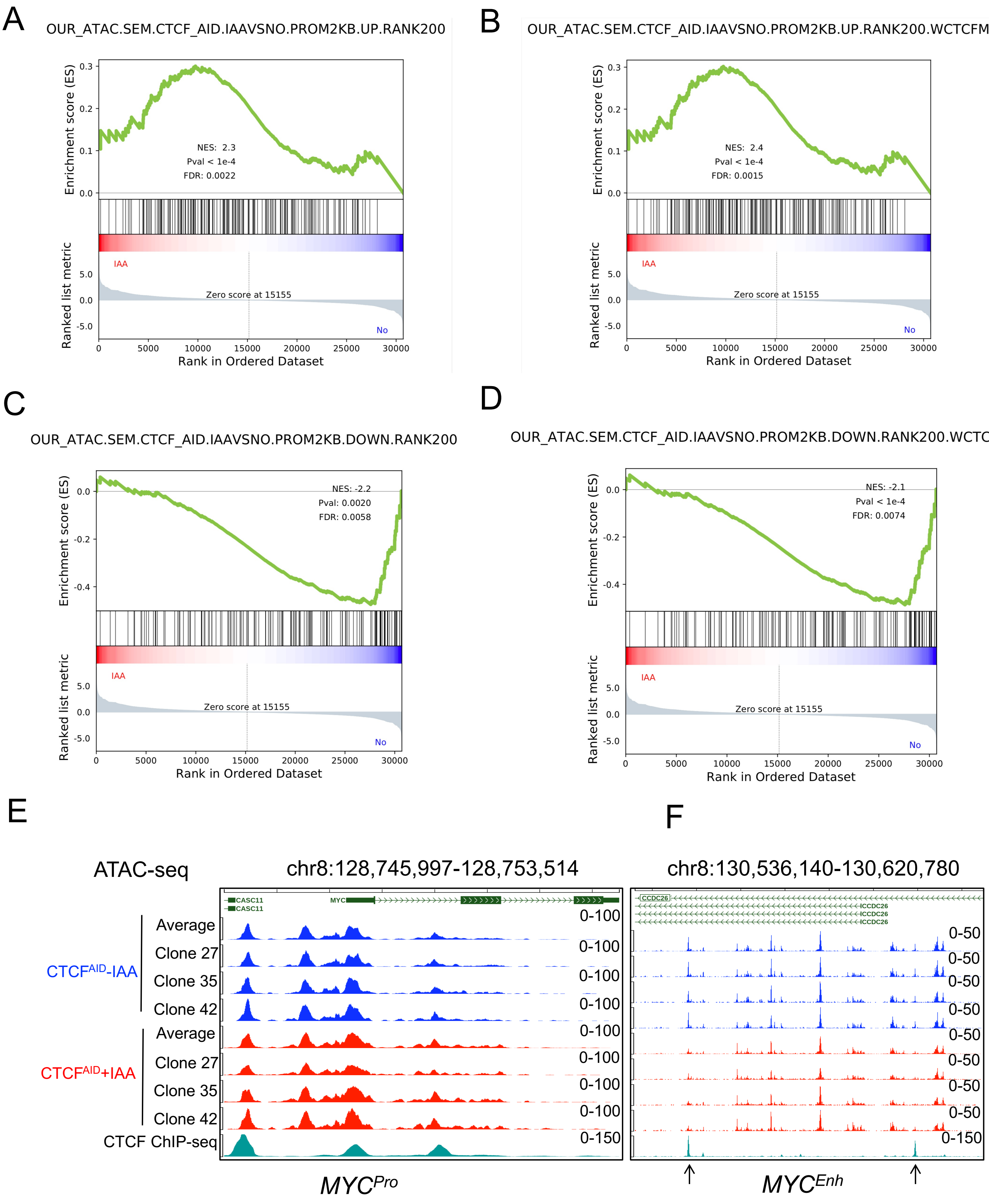
A
OUR_ATAC.SEM.CTCF_AID.IAAVSNO.PROM2KB.UP.RANK200
NES: 2.3
Pval < 1e-4
FDR: 0.0022
B
OUR_ATAC.SEM.CTCF_AID.IAAVSNO.PROM2KB.UP.RANK200.WCTCFMOTIF
NES: 2.4
Pval < 1e-4
FDR: 0.0015
C
OUR_ATAC.SEM.CTCF_AID.IAAVSNO.PROM2KB.DOWN.RANK200
NES: -2.2
Pval: 0.0020
FDR: 0.0058
D
OUR_ATAC.SEM.CTCF_AID.IAAVSNO.PROM2KB.DOWN.RANK200.WCTCFMOTIF
NES: -2.1
Pval < 1e-4
FDR: 0.0074
Enrichment score (ES)
Ranked list metric
IAA
No
Zero score at 15155
Rank in Ordered Dataset
E
ATAC-seq
chr8:128,745,997-128,753,514
CASC11
MYC
Average
Clone 27
Clone 35
Clone 42
CTCF^AID-IAA
CTCF^AID+IAA
CTCF ChIP-seq
0-100
0-150
MYC^Pro
F
chr8:130,536,140-130,620,780
CCDC26
0-50
0-150
MYC^Enh

Fig S11

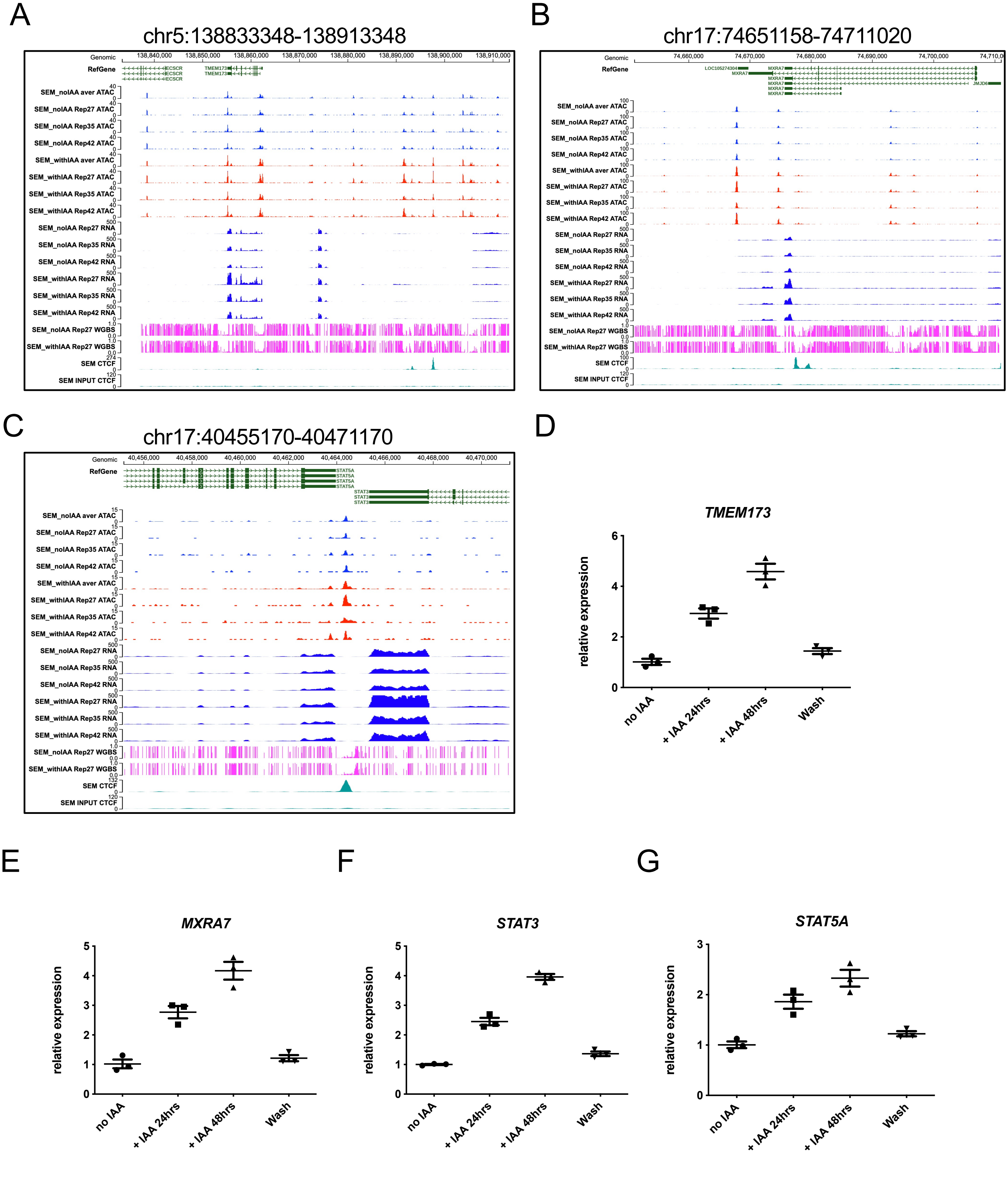
A
chr5:138833348-138913348
Genomic
RefGene
SEM_noIAA aver ATAC
SEM_noIAA Rep27 ATAC
SEM_noIAA Rep35 ATAC
SEM_noIAA Rep42 ATAC
SEM_withIAA aver ATAC
SEM_withIAA Rep27 ATAC
SEM_withIAA Rep35 ATAC
SEM_withIAA Rep42 ATAC
SEM_noIAA Rep27 RNA
SEM_noIAA Rep35 RNA
SEM_noIAA Rep42 RNA
SEM_withIAA Rep27 RNA
SEM_withIAA Rep35 RNA
SEM_withIAA Rep42 RNA
SEM_noIAA Rep27 WGBS
SEM_withIAA Rep27 WGBS
SEM CTCF
SEM INPUT CTCF
B
chr17:74651158-74711020
C
chr17:40455170-40471170
D
TMEM173
relative expression
no IAA
+ IAA 24hrs
+ IAA 48hrs
Wash
E
MXRA7
F
STAT3
G
STAT5A

Fig S12

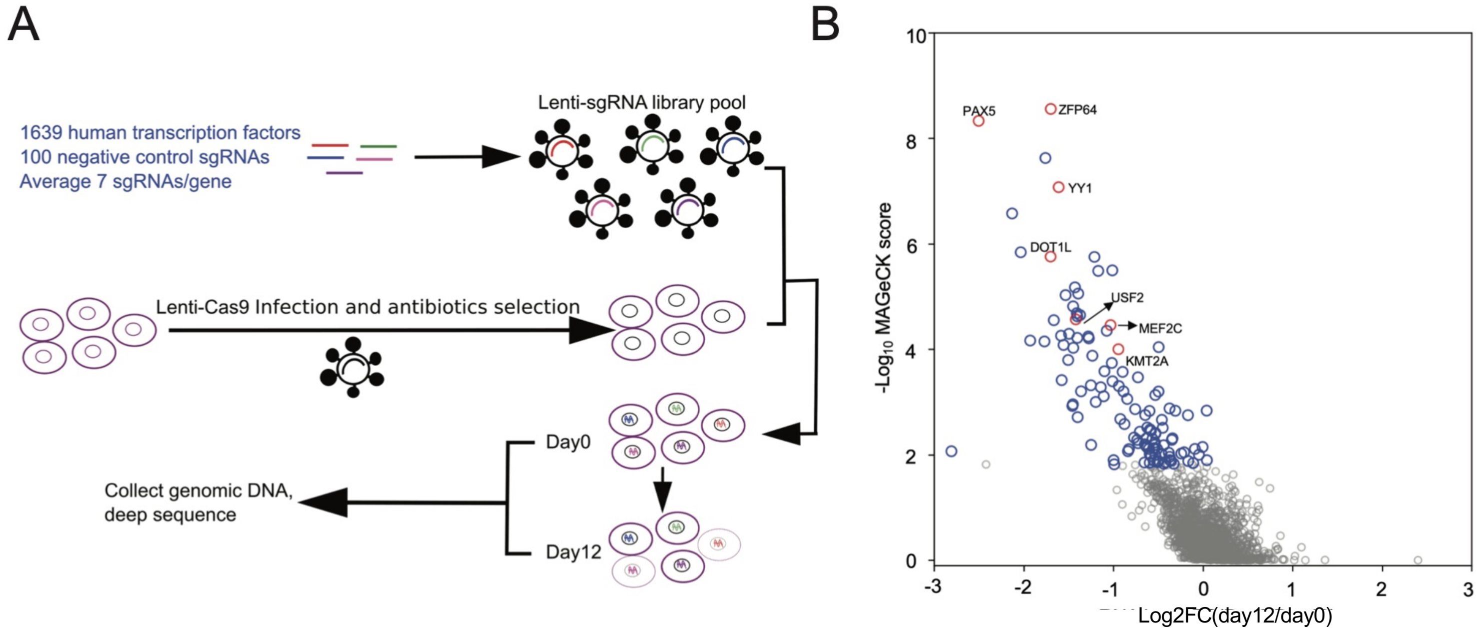
A
1639 human transcription factors
100 negative control sgRNAs
Average 7 sgRNAs/gene
Lenti-sgRNA library pool
Lenti-Cas9 Infection and antibiotics selection
Day0
Day12
Collect genomic DNA,
deep sequence
B
-Log10 MAGeCK score
Log2FC(day12/day0)
PAX5
ZFP64
YY1
DOT1L
USF2
MEF2C
KMT2A
10
8
6
4
2
0
-3
-2
-1
0
1
2
3

Fig S13

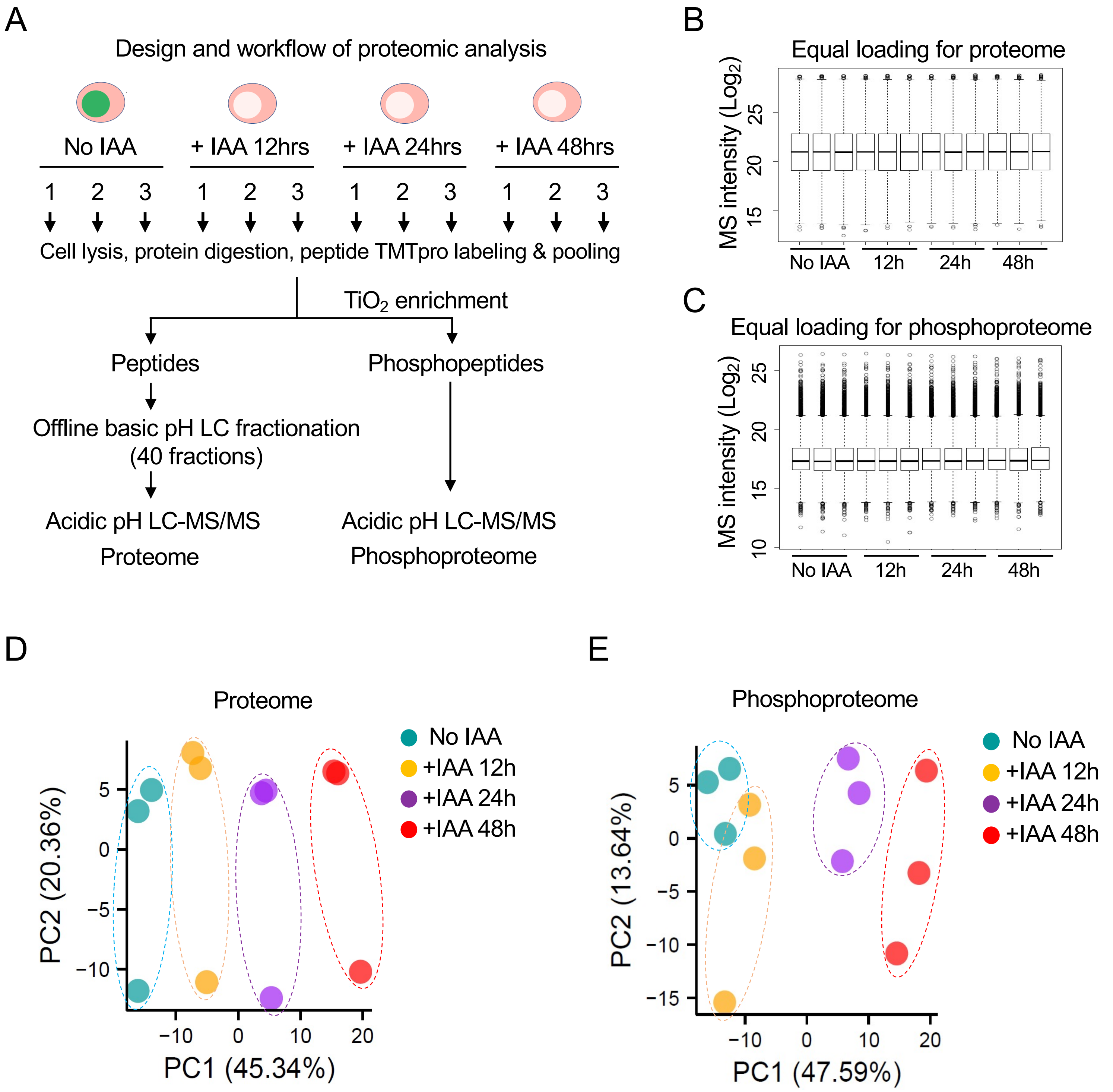

F

Data	Time course (IAA vs no IAA)	DE (FDR < 0.05)	Down	Up
Proteome	12h	529	282	247
	24h	2,550	1,183	1,367
	48h	4,891	2,104	2,787
Phosphoproteome	12h	54	42	12
	24h	1,895	994	901
	48h	3,913	1,805	2,108
