# Supplementary material for: Acute depletion of CTCF rewires genome-wide chromatin accessibility
Source: Genome Biol. 2021 Aug 24;22:244. doi: 10.1186/s13059-021-02466-0 (PMC8386078; doi:10.1186/s13059-021-02466-0)
Supplement: Supplementary file 9 — Additional file 9. [file 13059_2021_2466_MOESM9_ESM.docx]

**Review history**

**First round of review**

**Reviewer 1**

Xu et al. present a manuscript in which they characterize a CTCF-degron line using a number of omics techniques. The paper contains a number of interesting angles of investigation, however, they are not followed up by rigorous validation. Many of the observations have very small effect sizes. An interesting observation is that ZBTB7A seems to co-bind with CTCF. However, no follow up experiments are performed to nail down the mechanism. Therefore, in the end the reader is left wondering what have we learned about CTCF biology.

Major comments:
Without functional validation, many of the observed correlations remain that: correlations. The authors speculate in Fig. 5C that a CTCF site acts as an insulator. To validate this, the authors need to mutate the CTCF binding site and determine the effect on BLCAP expression.

The authors should have the paper read by an expert in both 3D genome and CTCF. Some statements:
"CTCF was originally identified by its ability to insulate the imprinted H19-IGF2 and β-hemoglobin loci(2,3)"
CTCF was identified by Filipova et al. in 1993 as a regulator of MYC
"CTCF-binding occupancy is highly enriched at all known scales of chromatin architecture"
What does this even mean? B compartments are not enriched for CTCF.
"Despite a global reduction of chromatin interactions upon CTCF loss, its effect on transcription is minimal"
Nora et al showed that 1000s of genes are differentially expressed. Hardly minimal.

Many more comments like this can be found throughout the paper.

The 2xCTS are suggested to be involved in looping and a paper that has no such analysis is used as a reference. Please show using the Hi-C data for SEM that these sites have a propensity to loop.

Other comments:

* Fig. 1E: how can the signal in the violin plot go below 0. Please make sure that the figure starts at 0.

* Fig. 2E: the difference may be significant with a very small p-value, but the effect size is very small. It does not seem to add much to the story and the analysis is better left out. If the authors should choose to leave it in a proper rationalization of the observed correlation should be given.

* Fig. 2C The author suggest the effects are directly related to CTCF, which is likely true, however the wording is ambiguous

* Figure S7 misses labels, making the figure unintelligible.

* l. 223: more open chromatin is often associated with more expression.
* Fig. 4D: the self-insulating role of CTCF can be easily checked by mutating the CTCF binding site in the promoter of CTCF. Otherwise, this could also be caused by pleiotropic (indirect) effects of CTCF depletion.

* How were the CTCF co-regulatory partners selected? Please describe briefly in the text.

**Reviewer 2**

In this manuscript, Xu et al. set out to decipher the role of CTCF in regulating chromatin accessibility and transcription. Towards this, they made use of acute CTCF depletion paradigm which is a widely accepted and preferred system over Knock-out or Knockdown strategies. While CTCF depletion leads to global changes in TADs, changes in transcription after 1 day of depletion are not very dramatic. Therefore, the question of whether CTCF and cohesin-mediated loop extrusion process collaborate to influence enhancer-promoter contacts and transcription remains an open question in the field. With the bioinformatic analyses, Xu et al. attempted to uncover differential positive and negative regulatory functions of CTCF in the context of chromatin accessibilities and transcription. Like other studies, authors could identify only a few deregulated genes upon CTCF depletion. In addition, the authors also generated proteome and phosphoproteome data upon acute depletion of CTCF in the SEM cells as a read out for gene expression changes. By integrating ATAC-seq, transcriptomics, proteomics and CRSIPR-drop out data, the authors identified 40 co-regulatory factors that work together with CTCF.
Altogether the study will be interesting for the field. I have a number a comments to help better the manuscript, most of which can be addressed by clarifying the manuscript / analyses. I recommend ultimate publication in Genome Biology.
Overall comments
(1)     There is no discussion of CRSIPR-drop out in the main text. This is an exciting experiment that needs to be explained and discussed at greater length in the main text, not just appear in part of a figure. The methods section also completely lacks information about the library used, the way the screen was conducted experimentally and how the data was analyzed. This is not acceptableAuthors need to include a table reporting their result with the statistical score for each sgRNA/gene assayed.

(2)     In the methods section it would be preferable if authors refrained from mentioning 'conducting as previously described' (e.g. for the CUT&RUN). It would be much more helpful if authors could incorporate the precise description of all the experiments carried out in this study, including all the details necessary for other investigators to replicate the experiments / analyses.

(3)     It would be relevant to include discussion of Owens et al. 2019 eLife who have analyzed the effect of acute CTCF depletion in mESCs on chromatin accessibility and nucleosome positioning

(4)     As per ENCODE standard guidelines for ATAC-seq data, it is recommended to include FRiP scores. No FRiP scores for the ATAC-seq data could be found in the manuscript. Please include these in supplementary information

Fig 1- General Comments
(5)     Entire premise for the study is acute CTCF depletion, however, CTCF depletion doesn't seem to be complete as per Hyle, J. et al. 2019 (Fig. S2 A-C). Although CTCF protein completely disappears when monitored by Western blotting, there is still some left-over as measured by Flow Cytometry (Hyle, J. et al. 2019). This should be acknowledged in the present study. Moreover, CTCF binding also isn't completely abrogated after 48h depletion as reported in Hyle, J. et al. 2019 (Fig. S2 A-C). CTCF peaks and their numbers are also quite variable between 3 clones generated in the Hyle et al. 2019 study and used in the current study (Clone 27, 35, 42). Is it because lentivirus mediated OsTir1 integration approach was used leading to random integration and could have led to different copy numbers in different clones leading to variability between CTCF peaks in these clones?

(6)     For the reasons stated in point 1, it would be very helpful to know if CTCF binding was completely abolished after 24h of depletion (by ChIP-seq), especially at the motifs close to the assigned DARs. While not strictly necessary it would allow much clearer interpretation of the ATAC-seq data.

(7)     Where do the increased or decreased DARs lie on the HiC map for SEM cells? Are the increased DARs close to the TAD boundaries?

Fig 1- Minor Comments
(8)     AID tagging is known to destabilize tagged proteins. Could authors provide a western blot with the CTCF levels in Control cells (parental line, -Dox)?
(9)     Logic of using USF1/2 knockdown as controls in Fig1? Where is the westerns to confirm the KD?
(10)     ATAC-seq was done in duplicates as per the description in Methods Section. The authors state that the results from both the replicates are well correlated but Spearman correlation plot for the 2 replicates couldn't be found in the manuscript. Please include in supplementary.
(11)     Are the control DAR peaks the one where CTCF binding persists? Or the peaks which has no CTCF motif/binding or inconsequential to CTCF binding?

Fig 2- Major Comments
(12)     Page 8, lines 152, 153 - Can the authors please elaborate on what do they mean when they say "reduced CTCF-dependent insulation allows more GTFs to bind target gene promoters" specifically in the case of Increased DARs.
(13)     In the case of Increased DARs, CTCF motifs are 100bp away from the ATAC-seq peak (Fig 1E). And most of the Increased DARs are at the gene promoters (Fig. S5). Are the authors suggesting that CTCF binding is preventing GTFs to bind their cognate sites on the gene promoters? Or do the authors mean that the CTCF sitting close to the promoter is preventing Enhancer-promoter interaction by serving as an insulator? And now that CTCF has been depleted, enhancers can interact with their respective promoters and increase the accessibility of the region as well as transcription as shown later?
(14)     Motifs for Control ATAC peaks in Fig. 2A are highlighted as increased ATAC peaks in Fig. 2B. What does this mean? And how is this possible?
(15)     As per Hyle et al. 2019, Myc was downregulated upon CTCF depletion in SEM cells for 48h. However, Myc motif isn't highlighted on the volcano plot for decreased DARs. The authors do state that it is one of the enriched motifs which is part of Supp Table but I am surprised to not see it on the Volcano plot in the Fig. 2A. It would be great if the authors can provide some clarification over this.

Fig 2- Minor Comments
(16)     Page 9 (lines 164-166) - Numbers of DARs used for the analysis in Fig 2C and Supp Fig 4 are different than Fig 1C

Fig 3- Major Comments
(17)     Could authors please provide justification for using only clone 27? As per Hyle et al. 2019, CTCF binding isn't completely lost even after 48h of CTCF depletion in this clone. Why is this clone may ideally suited for WGBS? Please elaborate on whether incomplete depletion might be a confounding factor. It is also important that authors clearly state that only one WGBS replicate was performed.

Fig 4 - Major Comments
(18)     In figure 4 it would be helpful if authors indicated the number of dysregulated genes (up and down separately) at the timepoint analyzed with the other experiments - ideally with a heatmap indicating their fold change. If data is available for each of the three clone, it would be helpful to display side by side.
(19)     In figure 4 It would be helpful if authors could show chromatin accessibility changes at the promoter of up- and down-regulated genes (separately) in the form of a heatmap like the one presented in figure 1C, where the number of up- and down- regulated genes promoters would be clearly indicated. Figures 4C and D could go in supplementary if space becomes limiting.

(20)     The authors determine chromatin accessibility as well as transcriptional changes for CTCF upon CTCF depletion and infer that "CTCF can insulate itself to maintain optimal levels". In my opinion, I do not think that just Fig 4A,C, D are sufficient to draw this inference. Possibility of CTCF binding to its own promoter and autoregulating itself in a negative feedback loop can not be ruled out just based on the results presented here.


(21)     In Fig. 4E, authors report that the accessibility at the Myc promoter doesn't change but the accessibility at its enhancer located 1.8Mb from the promoter decreases upon CTCF depletion, leading to dramatic downregulation of Myc. However, this is at odds with the results reported in Hyle et al. 2019, wherein the authors show that CTCF binding at both Myc promoter as well as enhancer goes down. The difference between Hyle et al. 2019 and current study is the duration of depletion (48h vs 24h). Could authors please provide insights into this? Interestingly, Myc protein is down by after 24h of CTCF depletion in Hyle et al. 2019.
I am curious to know if Myc promoter is one of those regions which is considered as Control DAR in the analysis presented in Fig 1 of the current study and doesn't show any changes in accessibility despite loss of CTCF binding?
And in a broader sense, did the authors find a set of deregulated genes that showed no changes in promoter and/or enhancer accessibility which has CTCF binding in the vicinity?

Fig 4 - Minor Comments
(22)     Is there a correlation between CTCF motif orientation and the transcription changes observed for the genes which had DARs and CTCF motif at the promoter?

Fig 6 - Major Comments
(23)     The manuscript could benefit from a clearer justification for conducting the proteome and phosphoproteome studies. Could the authors please elaborate on the logic of looking at proteome or phosphoproteome levels upon CTCF loss? Authors argue that the protein level changes are a better read out for gene expression changes rather than looking at transcriptional changes. I am unable to comprehend this, given the fact that the basis of the study is to identify chromatin accessibility changes and its correlation with transcriptional changes upon CTCF loss. While the bulk mRNA seq suffers from the limitations of differential mRNA stabilities and turn over, it would be a better idea to look at nascent transcription instead. Given how the manuscript is currently presented I fail to understand how studying the proteome or phosphoproteome help alleviate the limitation of RNA-seq.
(24)     Authors also mention that the translation machinery was also deregulated upon CTCF depletion which might have led to changes in the proteome. Doesn't this imply that the proteome change is more likely a secondary effect of their experimental system/condition.
(25)     The correlations between CTCF and co-regulatory partner motifs are difficult to understand as currently phrased (Fig. 6E). Are the authors suggesting that CTCF assists in co-regulatory factor binding? If so, wouldn't it be worth authors validate this by performing CUT&RUN for a few chosen co-regulatory factors upon CTCF depletion?
(26)     The authors posit that a correlation between the occurrence of CTCF motif and co-regulatory partner motif is more evident for Decreased DARs as compared to the Control or Increased DARs. Could the authors please clarify and elaborate upon following:
(i)     Most of decreased DARs are in the intronic regions (Fig. S5C). How many decreased DARs were incorporated in the analysis presented in Fig. 6E,F?
(27)     (ii)In Fig 2, Increased DARs were suggested to be the regions which has motifs enriched for General TFs and are withing 100 bp of CTCF motif. And these regions are more enriched in the gene promoters. Based on this, one would expect to see much stronger correlation between the CTCF and co-regulatory factor motifs for these Increased DARs.
(28)     Or do the authors think that all the CTCF sites at increased DARs are at the TAD boundary and serving as an insulator sites?
(29)     Did the authors identify any co-repressors motifs close to CTCF motif that might explain increased DARs upon CTCF loss and hence transcriptional upregulation?
(30)     In order to be classified as co-regulatory which is based on the correlations drawn from the data, it might be more meaningful to either deplete the co-regulatory factor(s) either individually and along with CTCF or delete the co-regulatory factor motifs from the selected sites and score for the transcriptional changes as a proof of principle.

Fig 6 - Minor Comments
(31)     Is there a significant overlap between proteome and phosphoproteome? If so, what does that mean in the context of CTCF depletion?

General Minor Comments
(32)     Page 3, line 33 - Not just human cohesin, also shown in mouse cells (mESC, CH12)
(33)      Page 3, line 36 - Change Ref 19 (Nora et al. 2020, Nat Comm)
(34)     Page 18, line 376 -There seems to be a mistake. The statement should be "increased" DARs upon CTCF loss exhibit tandem CTCF-binding pattern.
(35)     Page 21, lines 440, 447 - What do authors mean by occupancy switching? They cite an example of switching between CTCF and YY1 (line 447) but that doesn't seem likely considering the fact that CTCF and YY1 binding motifs are absolutely unique. However, CTCF and YY1 do interact with each other, so it is feasible that they are found at each other's motifs but that might not be the idea here.
(36)     In Supp Fig. 5, DAR numbers for the Venn diagrams slightly different from Fig 1C
(37)     Line 66 should read "our data" instead of "we data"
(38)     Line 67 should read "shedding light" not "a light"

**Authors’ response to reviewers**

**Reviewer #1**

Xu et al. present a manuscript in which they characterize a CTCF-degron line using a number of omics techniques. The paper contains a number of interesting angles of investigation, however, they are not followed up by rigorous validation. Many of the observations have very small effect sizes. An interesting observation is that ZBTB7A seems to co-bind with CTCF. However, no follow up experiments are performed to nail down the mechanism. Therefore, in the end the reader is left wondering what have we learned about CTCF biology.

**Major comments:**

Without functional validation, many of the observed correlations remain that: correlations. The authors speculate in Fig. 5C that a CTCF site acts as an insulator. To validate this, the authors need to mutate the CTCF binding site and determine the effect on BLCAP expression.

Thank you for the reviewer’s comments. We agree with the reviewer that targeting the CTCF binding site (CBS) would provide the most direct evidence to link CTCF function with transcriptional regulation of *BLCAP*. To address this concern, we conducted the following experiments to investigate functional regulation. **First**, we designed a guide RNA targeting the CBS within the CTCF binding peak in the distal non-coding region upstream of the *BLCAP* gene promoter. Lentiviral-expressing guide RNA was infected into Cas9-expressing SEM cells followed with antibiotic selection. Sanger genomic sequencing (TIDE-seq) detected about 61% overall indel frequency in the targeted pool population, which led to a significant increase of *BLCAP* mRNA expression compared with the non-targeting control (sgNT). **Second**, since there is only one CTCF binding peak in the distal non-coding region upstream of the *BLCAP* gene promoter, we believe acute depletion of CTCF protein should provide a complementary result to further support the functional regulation of the CTCF/BLCAP axis. Therefore, RNA-seq analysis and Q-PCR validation were conducted to examine the mRNA expression of *BLCAP* in response to acute depletion of CTCF protein. As expected, the *BLCAP* mRNA expression level significantly increased upon acute depletion of CTCF protein by auxin treatment for 24 or 48 hours. More importantly, the expression level was restored to the level in parental cells after auxin washout. In summary, these data strongly support that the CTCF occupancy at the distal non-coding regulatory region of *BLCAP* serves as a functional insulator controlling transcription.


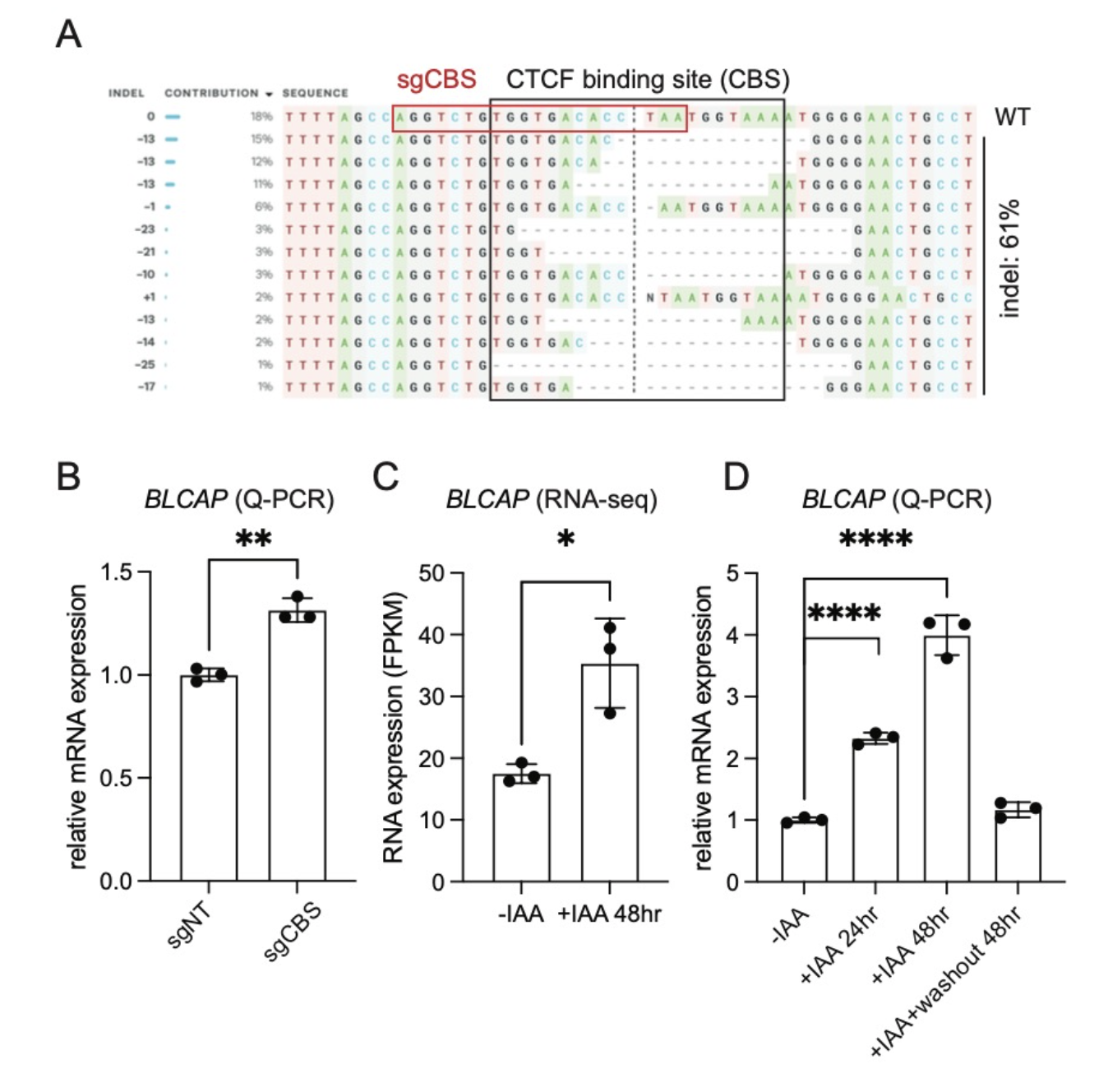


The authors should have the paper read by an expert in both 3D genome and CTCF. Some statements: "CTCF was originally identified by its ability to insulate the imprinted H19-IGF2 and β-hemoglobin loci (2,3)". CTCF was identified by Filipova et al. in 1993 as a regulator of MYC.

Thank you for the reviewer’s comments. We apologize for the inaccurate interpretation. We want to list an example that the insulation function of CTCF was originally identified to regulate H19-IGF2 and β-hemoglobin loci. We agree with the reviewer that transcriptional regulation of CTCF was first identified in *MYC* regulation in 1993. We corrected this description in the text accordingly.

"CTCF-binding occupancy is highly enriched at all known scales of chromatin architecture" What does this even mean? B compartments are not enriched for CTCF.

Thank you for the reviewer’s comments. We intend to emphasize that CTCF-binding occupancy is enriched in promoter/enhancer looping hubs and TAD boundaries. We did not mean to refer CTCF binding to compartment regulation. We corrected the sentence in the text to make the description accurate.

"Despite a global reduction of chromatin interactions upon CTCF loss, its effect on transcription is minimal". Nora et al showed that 1000s of genes are differentially expressed. Hardly minimal. Many more comments like this can be found throughout the paper.

Thank you for the reviewer’s comments. We agree that “minimal” is a vague description. Our observation suggests that the differentially expressed genes upon acute depletion of CTCF are less than that were depleted of CTCF binding occupancy at a genome-wide scale. We have carefully revised the interpretation throughout the paper.

The 2xCTS are suggested to be involved in looping and a paper that has no such analysis is used as a reference. Please show using the Hi-C data for SEM that these sites have a propensity to loop.

Thank you for the reviewer’s comments. We found that 775 out of 3,029 loops have both CTCF peaks and 2xCTS at least in one looping anchor. We also found that 333 loops have CTCF peaks but not 2xCTS. The normalized contact number from the loops with 2xCTS were significantly higher than those without 2xCTS (p = 2.9e-14, t-test). We include these results in Supplementary Figure 8J.

**Other comments:**

* Fig. 1E: how can the signal in the violin plot go below 0. Please make sure that the figure starts at 0.

Thank you for the reviewer’s comments. We apologize for the confusion. We confirmed that there is no value below 0. The original plot was generated and presented due to the usage of kernel density estimation. We have replaced it with an updated Fig. 1E.

* Fig. 2E: the difference may be significant with a very small p-value, but the effect size is very small. It does not seem to add much to the story and the analysis is better left out. If the authors should choose to leave it in a proper rationalization of the observed correlation should be given.

Thank you for the reviewer’s comments. We agree with the reviewer’s suggestion that removing this piece of data will keep the story focused.

* Fig. 2C The author suggest the effects are directly related to CTCF, which is likely true, however the wording is ambiguous.

Thank you for the reviewer’s comments. Although we are confident with our results, we also agree with the reviewer that there might be other possibilities. We revised the wording to stay focused on the observations evidentially supported.

* Figure S7 misses labels, making the figure unintelligible.

We apologize for the confusion. We now provide correct labels in the figure and details in the figure legend.

* l. 223: more open chromatin is often associated with more expression.

Thank you for the reviewer’s comments. We agree the previous description was vague. We agree with the interpretation “more open chromatin is often associated with more expression” as suggested (see line 217).

* Fig. 4D: the self-insulating role of CTCF can be easily checked by mutating the CTCF binding site in the promoter of CTCF. Otherwise, this could also be caused by pleiotropic (indirect) effects of CTCF depletion.

Thank you for the reviewer’s comments. We agree with the reviewer that targeting the CTCF binding site (CBS) would provide the most direct evidence to link CTCF’s regulatory function with transcriptional regulation of *BLCAP*. Please see our detailed response to the “major comments” previously.

* How were the CTCF co-regulatory partners selected? Please describe briefly in the text.

Thank you for the reviewer’s comments. We first required that CTCF co-regulatory partners (transcription factors, TFs) show significant enrichment of deregulated downstream genes either with up-regulated or down-regulated pattern of RNA and/or protein levels upon CTCF loss. Next, we filtered these TFs to ones with evidence of protein level expression supported by the mass-spectrum assay. Candidate TFs were further prioritized by their mRNA, protein and/or phosphorylation changes upon CTCF loss. In total, we identified 40 CTCF co-regulatory partner TFs that are significantly affecting their downstream gene expression at mRNA and/or protein level upon acute CTCF loss.

**Reviewer #2**

Comments for Xu et al.
In this manuscript, Xu et al. set out to decipher the role of CTCF in regulating chromatin accessibility and transcription. Towards this, they made use of acute CTCF depletion paradigm which is a widely accepted and preferred system over Knock-out or Knockdown strategies. While CTCF depletion leads to global changes in TADs, changes in transcription after 1 day of depletion are not very dramatic. Therefore, the question of whether CTCF and cohesin-mediated loop extrusion process collaborate to influence enhancer-promoter contacts and transcription remains an open question in the field. With the bioinformatic analyses, Xu et al. attempted to uncover differential positive and negative regulatory functions of CTCF in the context of chromatin accessibilities and transcription. Like other studies, authors could identify only a few deregulated genes upon CTCF depletion. In addition, the authors also generated proteome and phosphoproteome data upon acute depletion of CTCF in the
SEM cells as a read out for gene expression changes. By integrating ATAC-seq, transcriptomics, proteomics and CRSIPR-drop out data, the authors identified 40 co-regulatory factors that work together with CTCF.
Altogether the study will be interesting for the field. I have a number a comments to help better the manuscript, most of which can be addressed by clarifying the manuscript / analyses. I recommend ultimate publication in Genome Biology.

Thank you for the reviewer’s positive comments and constructive suggestions to further strengthen our manuscript. We appreciate the efforts that the reviewer has spent on helping us. We have carefully addressed each question and provided a point-by-point response below.

Overall comments
(1)     There is no discussion of CRSIPR-drop out in the main text. This is an exciting experiment that needs to be explained and discussed at greater length in the main text, not just appear in part of a figure. The methods section also completely lacks information about the library used, the way the screen was conducted experimentally and how the data was analyzed. This is not acceptable. Authors need to include a table reporting their result with the statistical score for each sgRNA/gene assayed.

Thank you for the reviewer’s comments. We apologize for the missing detail of the dropout CRISPR screen. To unbiasedly reveal the survival dependency genes in SEM cells, we analyzed data from a dropout CRISPR/Cas9 screen by targeting 1,639 transcription factors with 7 sgRNAs designed against each gene. The Cas9-expressing SEM cells infected with the pooled library of sgRNAs (M.O.I=~0.3) were collected at day 0 and day 12 to sequence for sgRNA redundancy (Fig. 5A). The rationale of this screen is based on the fact that read counts of sgRNAs against essential survival genes will be depleted on day 12 compared with day 0. Following the instruction of MAGeCK analysis, there are 117 TFs identified as essential survival genes based on the cutoff of MAGeCK score less than 0.01. Many survival dependent genes were identified among this gene list, including PAX5, DOT1L, ZFP64, YY1, MEF2C, KMT2A, and USF2 (Gu et al., 2019; Hyle et al., 2019; Pridans et al., 2008; Lu et al., 2018; Zhang et a., 2020). The method, experimental detail, and results have been updated in the text.


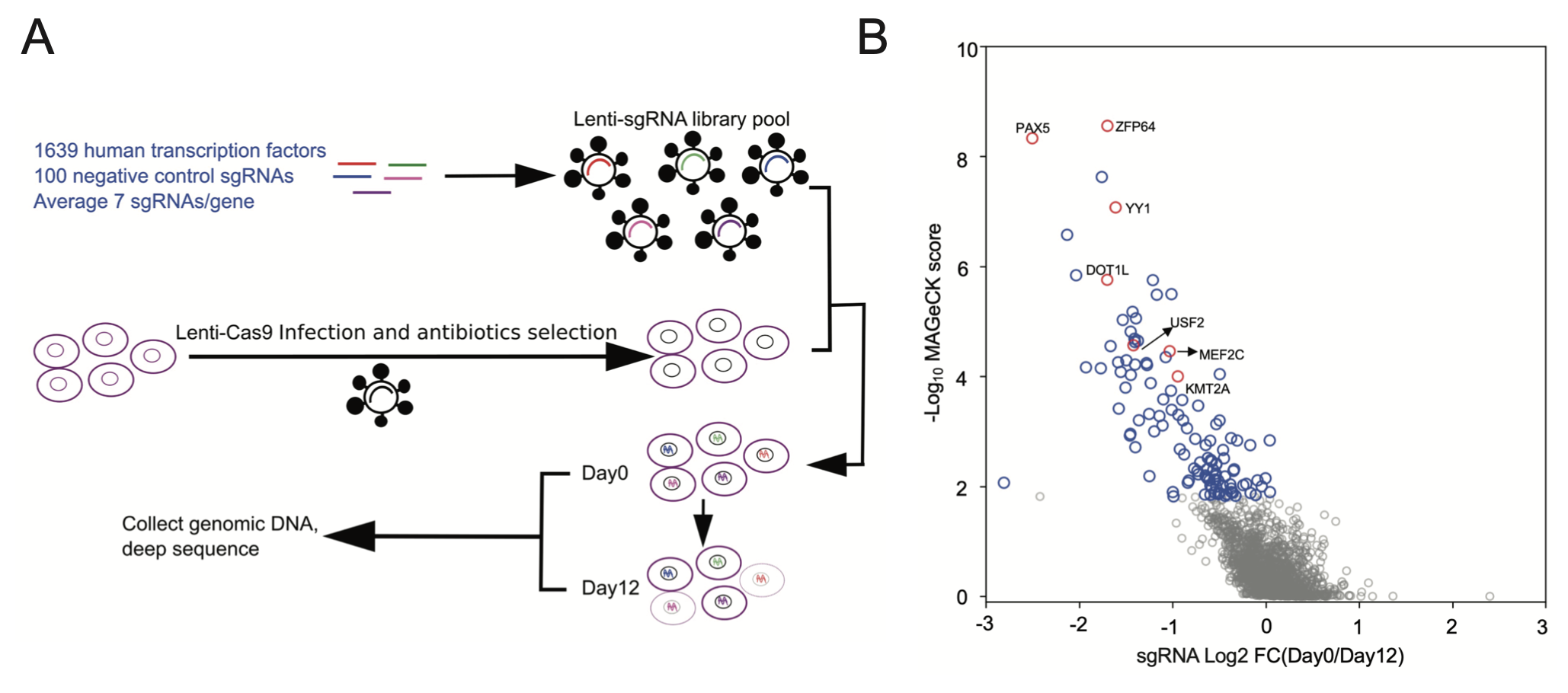


(2)     In the methods section it would be preferable if authors refrained from mentioning 'conducting as previously described' (e.g. for the CUT&RUN). It would be much more helpful if authors could incorporate the precise description of all the experiments carried out in this study, including all the details necessary for other investigators to replicate the experiments / analyses.

Thank you for the reviewer’s comments. We apologize for the missing detail of some assays and analysis. In addition to referring to the original publications, we have revised the method/material section and included necessary information for other investigators to replicate the experiments and analysis. The CUT&RUN data were from the previous study, so we did not include experimental detail for the assay.

(3)     It would be relevant to include discussion of Owens et al. 2019 eLife who have analyzed the effect of acute CTCF depletion in mESCs on chromatin accessibility and nucleosome positioning

Thank you for the reviewer’s comments. We apologize for missing the work published by Owens et al. 2019 at eLife. We have included this relevant reference in the discussion.

(4)     As per ENCODE standard guidelines for ATAC-seq data, it is recommended to include FRiP scores. No FRiP scores for the ATAC-seq data could be found in the manuscript. Please include these in supplementary information

Thank you for the reviewer’s comments and suggestions. Our FRiP (range from ~35% to ~44%) were larger than ENCODE suggested (30% recommended, 20% acceptable). We have also carefully checked the nucleosome pattern tracks, surrounding nucleosome-free tracks, and other ENCODE criteria. We concluded that the data quality of our ATAC-seq is great. We have provided quality control analysis of ATAC-seq data along with FRiP scores in Supplementary Table S1.

Fig 1- General Comments
(5)     Entire premise for the study is acute CTCF depletion, however, CTCF depletion doesn't seem to be complete as per Hyle, J. et al. 2019 (Fig. S2 A-C). Although CTCF protein completely disappears when monitored by Western blotting, there is still some left-over as measured by Flow Cytometry (Hyle, J. et al. 2019). This should be acknowledged in the present study. Moreover, CTCF binding also isn't completely abrogated after 48h depletion as reported in Hyle, J. et al. 2019 (Fig. S2 A-C). CTCF peaks and their numbers are also quite variable between 3 clones generated in the Hyle et al. 2019 study and used in the current study (Clone 27, 35, 42). Is it because lentivirus mediated OsTir1 integration approach was used leading to random integration and could have led to different copy numbers in different clones leading to variability between CTCF peaks in these clones?

Thank you for the reviewer’s comments. We acknowledge that residual CTCF protein can be detected upon auxin treatment due to the detection sensitivity of different strategies. For instance, although CTCF protein seems to completely disappear when monitored by immunoblotting, flow cytometry and CUT&RUN still can detect some positive signals. There are many possible explanations to address this observation. Dr. Gerd Blobel’s group recently reported that a minimal amount of chromatin-bound CTCF is retained upon auxin treatment (Nature, 576, pages158-162,2019). Also, random integration of different copies of OsTir1 may lead to a variable expression level of OsTir1. Finally, clonal variation of genetically engineered CTCF^AID^ lines may also contribute to incomplete protein degradation.

(6)     For the reasons stated in point 1, it would be very helpful to know if CTCF binding was completely abolished after 24h of depletion (by ChIP-seq), especially at the motifs close to the assigned DARs. While not strictly necessary it would allow much clearer interpretation of the ATAC-seq data.

Thank you for the reviewer’s comments. To address this question, we have conducted the auxin treatment for 24 and 48 hours to degrade CTCF protein in all three single-cell derived clones. We observed the complete degradation in clones 35 and 42, either 24 or 48 hours post auxin treatment. In clone 27, we have detected minimal residue CTCF protein after 24 hours but not 48 hours post auxin treatment. These data confirmed the clonal variation existed in the single-cell-derived knockin clones. However, in this study, we focused on the reproducible ATAC-seq peaks, which would reduce the impact from clonal variation. To further confirm our observation of chromatin accessibility change upon CTCF loss, we conducted ATAC-seq again on clones 27, 35 and 42 with auxin treatment for 48 hours. We observed consistent chromatin accessibility change compared with 24 hours focusing on the same groups of DARs.


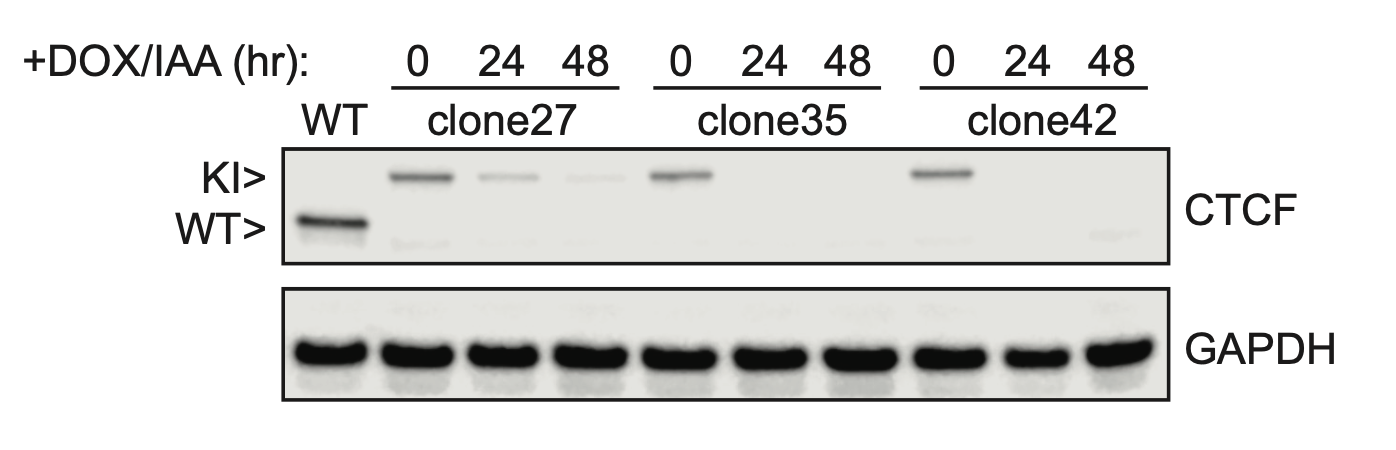


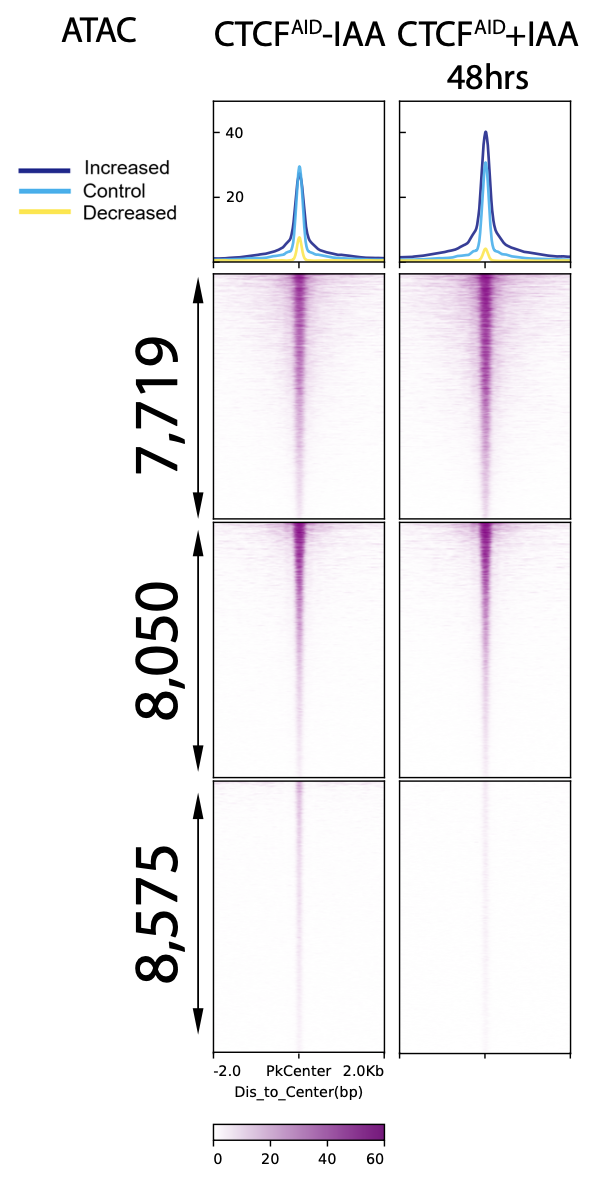


(7)     Where do the increased or decreased DARs lie on the HiC map for SEM cells? Are the increased DARs close to the TAD boundaries?

Thank you for the reviewer’s comments. We performed additional analysis and found that increased DARs were significantly distant from TAD boundaries than decreased DARs and control ATAC-seq peaks. However, we did not include these data in our current study due to the low sequencing depth for the HiC experiment. Also, these results might be limited due to 1) TADs were also experiencing dynamics upon CTCF degradation. 2) we only called high confidence TAD boundaries using the method from the previous study (Crane et al., Nature 2015), by which only 500~600 TAD boundaries were identified with confidence. We have replaced Fig. 2F with the density plot to reflect the difference.

Fig 1- Minor Comments
(8)     AID tagging is known to destabilize tagged proteins. Could authors provide a western blot with the CTCF levels in Control cells (parental line, -Dox)?

Thank you for the reviewer’s comments. We performed immunoblotting to compare the CTCF expression levels between parental SEM cells and the three knockin clones (clones 27, 35 and 42)(see new Fig. 1B). However, the expression level of CTCF^AID^ in three clones seems to be much lower than parental bulk populations. It is still difficult to conclude since the molecular weight is different, and the difference between the single cell-derived clones and bulk population also matters. However, given all three clones under long-term *in vitro* culture are indistinguishable from wild-type parental cells, we do not think severe destabilization of tagged CTCF protein occurs in our cellular system.


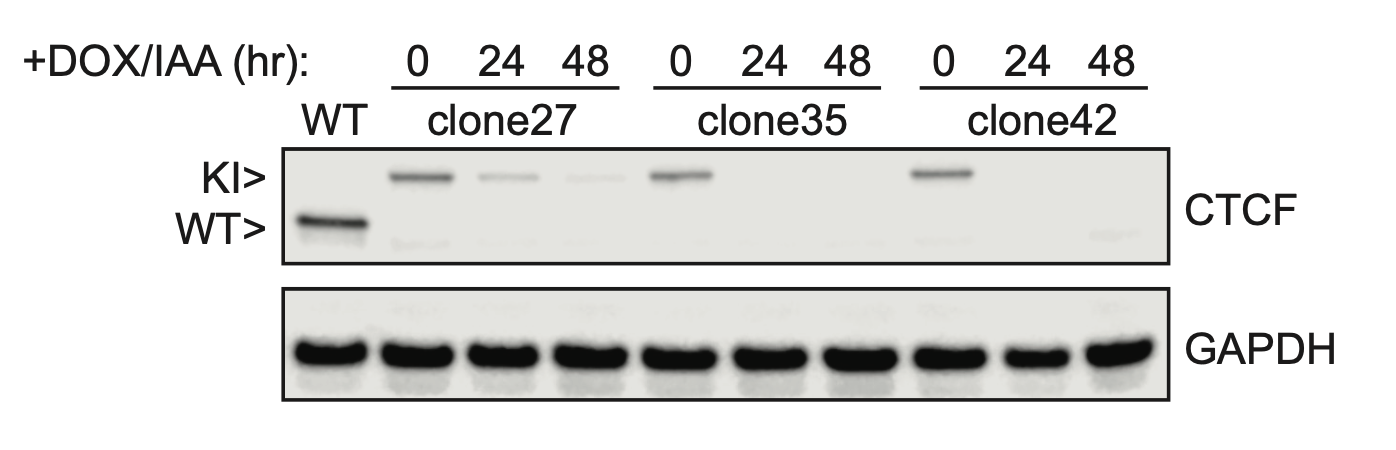


(9)     Logic of using USF1/2 knockdown as controls in Fig1? Where is the westerns to confirm the KD?

Thank you for the reviewer’s comments. We included DARs collected from USF1/2 knockdown to support the conclusion that CTCF regulated DARs were specific to CTCF loss. We also included immunoblotting results to confirm the successful knockdown of USF1/2 (see Supplementary Figure 1B).


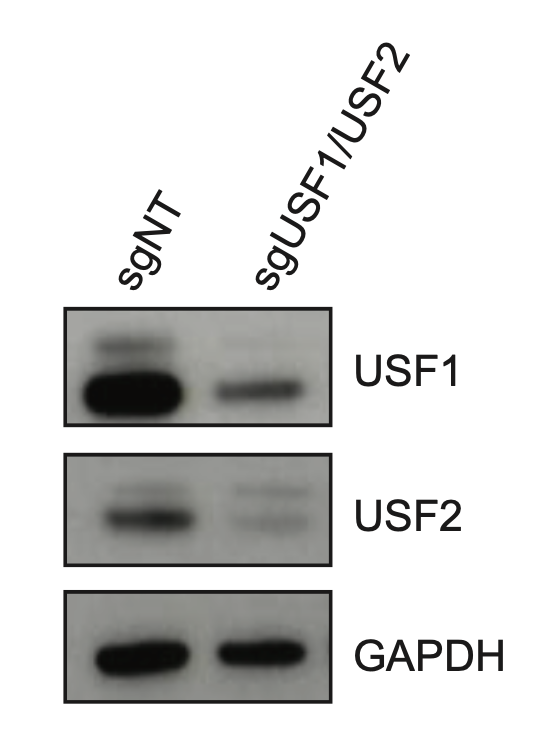


(10)    ATAC-seq was done in duplicates as per the description in Methods Section. The authors state that the results from both the replicates are well correlated but Spearman correlation plot for the 2 replicates couldn't be found in the manuscript. Please include in supplementary.

Thank you for the reviewer’s comments. We include the Spearman correlation plot result in Supplementary Figure S1.

(11)    Are the control DAR peaks the one where CTCF binding persists? Or the peaks which has no CTCF motif/binding or inconsequential to CTCF binding?

Thank you for the reviewer’s comments. We define “control DAR peaks” as those that don’t show significant chromatin accessibility change (p > 0.5) and fold change < 1.05. As shown in the ATAC-seq density heatmap, the “control DAR peaks” demonstrated lower CTCF binding affinity (Fig. 1C), less CTCF motif enrichment (Fig. 2A), and a more distant location from CTCF motifs (Fig. 1E).

Fig 2- Major Comments
(12)    Page 8, lines 152, 153 - Can the authors please elaborate on what do they mean when they say "reduced CTCF-dependent insulation allows more GTFs to bind target gene promoters" specifically in the case of Increased DARs.

Thank you for the reviewer comments. We apologize for the vague description that might confuse others. We propose CTCF behaves as a pioneer factor in practicing repressive function at increased DARs in control cells, by blocking certain GTF binding occupancy at the target gene promoter. Thus, upon CTCF loss, more GTFs would bind at these gene promoters, further increasing the chromatin accessibility. We revised the writing to deliver these messages in the text better.

(13)    In the case of Increased DARs, CTCF motifs are 100bp away from the ATAC-seq peak (Fig 1E). And most of the Increased DARs are at the gene promoters (Fig. S5). Are the authors suggesting that CTCF binding is preventing GTFs to bind their cognate sites on the gene promoters? Or do the authors mean that the CTCF sitting close to the promoter is preventing Enhancer-promoter interaction by serving as an insulator? And now that CTCF has been depleted, enhancers can interact with their respective promoters and increase the accessibility of the region as well as transcription as shown later?

Thank you for the reviewer’s comments and help with the interpretation of our data. This question is closely related to question #12 above. Our data likely fit the first mechanism as the reviewer pointed out, suggesting CTCF binding prevents GTFs from binding their cognate sites on the gene promoters. However, we could not completely exclude the second possibility. We apologize for the vague description that might confuse others. We have revised the interpretation of these data in the manuscript as suggested by the reviewer.

(14)    Motifs for Control ATAC peaks in Fig. 2A are highlighted as increased ATAC peaks in Fig. 2B. What does this mean? And how is this possible?

Thank you for the reviewer’s comments. We apologize for the confusion. This analysis was not used to evaluate whether these motifs have strong ATAC-seq signals (Fig. 2C and 2D). Instead, using the DAR information compared with the publicly available ChIP-seq database, we sought to explore which transcription factor binding signatures were enriched (more frequently appeared) in DARs than those control ATAC-seq peaks. For example, CNOT3 was enriched in control ATAC-seq peaks (~10%) compared to decreased DARs (~2%). It was also enriched in increased DARs (~50%) compared to control ATAC-seq peaks (~10%). Thus, one motif highlighted in control ATAC peaks in Fig. 2A could also be highlighted as increased ATAC-seq peaks in Fig. 2B.

(15)    As per Hyle et al. 2019, Myc was downregulated upon CTCF depletion in SEM cells for 48h. However, Myc motif isn't highlighted on the volcano plot for decreased DARs. The authors do state that it is one of the enriched motifs which is part of Supp Table but I am surprised to not see it on the Volcano plot in the Fig. 2A. It would be great if the authors can provide some clarification over this.

Thank you for the reviewer’s comments. We apologize for the inaccurate interpretation. The result we presented here is based on ChEA enrichment analysis, not motif analysis. By comparing genes assigned to closest DARs with publicly available ChIP-seq data from the ChEA database, the analysis output shows candidate TFs based on the chance of enrichment compared to random events. We realized CTCF was significantly enriched and likely to be the pioneer factor responsible for maintaining chromatin accessibility. Compared with CTCF, the enrichment of other TFs in decreased DARs is much weaker. In addition, we found it is also hard to reversely enrich the Myc motif even with the context of actual Myc target genes. For example, we took the top 100 Myc direct target genes detected by SLAM-seq (Science. 2018 May 18;360(6390):800-805) and tried different cutoffs for motif analysis. We could not enrich the MYC motif at the top. Together, we think for unbiased ChEA enrichment analysis, it is possible that motif for particular TF of interest would not enrich at the genome-wide scale.

Fig 2- Minor Comments
(16)    Page 9 (lines 164-166) - Numbers of DARs used for the analysis in Fig 2C and Supp Fig 4 are different than Fig 1C

Thank you for the reviewer’s comments. We apologize for the confusion. The numbers following the motif ID in Fig. 2C and Supplementary Figure S4 are not DAR numbers. They are numbers matched to the indicated motifs. We have revised the interpretation in text and figure legends.

Fig 3- Major Comments
(17)    Could authors please provide justification for using only clone 27? As per Hyle et al. 2019, CTCF binding isn't completely lost even after 48h of CTCF depletion in this clone. Why is this clone may ideally suited for WGBS? Please elaborate on whether incomplete depletion might be a confounding factor. It is also important that authors clearly state that only one WGBS replicate was performed.

Thank you for the reviewer’s comments. We acknowledge that in all clones, there is residual CTCF protein upon auxin induction and clonal variation. However, the RNA-seq and ATAC-seq suggest consistent results were seen among the three clones. Therefore, we do not think the incomplete depletion significantly impacts the results. Therefore, we randomly selected clone 27 for the WGBS test. We agree that more WGBS assays from more clones treated with auxin at different time points will provide more information. However, we would not conduct these experiments in this study due to the cost and time issue. We can move these data to supplementary Figure S9.

Fig 4 - Major Comments
(18)    In figure 4 it would be helpful if authors indicated the number of dysregulated genes (up and down separately) at the timepoint analyzed with the other experiments - ideally with a heatmap indicating their fold change. If data is available for each of the three clone, it would be helpful to display side by side.

Thank you for the reviewer’s comments. According to the reviewer’s suggestion, we generated a heatmap to indicate the transcriptional change of up and down-regulated genes is consistent with the change of ATAC-seq signals at promoters in all three clones.


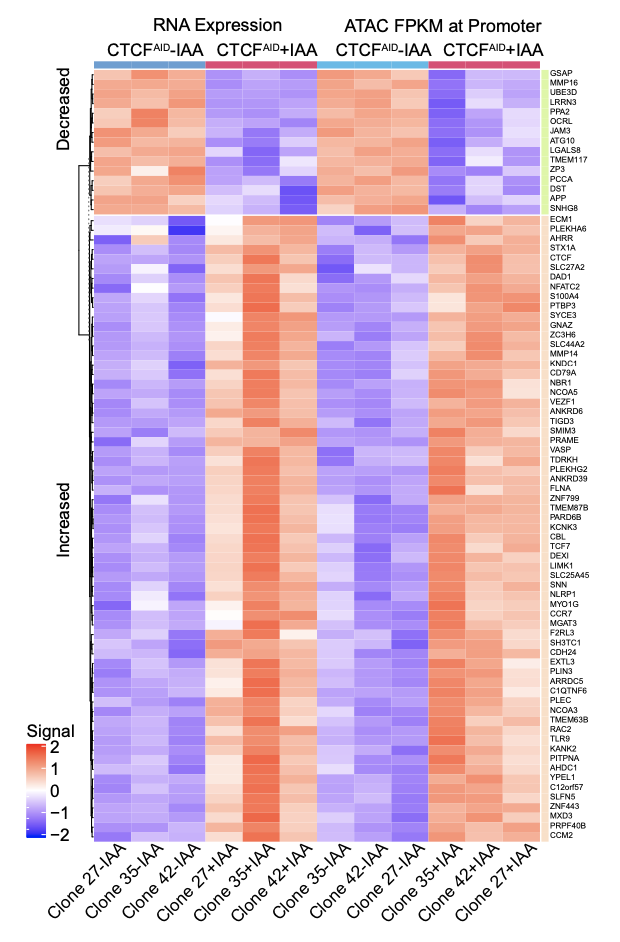


(19)    In figure 4 It would be helpful if authors could show chromatin accessibility changes at the promoter of up- and down-regulated genes (separately) in the form of a heatmap like the one presented in figure 1C, where the number of up- and down- regulated genes promoters would be clearly indicated. Figures 4C and D could go in supplementary if space becomes limiting.

Thank you for the reviewer’s suggestion. We generated the new plots as suggested.

(20)    The authors determine chromatin accessibility as well as transcriptional changes for CTCF upon CTCF depletion and infer that "CTCF can insulate itself to maintain optimal levels". In my opinion, I do not think that just Fig 4A,C, D are sufficient to draw this inference. Possibility of CTCF binding to its own promoter and autoregulating itself in a negative feedback loop can not be ruled out just based on the results presented here.

Thank you for the reviewer’s suggestion. We have revised the description in the text to include this possibility.

(21)    In Fig. 4E, authors report that the accessibility at the Myc promoter doesn't change but the accessibility at its enhancer located 1.8Mb from the promoter decreases upon CTCF depletion, leading to dramatic downregulation of Myc. However, this is at odds with the results reported in Hyle et al. 2019, wherein the authors show that CTCF binding at both Myc promoter as well as enhancer goes down. The difference between Hyle et al. 2019 and current study is the duration of depletion (48h vs. 24h). Could authors please provide insights into this?

Thank you for the reviewer’s comments. The transcriptional regulation of *MYC* is vulnerable to CTCF in the MLL-rearranged leukemia cell line SEM due to addiction of enhancer/promoter looping regulation at three-dimensional chromatin architecture. The *MYC* mRNA expression significantly reduced at both 24 and 48 hours post IAA treatment. Although the chromatin accessibility at the *MYC* promoter remains unchanged upon CTCF loss, a significant reduction of ATAC-seq signals at the CTCF binding sites located in the distal *MYC* enhancer was observed. We are working with another unpublished study to explore the molecular mechanism which indicates the CTCF loss affects the binding occupancy of enhancer-bound TFs and epigenetic regulators, further reducing the transcription of *MYC* in the 3D context of the enhancer/promoter loop.

I am curious to know if Myc promoter is one of those regions which is considered as Control DAR in the analysis presented in Fig 1 of the current study and doesn't show any changes in accessibility despite loss of CTCF binding?

Thank you for the reviewer’s comments. The *MYC* promoter has four reproducible ATAC-seq peaks. The one located closest to *MYC* TSS was significantly increased and passed FDR 5% but not passed a two-fold change. Another one downstream of *MYC* TSS was significantly increased but did not pass FDR 5%. The other two were not significantly increased or decreased but were neither included in control peaks since we required a cutoff of p-value > 0.5 for control peaks. Thus none of the four ATAC-seq peaks at *MYC* promoter were included in Fig. 1C.

And in a broader sense, did the authors find a set of deregulated genes that showed no changes in promoter and/or enhancer accessibility which has CTCF binding in the vicinity?

Thank you for the reviewer’s comments. Out of 219 up-regulated genes, only eight gene promoters have ATAC-seq control peaks and CTCF binding occupancy. Out of 269 down-regulated genes, twenty-one gene promoters have ATAC-seq control peaks and CTCF binding occupancy.

Fig 4 - Minor Comments
(22)    Is there a correlation between CTCF motif orientation and the transcription changes observed for the genes which had DARs and CTCF motif at the promoter?

Thank you for the reviewer’s comments. When we focused on 37 down-regulated genes with decreased DARs, a nearby CTCF motif was observed on the same strand of 16 (43.2%) genes. Out of 110 up-regulated genes with increased DARs, a nearby CTCF motif was observed on the same strand of 67 (60.9%) genes. It seems to indicate a mild correlation but not significant (Fisher exact test, p-value = 0.08, Odds Ratio = 0.491) due to a small size of differentially expressed genes.

Fig 6 - Major Comments
(23)    The manuscript could benefit from a clearer justification for conducting the proteome and phosphoproteome studies. Could the authors please elaborate on the logic of looking at proteome or phosphoproteome levels upon CTCF loss? Authors argue that the protein level changes are a better read out for gene expression changes rather than looking at transcriptional changes. I am unable to comprehend this, given the fact that the basis of the study is to identify chromatin accessibility changes and its correlation with transcriptional changes upon CTCF loss. While the bulk mRNA seq suffers from the limitations of differential mRNA stabilities and turn over, it would be a better idea to look at nascent transcription instead. Given how the manuscript is currently presented I fail to understand how studying the proteome or phosphoproteome help alleviate the limitation of RNA-seq.

Thank you for the reviewer’s comments. We apologize for the confusion. We did not intend to imply that protein level change is a better readout for gene expression change than mRNA. We agree that proteome or phosphoproteome did not alleviate the limitation of RNA-seq here. However, the downstream effect upon CTCF loss can be evaluated by protein level change as a complementary strategy. We revised the corresponding sentence “*these data suggest that global gene expression changes are likely more reflected in protein level other than transcriptome level*” to “*These data suggest that although the mRNA level changes are not robust, and acute CTCF loss induces substantial downstream disruption of protein expression and phosphorylation*” at line 315 in the text to avoid any confusion. We also added one more sentence in the discussion at line 433 to specify the limitation of the study: “*A limitation of the study is that the bulk mRNA sequencing may suffer from limitations of differential mRNA stabilities and turnover. Advanced nascent transcript sequencing methods, e.g., NET-seq and SLAM-seq, would be able to address this issue*.” It is widely reported that the global transcriptome and proteome correlation are often only modest, especially when cells are under acute stress (Liu et al., Cell., 2016; Zaro et al. eLife., 2020). Since the differential gene expression change reflected at the transcription level upon acute CTCF loss is lower than expected, the deep proteome and phosphoproteome studies can help us gain a broader picture of targets deregulated by acute CTCF loss at translational and posttranslational protein levels. We observed strong downstream proteome and phosphoproteome changes, which are consistent with the cellular phenotype. More importantly, we think deep proteomics and phosphoproteomics analysis are also critical for us to define and prioritize CTCF co-regulatory partners as shown in Fig. 6D.

(24)    Authors also mention that the translation machinery was also deregulated upon CTCF depletion which might have led to changes in the proteome. Doesn't this imply that the proteome change is more likely a secondary effect of their experimental system/condition.

Thank you for the reviewer’s comments. GSEA analysis from our previous study (Hyle et al. Nucleic Acids Research., 2019) suggested translation signature was enriched in SEM cells upon CTCF loss, resulting from the expression change of genes essential for translation or secondary effect upon CTCF loss. For instance, CTCF loss-induced MYC downregulation was reported to enrich deregulated translation signature. However, we do not have direct evidence to support this observation. We have revised the description in the text to avoid confusion.

(25)    The correlations between CTCF and co-regulatory partner motifs are difficult to understand as currently phrased (Fig. 6E). Are the authors suggesting that CTCF assists in co-regulatory factor binding? If so, wouldn't it be worth authors validate this by performing CUT&RUN for a few chosen co-regulatory factors upon CTCF depletion?

Thank you for the reviewer’s comments. We sought to test the co-regulatory mechanism by focusing on the CTCF and ZBTB7A. We conducted a Cut&Run assay to characterize the co-binding pattern of CTCF and ZBTB7A. However, no suitable antibodies are working for ZBTB7A in the Cut&Run assay. Due to the technical challenge and limited time for revision, we decided to test our hypothesis using publicly available data collected in a human erythroid progenitor cell line HUDEP2 (Masuda et al., Science, 2016; Liu et al., Cell, 2018). We also included RNA-seq data collected in CTCF^AID^ knockin HUDEP2 that we have previously reported (Hyle et al., Nucleic Acids Research, 2019). We identified 11,394 CTCF peaks overlapped with ZBTB7A peaks (23.5%), and the association between ATAC-seq peaks with both CTCF and ZBTB7A were strong (Heatmap below). We further tested the association with differentially expressed genes in CTCF^AID^ knockin HUDEP2 upon CTCF degradation. Interestingly, 108 genes down-regulated following CTCF degradation have both CTCF and ZBTB7A binding at the promoter (62.1%). In comparison, only 250 up-regulated genes have both CTCF and ZBTB7A binding at the promoter (39.9%, Fisher p=2.98e-7, Odds Ratio=2.468, mosaic plot below). In contrast, genes with only CTCF or only ZBTB7A were more often observed for up-regulated genes with less confidence (p=0.031 and p=0.0046). These data suggested that co-regulatory factors bound with CTCF might be indispensable for regulating the transcription of target genes.


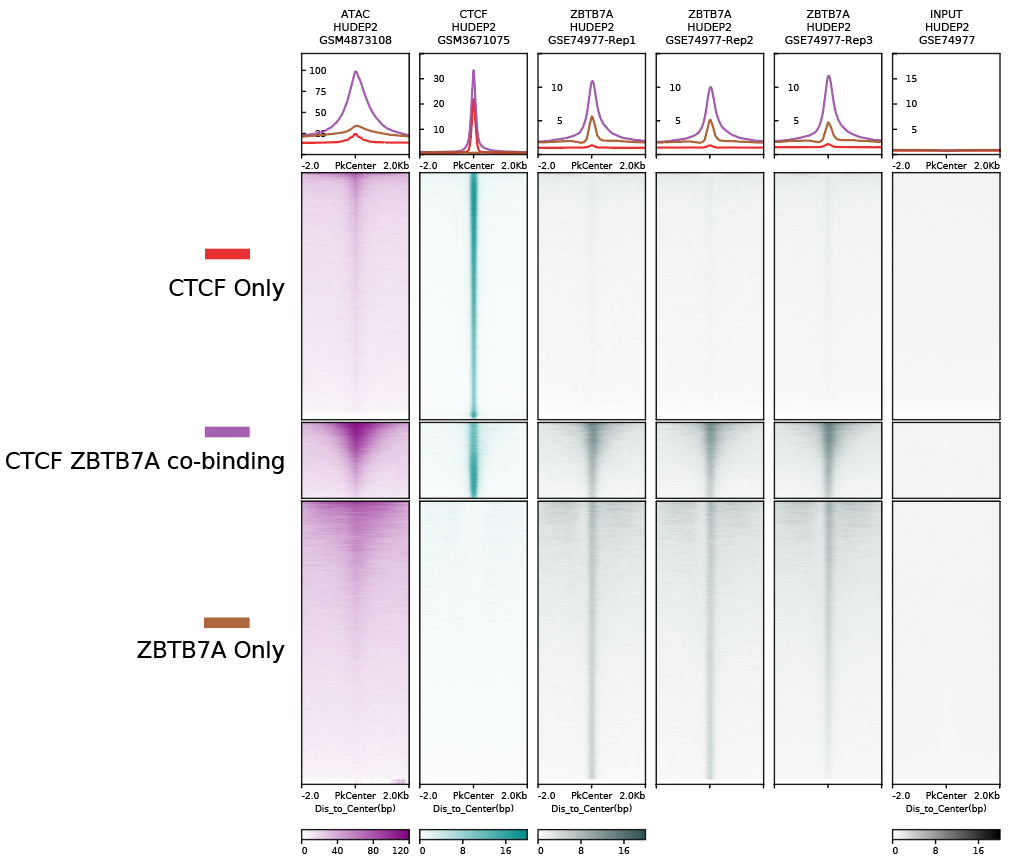


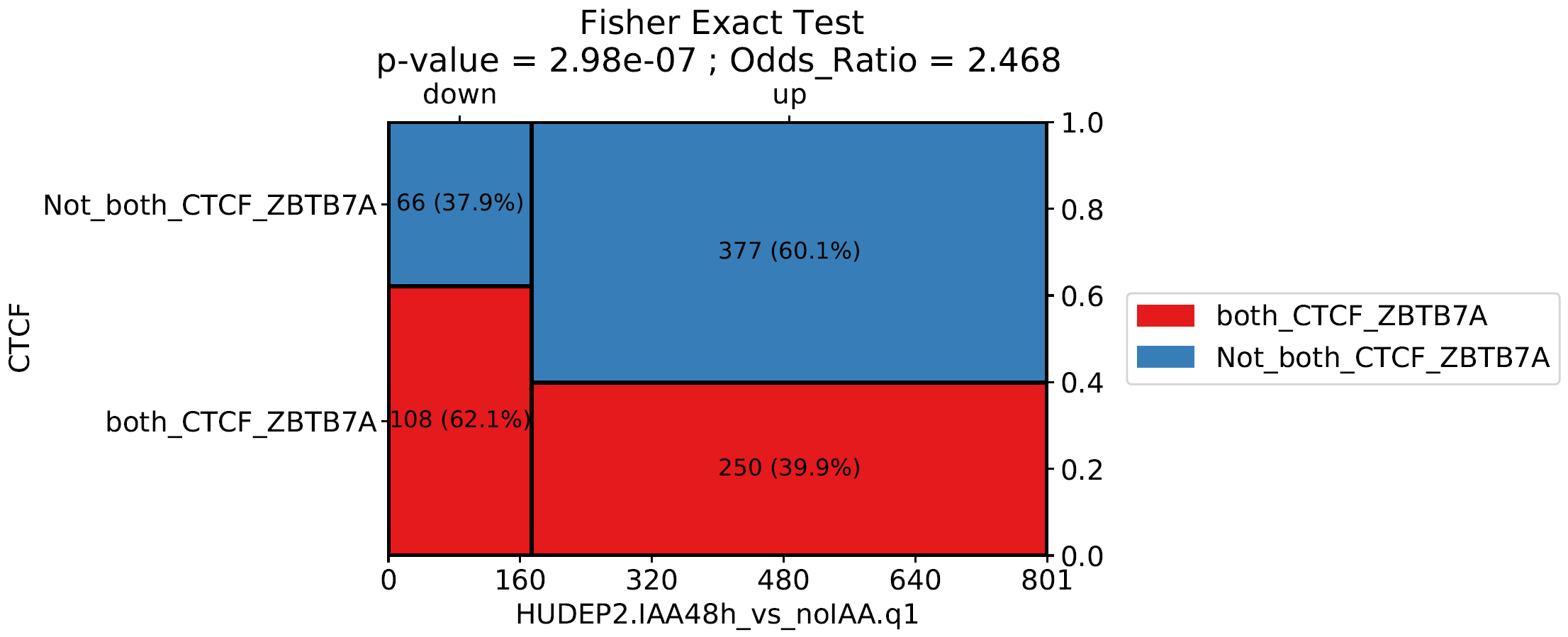


(26)    The authors posit that a correlation between the occurrence of CTCF motif and co-regulatory partner motif is more evident for Decreased DARs as compared to the Control or Increased DARs. Could the authors please clarify and elaborate upon following:
(i)     Most of decreased DARs are in the intronic regions (Fig. S5C). How many decreased DARs were incorporated in the analysis presented in Fig. 6E,F?

Thank you for the reviewer’s comments. About 6,913 decreased DARs were incorporated in the analysis in Fig. 6E with at least one CTCF motif.

(27)    (ii)In Fig 2, Increased DARs were suggested to be the regions which has motifs enriched for General TFs and are withing 100 bp of CTCF motif. And these regions are more enriched in the gene promoters. Based on this, one would expect to see much stronger correlation between the CTCF and co-regulatory factor motifs for these Increased DARs.

Thank you for the reviewer’s comments. We think even in the increased DARs with complex transcription factor binding occupancy, the transcriptional readout could be either direction. It will be challenging to correlate the CTCF and co-regulatory factor motifs for these increased DARs. We attempted to utilize multi-omics integration analysis to prioritize CTCF co-regulatory factors. We picked ZBTB7A and YY1 as examples because they were the top ones enriched for decreased DARs except aside from CTCF/Cohesin (Fig 2A) and associated with downstream-regulated genes (Fig 6D), indicated they are more likely CTCF’s immediately co-regulators.

(28)    Or do the authors think that all the CTCF sites at increased DARs are at the TAD boundary and serving as an insulator sites?

Thank you for the reviewer’s comments. We performed additional analysis and found that increased DARs were significantly distant from TAD boundaries than decreased DARs and control ATAC-seq peaks. However, we did not include these data in our current study due to the low sequencing depth for the HiC experiment. Also, these results might be limited due to 1) TADs were also experiencing dynamics upon CTCF degradation. 2) we only called high confidence TAD boundaries using the method from the previous study (Crane et al., Nature 2015), by which only 500~600 TAD boundaries were identified with confidence. Therefore, based on the current data, we could not conclude that all the CTCF sites at increased DARs are at the TAD boundary and serving as an insulator sites with high confidence.

(29)    Did the authors identify any co-repressors motifs close to the CTCF motif that might explain increased DARs upon CTCF loss and hence transcriptional upregulation?

Thank you for the reviewer’s comments. We assume ZBTB7A is one of the co-repressors since their downstream target genes were more enriched for up-regulated proteins (Fig 6D). It also has been reported recently that ZBTB7A could recruit a co-repressor complex in the biological process of globin gene switch (Science. 2016 Jan 15;351(6270):285-9). However, due to the unavailability of good CUT&RUN and ChIP-seq antibody and protocol, we did not have direct evidence to link the increased DARs and ZBTB7A binding switch.

(30)    In order to be classified as co-regulatory which is based on the correlations drawn from the data, it might be more meaningful to either deplete the co-regulatory factor(s) either individually and along with CTCF or delete the co-regulatory factor motifs from the selected sites and score for the transcriptional changes as a proof of principle.

Thank you for the reviewer’s comments. We agree with the reviewer that depleting the co-regulatory factor(s) either individually and along with CTCF will provide more information. However, given the top TF candidates are also essential survival genes, CRISPR knockout would lead to complicated secondary effects. Targeting these candidate proteins with auxin-inducible degron similar to CTCF^AID^ will be the only practical approach to address this question. Due to the tremendous efforts to generate these tools, we will not perform these experiments in this study. We will be interested in developing such research tools in the future.

Fig 6 - Minor Comments
(31)    Is there a significant overlap between proteome and phosphoproteome? If so, what does that mean in the context of CTCF depletion?

Thank you for the reviewer’s comments. We observed a modest overlap between differentially expressed (DE) phosphoproteome and proteome, similar to what we have observed in other cancer studies (Wang et al., Nature Communications, 2019; Stewart et al., Cancer Cell, 2018). Out of the 1,269 proteins with differentially expressed phosphor sites and 2,550 differentially expressed total proteins, we identified 551 overlapped proteins, equal to 43% of the DE phosphorylation and 22% of DE proteins.


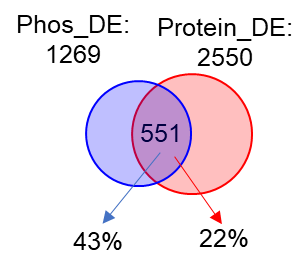


General Minor Comments
(32)    Page 3, line 33 - Not just human cohesin, also shown in mouse cells (mESC, CH12)

Thank you for the reviewer’s suggestion. We have included this research progresses.

(33)     Page 3, line 36 - Change Ref 19 (Nora et al. 2020, Nat Comm)

Thank you for the reviewer’s suggestion. We have updated the latest reference.

(34)    Page 18, line 376 -There seems to be a mistake. The statement should be "increased" DARs upon CTCF loss exhibit tandem CTCF-binding pattern.

Thank you for the reviewer’s comments. We apologize for the confusion. We think these results might be due to the difference between the tandem CTCF-binding occupancy and tandem CTCF motifs (2xCTS). The tandem CTCF-binding pattern was observed in decreased DARs (Fig 1C, Sup Fig 2C, and Sup Fig 3A). The estimated distance between the two sites was about 200bp. We were not sure whether increased DARs might also have tandem CTCF-binding patterns. Because of the complex TF binding at promoters, it is challenging to find the summit of the chromatin accessibility region to achieve enough resolution for the observation. On the other hand, the tandem CTCF motifs (2xCTS) were enriched for increased DARs. The reported distance between the two sites was about 33bp and enriched for BORIS/CTCFL binding.

(35)    Page 21, lines 440, 447 - What do authors mean by occupancy switching? They cite an example of switching between CTCF and YY1 (line 447) but that doesn't seem likely considering the fact that CTCF and YY1 binding motifs are absolutely unique. However, CTCF and YY1 do interact with each other, so it is feasible that they are found at each other's motifs but that might not be the idea here.

Thank you for the reviewer’s comments. We apologize for the confusion. In the discussion section, we sought to propose the possible mechanism that loss of CTCF affects YY1 binding occupancy. We also agree with the reviewer that CTCF and YY1 do interact with each other. To avoid potential confusion, we will delete this piece of discussion in the text.

(36)    In Supp Fig. 5, DAR numbers for the Venn diagrams slightly different from Fig 1C

Thank you for the reviewer’s comments. The number in Supp Fig.5 was the actual number of peaks that passed the threshold. To get a better resolution for the heatmap and avoid artifacts due to two very close peaks, we used the summit of peaks to generate the heatmap. We have provided details to define these steps more clearly in the method part. In addition, we find a few incorrect numbers in the text, Fig 1C, and Fig 6B. We have now corrected all those errors.

(37)    Line 66 should read "our data" instead of "we data"

We apologize for the typo. It was corrected throughout the text.

(38)    Line 67 should read "shedding light" not "a light"

We apologize for the typo. It was corrected throughout the text.

**Second round of review**

**Reviewer 1**

The authors have addressed my comments. It is still unclear what role ZBTB7A could play in the CTCF biology, unfortunately.

The CRISPR experiments nicely show an insulating function for the CTCF site upstream of BLCAP.

**Reviewer 2**

The authors have addressed and clarified most of the concerns raised and the current version of the manuscript reads better. The findings are very relevant and of interest to the field. The authors provide a detailed and well-reasoned explanation to the comments raised. However, a lot of this is still not a part of the manuscript text. I feel that this would be crucial and helpful for the readers and would therefore, highly recommend following edits before final acceptance:
(1)     CRISPR-drop out experiment is now part of the Supplementary Figure and has been explained with the help of a schematic. However the Methods section is not nearly detailed enough to allow anyone to reproduce these experiments. More details about the experimental and computational procedures must be reported.

(2)     Please acknowledge the presence of residual CTCF and how would that not affect the analysis presented. It is clear in the drafted response but not included in the manuscript text.

(3)     Correlation of increased/decreased DARs with the TAD boundary is interesting. I agree with the authors on the limited scope of this analysis given the low sequencing depth of HiC libraries and other confounding factors listed. I still think it might be worthwhile to provide a speculation on their observation in Fig. 2F.

(4)     Logic of using USF1/2 as Controls is still not specified in the manuscript.

(5)     Include explanation about CTCF occupancy and accessibility discrepancy at the Myc promoter and enhancer in the text. Author response to the comment 21.

(6)     It would be helpful to include the following in the text or show it as a part of the Supp figure.
“And in a broader sense, did the authors find a set of deregulated genes that showed no changes in promoter and/or enhancer accessibility which has CTCF binding in the vicinity?”
Thank you for the reviewer’s comments. Out of 219 up-regulated genes, only eight gene promoters have ATAC-seq control peaks and CTCF binding occupancy. Out of 269 down-regulated genes, twenty-one gene promoters have ATAC-seq control peaks and CTCF binding occupancy.

(7)     Owens et al. is now cited but not discussed at all in the context of results presented here. For instance, the findings in Owens et al. can lend support to the potential role of CTCF in changing chromatin accessibility and binding of other GTFs (Lines 156-157, 446-449).

**Authors’ response to reviewers**

**Reviewer #1**

The authors have addressed my comments. It is still unclear what role ZBTB7A could play in the CTCF biology, unfortunately.

Thank you for the reviewer’s comments. We agree with this reviewer that it is crucial to investigate the role of ZBTB7A in CTCF biology. Given ZBTB7A is a survival essential gene similar to CTCF, the best cellular model will be an auxin-induced degron system that requires extensive efforts based on our experience. We will continue to establish such materials and investigate the co-regulatory function in the future.

The CRISPR experiments nicely show an insulating function for the CTCF site upstream of BLCAP.

Thank you for the reviewer’s positive comments.

**Reviewer #2**

The authors have addressed and clarified most of the concerns raised and the current version of the manuscript reads better. The findings are very relevant and of interest to the field. The authors provide a detailed and well-reasoned explanation to the comments raised. However, a lot of this is still not a part of the manuscript text. I feel that this would be crucial and helpful for the readers and would therefore, highly recommend following edits before final acceptance:

(1) CRISPR-drop out experiment is now part of the Supplementary Figure and has been explained with the help of a schematic. However the Methods section is not nearly detailed enough to allow anyone to reproduce these experiments. More details about the experimental and computational procedures must be reported.

Thank you for the reviewer’s comments. We have now provided experimental and computational details in the method section (see lines 624-633).

(2) Please acknowledge the presence of residual CTCF and how would that not affect the analysis presented. It is clear in the drafted response but not included in the manuscript text.

Thank you for the reviewer’s comments. We have added a paragraph to acknowledge the presence of residual CTCF and to explain why it would not affect the analysis in the revised manuscript (see lines 496-505).

(3) Correlation of increased/decreased DARs with the TAD boundary is interesting. I agree with the authors on the limited scope of this analysis given the low sequencing depth of HiC libraries and other confounding factors listed. I still think it might be worthwhile to provide a speculation on their observation in Fig. 2F.

Thank you for the reviewer’s comments. We added a sentence to discuss this piece of data (see lines 192-194). “It is known that TAD boundaries are enriched in CTCF binding sites and transcriptional active genes including housekeeping genes. Physical location of CTCF occupancy seems to be closely associated with its transcriptional regulation.”

(4) Logic of using USF1/2 as Controls is still not specified in the manuscript.

Thank you for the reviewer’s comments. We added a sentence to discuss the rationale using USF1/2 as controls in the text. “Given the USF1/2 and CTCF binding consensus motifs are completely different, we also included DARs collected from USF1/2 knockdown to support the conclusion that CTCF regulated DARs were specific to CTCF loss” (see lines 100-103).

(5) Include explanation about CTCF occupancy and accessibility discrepancy at the Myc promoter and enhancer in the text. Author response to the comment 21.

Thank you for the reviewer’s comments. We have now included this paragraph in the text (see lines 246-252 and 255-263).

(6) It would be helpful to include the following in the text or show it as a part of the Supp figure. “And in a broader sense, did the authors find a set of deregulated genes that showed no changes in promoter and/or enhancer accessibility which has CTCF binding in the vicinity?” Thank you for the reviewer’s comments. Out of 219 up-regulated genes, only eight gene promoters have ATAC-seq control peaks and CTCF binding occupancy. Out of 269 down-regulated genes, twenty-one gene promoters have ATAC-seq control peaks and CTCF binding occupancy.

Thank you for the reviewer’s comments. We have now included the following description in the text (see lines 234-239). Out of 219 up-regulated genes, only eight gene promoters have ATAC-seq control peaks and CTCF binding occupancy. Out of 269 down-regulated genes, twenty-one gene promoters (24 peaks) have ATAC-seq control peaks and CTCF binding occupancy. However, given the small number of peaks, we would not show the data in a separate figure.


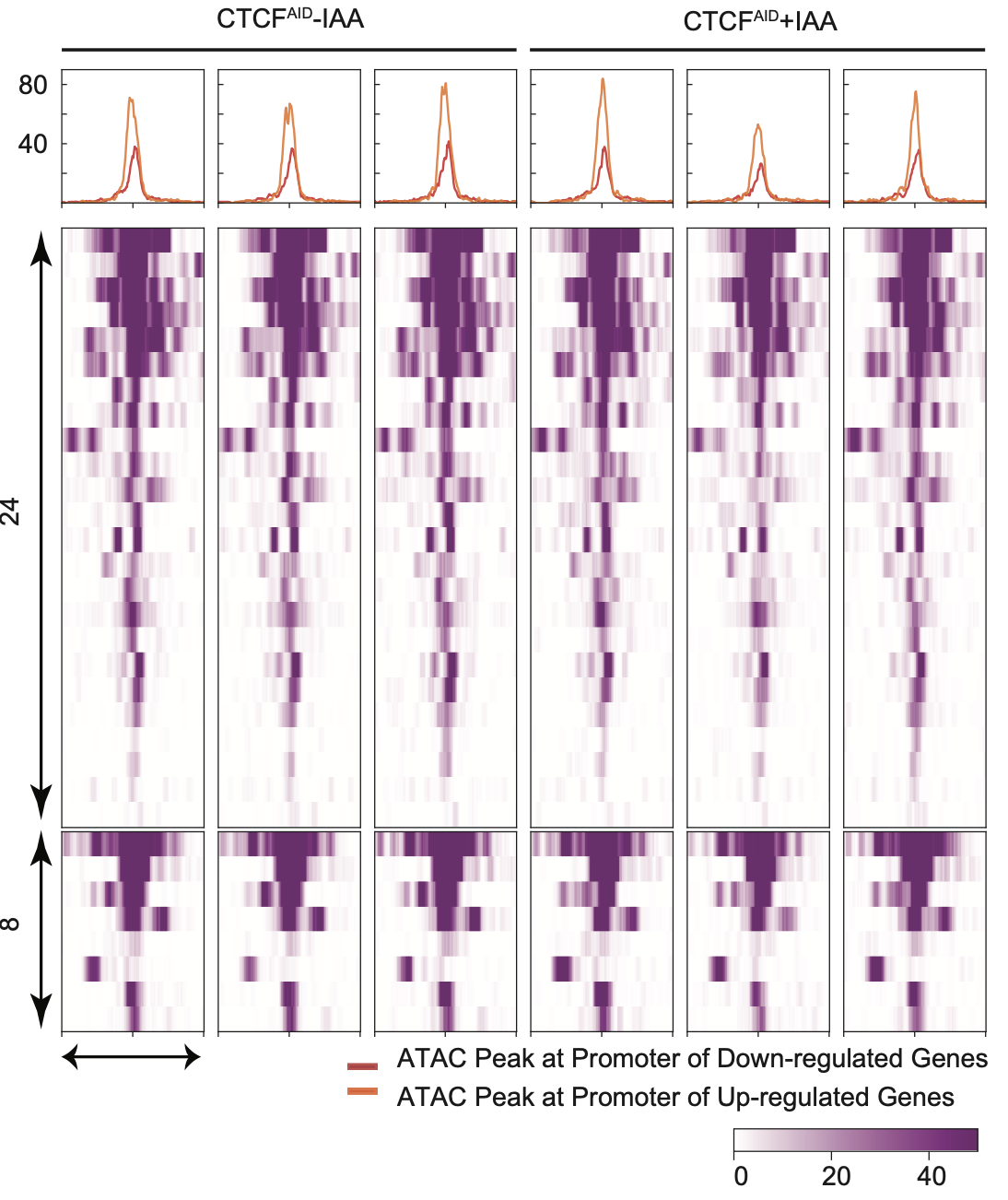


(7) Owens et al. is now cited but not discussed at all in the context of results presented here. For instance, the findings in Owens et al. can lend support to the potential role of CTCF in changing chromatin accessibility and binding of other GTFs (Lines 156-157, 446-449).

Thank you for the reviewer’s comments. We agree with this reviewer that Owens et al. can lend support to the potential role of CTCF in changing chromatin accessibility and binding of other GTFs. We have now included the discussion in the text (see lines 428-431).
